# Detection of adverse drug events in e-prescribing and administrative health data: a validation study

**DOI:** 10.1186/s12913-021-06346-y

**Published:** 2021-04-23

**Authors:** Bettina Habib, Robyn Tamblyn, Nadyne Girard, Tewodros Eguale, Allen Huang

**Affiliations:** 1 Clinical and Health Informatics Research Group, McGill University, 1140 Pine Avenue West, Montreal, QC H3A 1A3 Canada; 2 Department of Epidemiology, Biostatistics, and Occupational Health, McGill University, Montreal, Canada; 3 Department of Medicine, McGill University Health Centre, Montreal, Canada; 4 School of Pharmacy, Massachusetts College of Pharmacy and Health Sciences, Boston, MA USA; 5 Division of Geriatric Medicine, University of Ottawa, Ottawa, Ontario Canada

**Keywords:** Adverse drug event, Administrative health data, Electronic prescribing data, Validation

## Abstract

**Background:**

Administrative health data are increasingly used to detect adverse drug events (ADEs). However, the few studies evaluating diagnostic codes for ADE detection demonstrated low sensitivity, likely due to narrow code sets, physician under-recognition of ADEs, and underreporting in administrative data. The objective of this study was to determine if combining an expanded ICD code set in administrative data with e-prescribing data improves ADE detection.

**Methods:**

We conducted a prospective cohort study among patients newly prescribed antidepressant or antihypertensive medication in primary care and followed for 2 months. Gold standard ADEs were defined as patient-reported symptoms adjudicated as medication-related by a clinical expert. Potential ADEs in administrative data were defined as physician, ED, or hospital visits during follow-up for known adverse effects of the study medication, as identified by ICD codes. Potential ADEs in e-prescribing data were defined as study drug discontinuations or dose changes made during follow-up for safety or effectiveness reasons.

**Results:**

Of 688 study participants, 445 (64.7%) were female and mean age was 64.2 (SD 13.9). The study drug for 386 (56.1%) patients was an antihypertensive, and for 302 (43.9%) an antidepressant. Using the gold standard definition, 114 (16.6%) patients experienced an ADE, with 40 (10.4%) among antihypertensive users and 74 (24.5%) among antidepressant users. The sensitivity of the expanded ICD code set was 7.0%, of e-prescribing data 9.7%, and of the two combined 14.0%. Specificities were high (86.0–95.0%). The sensitivity of the combined approach increased to 25.8% when analysis was restricted to the 27% of patients who indicated having reported symptoms to a physician.

**Conclusion:**

Combining an expanded diagnostic code set with e-prescribing data improves ADE detection. As few patients report symptoms to their physician, higher detection rates may be achieved by collecting patient-reported outcomes via emerging digital technologies such as patient portals and mHealth applications.

**Supplementary Information:**

The online version contains supplementary material available at 10.1186/s12913-021-06346-y.

## Background

Drug expenditures have been growing at a faster rate than all other costs in health care [1, 2]. While modern drug therapy plays an important role in managing health problems, adverse drug events (ADEs) are frequent and costly, with up to 16% of emergency department (ED) visits and 7% of hospital admissions being medication-related [3–6]. The frequency of these events can be explained in part by established processes for drug approval, which test drugs in tightly controlled settings and in a limited number of patients who infrequently represent those typically prescribed the drug after approval. In addition, once drugs have entered the market, they are often prescribed off-label for conditions for which they have not been approved [7–10], which previous research has shown increases the risk of ADEs [11]. Consequently, robust methods of post-marketing surveillance have emerged as a requirement to monitor the safety and effectiveness of medications after they have been approved for sale [12–14].

Methods of post-marketing drug surveillance include voluntary systems for spontaneous reporting of ADEs [15] and prescription event monitoring [16], both of which suffer from systematic under-reporting [16–19]. The data gaps in these reporting systems have been increasingly supplemented with computerized administrative health data, which are timelier, more reliable, and easier to collect. Diagnostic codes from physician billing data are often used to detect ADEs, with International Classification of Disease (ICD) code sets developed for this purpose [20–30]. However, most of these codes have only been validated in the in-patient hospital setting [21, 22, 24–26, 31]. Few studies have evaluated the accuracy of ICD code sets for ADE detection in ED and outpatient settings, where the majority of medications are prescribed and resulting ADEs diagnosed [32]. Moreover, code sets evaluated in ED and outpatient settings have not been very helpful, with a reported sensitivity of only up to 28% in detecting ADEs [20, 23, 27, 28]. Many codes are external cause codes that describe precise causes of ADEs (e.g. ICD-10 Y40-Y59 drugs, medication, and biological substances causing adverse effects in therapeutic use) or codes that relate to drug-induced diagnoses (e.g. ICD-10 G44.4 drug-induced headache). ADE detection which relies on these codes requires that the ADE is both recognized by the busy clinician and coded and recorded in administrative health data, activities which are sub-optimally achieved [4, 20, 33, 34].

In contrast, automated systems that search electronic medical notes for phrases representing known adverse effects of specific drug classes are much better at detecting ADEs [23, 35]. This approach relies on the documentation of patient symptoms, which is routinely done, rather than on a clinician’s recognition of an ADE. To develop an enhanced ADE detection system for use in administrative health data, we adapted the strategy of searching for drug side effects by creating an expanded, therapeutic class-targeted ICD code set. This code set includes side effects of specific therapeutic drug classes (e.g. sexual dysfunction for antidepressants), in addition to the conventional external cause codes and drug-induced diagnoses.

One missed opportunity for ADE detection is the use of data from computerized prescribing systems. These systems are ideal for identifying when new drugs are started, when doses are changed, or when drugs are discontinued because of adverse effects or lack of therapeutic effectiveness [36, 37]. Moreover, electronic prescribing systems are commonly used, if not required, in many jurisdictions and the majority of these systems provide highly structured drug data. To further capture ADEs recognized by physicians but underreported in administrative data, we took advantage of an e-prescribing system that requires physicians to record the reasons for changing dose or discontinuing a drug – data that have previously been shown to be accurately recorded – to determine if e-prescribing data could increase ADE detection.

We assessed the accuracy of an expanded administrative data ICD code set and e-prescribing data, individually and combined, for ADE detection among patients receiving a newly prescribed medication in primary care.

## Methods

### Research design

We conducted a prospective cohort study to assess the sensitivity, specificity, positive predictive value (PPV), and negative predictive value (NPV) of using treatment change orders in e-prescribing data and diagnostic codes in administrative health data to detect adverse events attributable to medications prescribed in a primary care setting. We assessed the accuracy of ADE detection among new users of antidepressant and antihypertensive medications, as these are among the mostly frequently prescribed medication classes and account for a significant proportion of adverse events [8, 23, 38–41]. New users were identified through the e-prescribing system, which had information on all drugs prescribed and dispensed in the past year. New users were then followed for a period of 2 months, starting from the date the medication was first dispensed. Gold standard ADEs were defined as new or worsening symptoms reported by patients and adjudicated as medication-related by a clinical expert using the Naranjo criteria [42].

### Study context

This study was conducted in Quebec, Canada, where the healthcare system provides complete coverage for medical services. Prescription drug coverage in Quebec is mandatory and provided by the provincial health insurance agency (Régie de l’assurance maladie du Québec – RAMQ) to individuals who are above the age of 65, are welfare recipients, or are not covered by their employer.

**Fig. 1:**
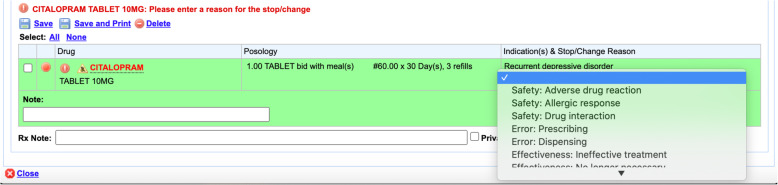
MOXXI stop/change option: indicating the reason for changing or discontinuing a medication

Patients were recruited into the study between 2007 and 2015 through MOXXI (Medical Office of the XXIst Century), an experimental electronic health record platform used by 110 primary care physicians for approximately 90,000 of their patients [43]. The system, which integrates information drawn in real-time from administrative health databases into patient profiles (e.g. medications dispensed, current and past health problems, recent ED visits and hospital admissions), also functions as an e-prescribing tool. Physicians using MOXXI must enter a treatment indication for every new medication prescribed by selecting an item from a drop-down listing of all known on- and off-label conditions for that drug. If a medication is discontinued or its dose is changed, the prescriber is required to record a reason for their action by selecting an option from a drop-down listing that includes: adverse drug reaction, ineffective treatment, drug interaction, allergic response, adjusting dose to optimize treatment, error in prescribing, incorrect medication dispensed, end of treatment, and substitution for less expensive drug (Fig. 1). The medication change and associated reason are also printed on the prescription and recorded in the treatment history of the health problem for which the drug was prescribed. A previous study showed that MOXXI had a specificity of 99.7% and sensitivity of 67.0% in documenting treatment discontinuations and dose changes, as well as high concordance between treatment change reasons recorded in the application and those obtained from chart-facilitated physician interview [37].

### Study population

Patients participating in MOXXI were eligible to be included in this study if they 1) had prescription drug insurance through the RAMQ, 2) were prescribed and dispensed an antidepressant or antihypertensive medication by a MOXXI physician between 2007 and 2015, and 3) had no prior prescription or record of dispensed medication for that same drug in the preceding 12 months. We chose to focus on incident users as adverse events are more likely to occur shortly after starting or stopping therapy [44–47].

After enrollment in the study, patients were followed for 2 months, starting from the date the antidepressant or antihypertensive medication was first dispensed. During this period, data were collected on medical services received, dose changes or discontinuations of the study drug, and patient-reported outcomes.

### Data sources

#### Administrative health data

Real-time patient demographic, drug, and clinical information was gathered using MOXXI’s dedicated, secure interface to RAMQ databases, with records linked using patients’ unique health insurance numbers. Patient demographic information included date of birth, sex, and date of death (if applicable). Drug information included pharmacy claims data for all drugs dispensed to a patient following the visit in which they received the new prescription, as well as in the preceding 12 months. This included the date of dispensing, drug identification number, quantity dispensed, and prescription duration. The validity of these drug data has been previously demonstrated [48].

Medical fee-for-service billing data for services provided by any licensed physician in the province to study patients during the 2-month follow-up period and the 12 months prior to enrollment were also obtained via the RAMQ interface. These data included the date of service delivery, the service location (critical care, inpatient ward, ED, outpatient clinic, private office), and the ICD-9 diagnosis for the visit. In addition, data from the provincial hospitalization database were retrieved and linked to study patients by unique medicare number. For each hospitalization during the follow-up period and the 12 months prior, we retrieved admission and discharge dates, ICD-10 codes of the principal discharge diagnosis, length of stay, and the discharge destination (e.g. death, home, rehabilitation).

#### MOXXI electronic prescribing data

Data from the MOXXI e-prescribing tool were used to identify patients with new prescriptions of antihypertensive and antidepressant medications between 2007 and 2015. Prescription information included the patient’s unique health insurance number, date of the prescription, drug identification number, treatment indication, quantity, duration, directives, and number of refills. For each patient with a new prescription for one of the study drugs, we also retrieved data on subsequent discontinuations or dose changes made during the 2 months following first dispensation of the drug, along with the documented reason(s) for the change.

#### Patient interview data

A modified version of the Australian adverse reaction and drug event report was used to collect patient feedback on adverse events (Additional file 1: Patient Questionnaire) [49]. The interview was administered via telephone by a trained research assistant within 3 weeks following the first dispensation of the study drug. Patients were first asked to report any new or worsening symptom or health problem experienced in the past 3 weeks. Standardized system-related questions were then used to identify any body system changes patients may have experienced since starting the drug, and drug-specific standardized questions were used to determine if patients had experienced any of the known effects of the study drug.

### Patient characteristics

Demographic and clinical characteristics used to characterize the study population included age, sex, the therapeutic class of the study drug, the treatment indication of the study drug, the number of concurrent medications patients were taking, and the presence or absence of the 17 conditions included in the Charlson Comorbidity Index [50, 51]. The latter was determined using diagnostic codes for medical services that patients received in the 12 months prior to enrollment in this study.

### Adverse drug event (ADE) definitions

#### ADEs in administrative health data: Expanded therapeutic class-targeted code set & standard code set

The expanded, therapeutic class-targeted ICD code set was composed of ICD codes for known adverse effects of antihypertensive and antidepressant medications (e.g. sexual dysfunction for antidepressants), as well as external cause codes specifying an adverse reaction to those medication classes. Adverse effects for every drug group have been defined by the Oregon Drug Effectiveness Review Project [52] and those relevant to antidepressants and antihypertensives were mapped to corresponding ICD-9 and ICD-10 codes (Additional file 2: Appendices 1–2).

The narrower, standard code set consisted of diagnostic codes included in previously reported ADE code sets [20, 23, 27, 28]. This code set was comprised of external cause codes and drug-induced diagnoses specifying an adverse reaction to antihypertensive and antidepressant medications (Additional file 2: Appendix 3).

To assess ADEs using the standard and expanded code sets, ICD codes recorded in medical services claims for all physician visits (office, ED, outpatient, inpatient) and hospital admissions during the follow-up period were retrieved. Patients were classified as having a potential ADE if one or more ICD codes from the a) standard code set or b) expanded code set were documented as a reason for the physician visit or hospital admission.

#### ADEs in MOXXI electronic prescribing data

Potential ADEs in MOXXI e-prescribing data were defined as study drug discontinuations or dose changes made by the study physician during the follow-up period for safety (adverse drug reaction, allergy, drug interaction) or effectiveness reasons (treatment ineffective). Treatment changes made for ‘ineffectiveness’ reasons could indicate underlying problems related to medication safety, such as interactions, and because patients often discontinue medication due to adverse reactions [53–55].

#### Gold standard assessment of ADEs

New or worsening symptoms and health problems reported during patient interviews were reviewed by a clinical expert. The expert was also presented with information regarding other medications the patient was prescribed and dispensed, the patient’s medical problem list as documented in MOXXI, and medical services received by the patient (ED visits and hospital admissions). The Naranjo criteria for ADE assessment were used to assess the likelihood that the symptom(s) reported during the interview were related to the newly started therapy [42]. The Naranjo algorithm assesses the presence or absence of ten criteria related to the adverse event, each of which is given a specific weight based on its relative importance. The sum of weights is then used to determine an overall score, which places the probability that the event was medication-related into one of four categories: doubtful, possible, probable, or definite. Gold standard ADEs were defined as patient-reported adverse events whose Naranjo score was classified as probable or definite.

### Outcome definitions

Sensitivity was defined as the proportion of true ADEs, as defined by the gold standard, which were correctly identified using e-prescribing and/or administrative health data. Specificity was defined as the proportion of true negatives that were identified as such using e-prescribing and/or administrative health data. The PPV was defined as the proportion of potential ADEs identified using e-prescribing and/or administrative health data that were true ADEs, and the NPV as the proportion of patients who did not have an ADE based on e-prescribing and/or administrative health data who were true negatives.

### Statistical analysis

Descriptive statistics were used to characterize the study population and to estimate ADE rates, overall and by therapeutic class, using the gold standard definition, standard and expanded ICD code sets, and MOXXI e-prescribing data. The sensitivity, specificity, PPV and NPV of using administrative health data and e-prescribing data, individually and combined, to detect ADEs were also estimated, overall and by therapeutic class. 95% confidence intervals (CIs) around these estimates were constructed using the exact method for binomial proportions. All analyses were conducted using SAS version 9.4 (SAS Institute Inc., Cary, NC, USA).

## Results

### Study cohort

**Fig. 2:**
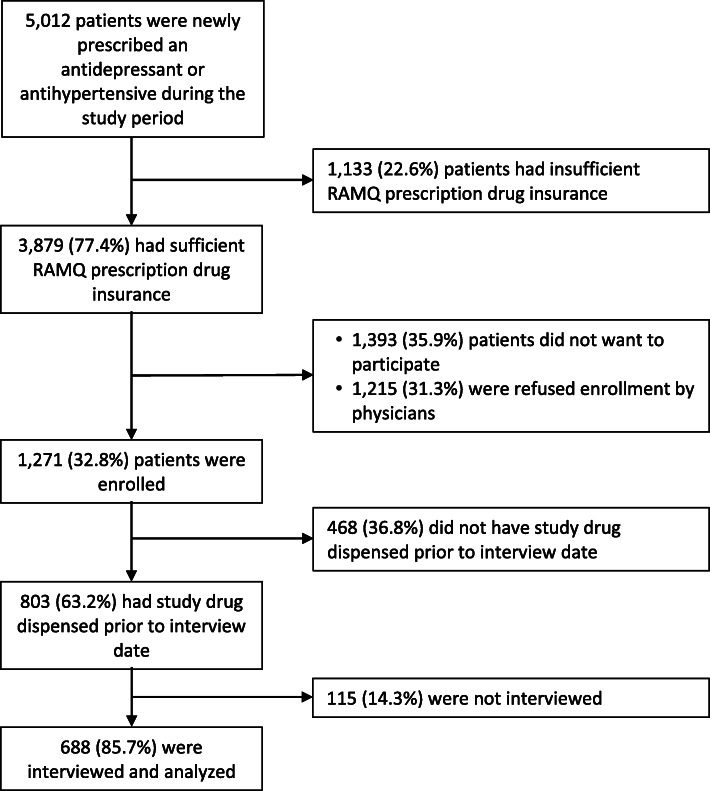
CONSORT diagram of eligible, enrolled, and interviewed patients

Between July 2007 and June 2015, 5012 patients had a new prescription for an antihypertensive or antidepressant medication (Fig. 2). Of these, 1133 (22.6%) had insufficient RAMQ prescription drug coverage during the study period, 1393 (27.8%) declined to participate, and 1215 (24.2%) were refused enrollment by the MOXXI physician. A total of 1271 patients consented and were enrolled in the study. Compared to patients who declined to participate, these patients were younger (mean age 64.1 vs 67.0), a larger proportion of them were male (37.2% vs 34.2%), and a smaller proportion had an indication of depression if prescribed an antidepressant (40.5% vs 44.6%) (Additional file 2: Appendix 4). Among enrolled patients, 803 (63.2%) had the study drug dispensed and a subsequent 688 (85.7%) patients completed the interview within 3 weeks of medication dispensation and were included in the analysis.

### Patient characteristics

The majority (*N* = 445, 64.7%) of study participants were female and the mean age was 64.2 years (SD 13.9 years) (Table 1). The study drug for 386 (56.1%) patients was an antihypertensive medication, and for 302 (43.9%) patients it was an antidepressant. The most frequently prescribed antihypertensive drug classes were calcium channel blockers (30.1%) and angiotensin II receptor antagonists (29.3%), and the most common antidepressant classes were selective serotonin reuptake inhibitors (SSRIs, 38.7%) and serotonin-norepinephrine reuptake inhibitors (SNRIs, 34.4%). The most common recorded treatment indications for antihypertensive prescriptions were hypertension (79.3%) and edema (4.7%). For antidepressants, depression (40.1%), generalized anxiety disorder (20.5%), and insomnia (17.2%) were the most common treatment indications.

**Table 1:** Characteristics of Study Patients

	All Study Patients (*N* = 688)	Patients Prescribed an Antidepressant (*N* = 302)	Patients Prescribed an Antihypertensive (*N* = 386)
**Age, mean (SD)**	64.2 (13.9)	59.5 (16.2)	67.8 (10.6)
**Sex (male)**	243 (35.3%)	94 (31.1%)	149 (38.6%)
**Most Commonly Prescribed Drug Classes**			
*Antidepressants*			
SSRIs	117 (17.0%)	117 (38.7%)	NA
SNRIs	104 (15.1%)	104 (34.4%)	NA
SRIs	50 (7.3%)	50 (16.6%)	NA
*Antihypertensives*			
Calcium Channel Blockers	116 (16.9%)	NA	116 (30.1%)
Angiotensin II Receptor Blockers	113 (16.4%)	NA	113 (29.3%)
Thiazide Diuretics	50 (7.3%)	NA	50 (13.0%)
**Most Commonly Prescribed Medications**			
*Antidepressants*			
Citalopram	90 (13.1%)	90 (29.8%)	NA
Trazodone^a^	50 (7.3%)	50 (16.6%)	NA
Venlafaxine	44 (6.4%)	44 (14.6%)	NA
*Antihypertensives*			
Amlodipine	69 (10.0%)	NA	69 (17.9%)
Hydrochlorothiazide	45 (6.5%)	NA	45 (11.7%)
Telmisartan	32 (4.7%)	NA	32 (8.3%)
**Most Common Therapeutic Indications**			
*Antidepressants*			
Depression	121 (17.6%)	121 (40.1%)	NA
Generalized Anxiety Discorder	62 (9.0%)	62 (20.5%)	NA
Insomnia	52 (7.6%)	52 (17.2%)	NA
*Antihypertensives*			
Hypertension	306 (44.5%)	NA	306 (79.3%)
Oedema	18 (2.6%)	NA	18 (4.7%)
**Number of Concurrent Medications**^**b**^	3.7 (2.9)	3.8 (2.9)	3.7 (2.9)
**Charlson Comorbidity Index Score**			
0	334 (48.5%)	165 (54.6%)	169 (43.8%)
1	166 (24.1%)	73 (24.2%)	93 (24.1%)
2+	188 (27.3%)	64 (21.2%)	124 (32.1%)
**Most Common Comorbidities**			
Chronic Pulmonary Disease	149 (21.7%)	68 (22.5%)	81 (21.0%)
Diabetes (without complications)	105 (15.3%)	36 (11.9%)	69 (17.9%)
Renal Disease	66 (9.6%)	21 (7.0%)	45 (11.7%)
Congestive Heart Failure	58 (8.4%)	18 (6.0%)	40 (10.4%)
Cerebrovascular Disease	48 (7.0%)	20 (6.6%)	28 (7.3%)

Unless otherwise specified, all estimates are presented as N (%)

*Abbreviations*: *SSRIs* Selective Serotonin Reuptake Inhibitors, *SNRIs* Serotonin-Norepinephrine Reuptake Inhibitors, *SRIs* Serotonin Reuptake Inhibitors, *NA* Not applicable

^a^96% of prescriptions for trazodone were for an indication of insomnia

^b^Defined as the number of medications besides the study drug that were dispensed in the 1 month following the visit in which the study drug was prescribed

More than half (51.5%) of patients in this study had at least one comorbidity, with a larger proportion of antihypertensive users having a comorbidity (56.2%) compared to antidepressant users (45.4%) (Table 1). The most common comorbidities included chronic pulmonary disease (21.7%), diabetes (without complications) (15.3%), and renal disease (9.6%). In the one month following the visit in which the study drug was prescribed, patients were dispensed an average of 3.7 (SD 2.9) medications other than the study drug (Table 1). There was no difference between antidepressant and antihypertensive users in the number of medications dispensed.

### ADE incidence

Of the 688 study participants, 318 (46.2%) reported at least one new or worsening symptom or health problem during the interview. Among patients prescribed an antihypertensive, 150 (38.9%) reported symptoms, the most common of which were headache (17.3%), dizziness (17.3%) and fatigue (16.0%). More than half (*N* = 168, 55.6%) of patients prescribed an antidepressant reported symptoms, among which dry mouth or throat (20.2%), headache (18.5%), and nausea or vomiting (17.9%) were the most common (Additional file 2: Appendix 5).

Using the gold standard definition, 114 (16.6%) study patients experienced an adverse event attributable to the study drug during the study follow-up period (Table 2). Among patients prescribed an antihypertensive, 40 (10.4%) experienced an ADE, compared with 74 (24.5%) patients prescribed an antidepressant who experienced an ADE.

**Table 2:** Adverse Drug Event (ADE) Rates, by Definition and Data Source

Data Source	ADE in all Study Patients (N = 688)	ADE in Patients Prescribed an Antidepressant (N = 302)	ADE in Patients Prescribed an Antihypertensive (N = 386)
Gold Standard^a^	114 (16.6%)	74 (24.5%)	40 (10.4%)
Electronic Prescribing Data, Treatment Change Orders^b^	40 (5.8%)	20 (6.6%)	20 (5.2%)
Administrative Health Data, Expanded ICD Code Set^c^	62 (9.0%)	11 (3.6%)	51 (13.2%)
Administrative Health Data, Standard ICD Code set^d^	19 (2.8%)	3 (1.0%)	16 (4.1%)

^a^Gold standard assessment of ADEs was based on patient interview data, adjudicated by a clinical expert to assess causality based on the Naranjo criteria. ^b^Potential ADEs in electronic prescribing data were defined as study drug discontinuations or dose changes due to safety or effectiveness reasons. ^c^Potential ADEs in administrative health data using the expanded ICD code set were defined as a physician visit, ED visit, or hospital admission during follow-up for which the recorded ICD code was (1) a relevant external cause code or (2) an adverse effect of the study drug. ^d^Potential ADEs in administrative health data using the standard ICD code set were defined as a physician visit, ED visit, or hospital admission during follow-up for which the recorded ICD code was (1) a relevant external cause code or (2) an adverse effect of the study drug *and* (3) was included in previously validated code sets.

Using our expanded, therapeutic class-targeted ICD code set applied to administrative health data, 62 (9.0%) patients experienced a potential ADE (51 (13.2%) patients on antihypertensives and 11 (3.6%) patients on antidepressants). Using the standard code set, 19 (2.8%) patients experienced a potential ADE (16 (4.1%) patients on antihypertensives and 3 (1.0%) patients on antidepressants). In patients who had a potential ADE based on our expanded code set, the most common recorded diagnoses were hyperglycemia (*N* = 21, 35.6%) and dyspnea (*N* = 10, 17.0%) among antihypertensive users and insomnia (*N* = 4, 36.4%) among antidepressant users (Additional file 2: Appendix 6).

Using MOXXI e-prescribing data, 40 (5.8%) patients experienced a potential ADE (20 (5.2%) patients on antihypertensives and 20 (6.6%) patients on antidepressants). Overall, 95% of potential ADEs were identified by drug discontinuations (90% antihypertensives, 100% antidepressants), and 5% by dose changes (10% antihypertensives, 0% antidepressants) (Additional file 2: Appendix 7). Drug discontinuation and dose changes identified as potential ADEs were more frequently made for safety (70%) than effectiveness reasons (30%). On average, treatment changes occurred within 30 days (SD 11 days) of the drug being dispensed.

### Sensitivity, specificity, PPV, and NPV

The sensitivity of our expanded, therapeutic class-targeted ICD code set was 7.0% (95% CI 3.1–13.4%) and the specificity was 90.6% (95% CI 87.9–92.9%) (Table 3). Treatment discontinuation or dose change orders in e-prescribing data had a sensitivity of 9.7% (95% CI 4.9–16.6%) and specificity of 95.0% (95% CI 92.8–96.6%) in detecting ADEs. For the standard code set, the sensitivity was 0%. Supplementing our expanded ICD code set with e-prescribing data doubled the sensitivity to 14.0% (95% CI 8.2–21.8%) and decreased specificity to 86.0% (95% CI 83.0–88.8%). The PPVs of the expanded ICD code set, e-prescribing data, and the two combined for ADE detection were 12.9% (95% CI 5.7–23.9%), 27.5% (95% CI 14.6–43.9%), and 16.7% (95% CI 9.8–25.7%), respectively. The NPVs of these approaches were 83.1% (95% CI 79.9–85.9%), 84.1% (95% CI 81.1–86.8%), and 83.5% (95% CI 80.2–86.4%), respectively.

**Table 3:** Accuracy of Electronic Prescribing Data and Diagnostic Codes in Administrative Health Data in Detecting Adverse Drug Events, Overall and by Medication Class

Data Source	Sensitivity (95% CI)	Specificity (95% CI)	PPV (95% CI)	NPV (95% CI)
**All Patients**				
Electronic Prescribing Data	9.7% (4.9–16.6%)	95.0% (92.8–96.6%)	27.5% (14.6–43.9%)	84.1% (81.1–86.8%)
Adminsitrative Health Data^a^	7.0% (3.1–13.4%)	90.6% (87.9–92.9%)	12.9% (5.7–23.9%)	83.1% (79.9–85.9%)
Electronic Prescribing & Administrative Health Data^a^	14.0% (8.2–21.8%)	86.1% (83.0–88.8%)	16.7% (9.8–25.7%)	83.5% (80.2–86.4%)
**Patients Prescribed an Antidepressant**				
Electronic Prescribing Data	13.5% (6.7–23.5%)	95.6% (92.1–97.9%)	50.0% (27.2–72.8%)	77.3% (72.0–82.1%)
Adminsitrative Health Data^a^	5.4% (1.5–13.3%)	94.7% (93.8–98.8%)	36.4% (10.9–69.2%)	76.0% (71.0–80.9%)
Electronic Prescribing & Administrative Health Data^a^	16.2% (8.7–26.6%)	92.5% (88.3–95.6%)	41.4% (23.5–61.1%)	77.3% (71.9–82.1%)
**Patients Prescribed an Antihypertensive**				
Electronic Prescribing Data	2.5% (0.0–13.2%)	94.5% (91.6–96.7%)	5.0% (0.1–24.9%)	89.3% (85.7–92.3%)
Adminsitrative Health Data^a^	10.0% (2.8–23.7%)	86.4% (82.3–89.9%)	7.8% (2.2–18.9%)	89.3% (85.4–92.4%)
Electronic Prescribing & Administrative Health Data^a^	10.0% (2.8–23.7%)	81.8% (77.3–85.7%)	6.0% (1.7–14.6%)	88.7% (84.7–92.0%)

*Abbreviations*: *CI* Confidence interval, *PPV* positive predictive value, *NPV* negative predictive value

^a^Results are presented for the expanded ICD code set

Assessing the accuracy of these approaches by therapeutic class revealed that while the sensitivity of e-prescribing data was higher among antidepressant users compared with antihypertensive users (13.5% vs 2.5%, respectively), the reverse was true regarding the sensitivity of our expanded ICD code set (5.4% vs 10.0%, respectively) (Table 3).

### Symptoms reported to physicians

Interview data indicate that 73% of study patients who reported new or worsening symptoms and health problems during their interview did not inform their doctor of these issues. Compared to patients who reported all their symptoms to their physician, patients who did not report symptoms experienced less severe symptoms (27.0% of symptoms were rated severe or very severe vs 51.9% of symptoms), were younger (mean age 61.5 vs 66.3), and were more likely to be male (29.6% vs 25.3%) (Additional file 2: Appendices 8–9). We also found that the sensitivity of our combined approach was improved almost two-fold when the analysis was restricted to patients who had reported symptoms to their physician (25.8% vs 14.0%).

## Discussion

In this validation study, we found that treatment change orders in e-prescribing data and diagnostic codes in administrative health data, individually and combined, enhanced the identification of ADEs caused by antidepressant and antihypertensive medications prescribed in a primary care setting compared to the standard code set. The higher sensitivity of our expanded ICD code set compared to the standard code set suggests that including diagnostic codes for known adverse effects of therapeutic drug classes improves ADE detection. However, the sensitivity of the expanded code set was still low, as even a comprehensive diagnostic code set will fail to capture ADEs if ICD codes are not reported in administrative health data. To fill this gap, we supplemented our expanded code set with e-prescribing data on change or discontinuation orders for safety or effectiveness reasons. Although this doubled the sensitivity, the sensitivity of our combined approach did not surpass 14.0%.

Our results can be explained, at least in part, by low rates of symptom reporting by patients to physicians. Indeed, 73% of study patients indicated in their interviews that they had not reported any of their symptoms to a physician. This suggests that e-prescribing and administrative health data alone may not detect mild ADEs for which patients do not seek medical attention. Even when patients do seek medical attention, there is discordance between the symptoms patients report and those documented in the electronic health record [56], which suggests that physician-reported data may not provide an accurate picture of patients’ symptoms. This could explain why, even when restricting analysis to patients who had reported symptoms to their physicians, the sensitivity of our combined approached remained relatively low. Thus, a potential alternative for ADE detection may lie in patient-reported outcomes (PROs), which have emerged as a necessary and increasingly promoted means of obtaining information from patients regarding their health status, including adverse events [57]. PROs have potential as a feasible and effective approach for ADE detection [58, 59], particularly if integrated into patient-centered digital health tools such as patient portals, mobile health (mHealth) applications, or even online reporting sites [60].

Interestingly, our subgroup analyses indicated that the sensitivity of treatment change orders in e-prescribing data was higher among antidepressant users compared with antihypertensive users. One potential explanation is that patients presenting to primary care physicians with an ADE related to new antidepressant use may be more likely to have their medication discontinued or changed. In contrast, patients with ADEs related to antihypertensive use may be prescribed an additional medication that targets the ADE, creating a medication cascade [61]. This is supported by a closer look at diagnostic codes of potential ADEs in administrative health data, the most common of which among antihypertensive users were for hyperglycemia. Physicians faced with a hyperglycemic patient may be more likely to prescribe a hypoglycemic medication than discontinue the antihypertensive [62]. This could explain the lower sensitivity of e-prescribing data among antihypertensive users.

Although our expanded, therapeutic class-targeted ICD code set was more sensitive than the standard code set in detecting ADEs in our cohort, its accuracy was similar to previously reported estimates for narrower code sets. In a 2001 study conducted in an outpatient setting, Honigman reported a PPV of 2% for the use of ICD-9 codes in identifying ADEs [23]. Similarly, Field found that computer-generated signals that included ICD-9 codes flagged true ADEs 7% of the time [27]. Results of both studies suggested low sensitivities of diagnostic codes in detecting ADEs, as did an investigation by Hohl in 2013 of the sensitivities of two ICD-10 code sets in detecting ADEs in the emergency department (6.8 and 28.1%) [20]. Despite using codes similar to those in our narrow standard code set, the sensitivities reported in these studies are more similar to that of our expanded code set. This may be explained by the data sources from which ICD codes were obtained in these studies, which differ from those used in our investigation. Electronic medical record data were used by the Field and Honigman studies, whereas Hohl used ED data from the National Ambulatory Care Reporting System (NACRS). The latter assigns ICD codes to ED discharge diagnoses by mapping selections made from a shortlist of 800 common term diagnoses to ICD-10-CA codes. Compared to the billing data used in our study, this process likely assigns ICD codes that more accurately reflect patients’ diagnoses. This could explain the higher sensitivity reported in Hohl despite their use of a narrower code set. In addition, as part of their process to identify gold standard ADEs, Hohl interviewed treating emergency physicians using a standardized questionnaire to determine patients’ working diagnoses. This may have made it more likely that treating physicians recognized and documented ADEs in the ED visit note, which in turn is used to assign ICD codes in NACRS, thus potentially overestimating the sensitivity of their code set.

Our study has several limitations. First, gold standard ADEs were often adjudicated based on incomplete information since lab test results and information on whether symptoms improved after medication discontinuation were often unavailable. These missing data impact on the Naranjo score calculation since item responses are recorded as “don’t know”. This uncertainty with missing information is a well-documented issue with the Naranjo algorithm, which nevertheless remains a frequently used method for ADE adjudication [63]. However, the ADE rates produced by our gold standard are similar to previously reported rates of adverse events attributable to antidepressant and antihypertensive medications [64–66], suggesting that the Naranjo algorithm produced accurate estimates in our sample. Second, the 3-week recall period used in patient interviews may have led to an underestimation of true ADE rates as patients may not have recalled mild symptoms that occurred earlier in this period. However, we expect such bias to be minimal as adverse effects of medications typically occur days or even weeks following the start of medication use [67, 68]. The 3-week window we used is meant to allow time for adverse effects to occur prior to the interview while minimizing poor recall. It is similar to (and in some cases even shorter than) recall periods used in previously published studies [45, 69–71]. In addition, the mean age of the study population was 64.2 years, of whom a low proportion would be expected to have significant cognitive problems impacting recall, although this was not specifically addressed. Third, administrative health data from the RAMQ record only the first 4 digits of ICD-9 codes, whereas many of the codes we identified as potential adverse effects of study drugs had up to 5 digits (and thus had to be truncated when applied to our data). This could have led to false positive ADEs detected using this approach. However, given the high specificity of administrative health data in detecting ADEs, this did not seem to be an issue in our particular sample. Finally, the generalizability of our results is unknown, as the accuracy of our ICD code set in detecting ADEs is largely dependent on how consistently and accurately ICD codes are recorded in billing data (since there are no systematic checks for data quality), which may vary between jurisdictions.

## Conclusion

The results of this validation study suggest that supplementing diagnostic codes in administrative health data with treatment change orders in electronic prescribing data doubles sensitivity. However, even when combined, these approaches do not possess sufficient accuracy to detect most ADEs resulting from medications prescribed in the community. Further research should be conducted to investigate the utility of patient-reported outcomes in detecting potential ADEs via emerging digital health technologies such as patient portals and mobile health applications.

## Supplementary Information

**Additional file 1. Patient Questionnaire**

**Additional file 2: Appendix 1**. Documented adverse effects of antidepressant and antihypertensive medications. **Appendix 2**. Expanded, Therapeutic Class-Targeted ICD Code Set. **Appendix 3**. Standard ICD Code Set. **Appendix 4**. Characteristics of consented patients, patients who declined consent, patients who were not dispensed the study drug, patients who were dispensed the study drug, and patients who were included in the final analysis. **Appendix 5**. Symptoms reported during interview, overall and by study drug therapeutic class, and among patients who reported all, none, or some of their symptoms to their physician. **Appendix 6**. Potential medication-related adverse effects experienced by antidepressant and antihypertensive users based on expanded, therapeutic class-targeted codeset. **Appendix 7**. Details of treatment changes among patients with a potential ADE based on e-prescribing data. **Appendix 8**. Characteristics of patients who reported all, none, or some of the symptoms they indicated in the interview to their physician. **Appendix 9**. Severity of symptoms experienced, as reported during interviews, by patients who reported all, none, or some of their symptoms to their physician.

## Data Availability

The datasets generated and analyzed during the current study are not publicly available due to privacy reasons, nor is a de-identified dataset available upon request.

## Abbreviations

ADE: Adverse Drug Event
ED: Emergency Department
ICD: Intenational Classification of Disease
MOXXI: Medical Office of the XXIst Century
RAMQ: Régie de l’assurance maladie du Québec
PPV: Positive Predictive Value
NPV: Negative Predictive Value
CI: Confidence Interval
SD: Standard deviation
SSRI: Selective serotonin reuptake inhibitor
SNRI: Serotonin-norepinephrine reuptake inhibitors
SRI: Serotonin reuptake inhibitor
NACRS: National Ambulatory Care Reporting System
