# Supplementary material for: Detection of adverse drug events in e-prescribing and administrative health data: a validation study
Source: BMC Health Serv Res. 2021 Apr 23;21:376. doi: 10.1186/s12913-021-06346-y (PMC8063436; doi:10.1186/s12913-021-06346-y)
Supplement: Supplementary file 1 — Additional file 1. Patient Questionnaire [file 12913_2021_6346_MOESM1_ESM.pdf]

Patient questionnaire ID………………

PART 1 : GENERAL INFORMATION ABOUT THE PATIENT, MEDICATION AND ANY NEW EVENT.

1: Sex: 1 □ Male 2 □ Female

2. Age: __________years old

3. How many times a day are/were you taking_________?

1 □ Once a day 4 □ Four times a day
2 □ Twice a day 5 □ Other (please indicate)
3 □ Three times a day

4. How many tablets or capsules are/were you taking each time?
1 □ One 4 □ Four
2 □ Two 5 □ Other (please indicate)
3 □ Three

5. When did you start taking your ___________? (__ __ / __ __ /__ __) date/month / year

6. What condition did your doctor prescribe this medicine for?_____________________.

7. Please list all other medicines that you have taken regularly **during the last 6 months**.

For each medicine please tick whether you started taking it before or after _________.

Please also tick whether you have now stopped taking any of these medicines.

**Name of Medicine**	**Started ?**	**Stopped ?**
__________________________	□ Before □ After	□ Yes □ No
__________________________	□ Before □ After	□ Yes □ No
__________________________	□ Before □ After	□ Yes □ No
__________________________	□ Before □ After	□ Yes □ No
__________________________	□ Before □ After	□ Yes □ No

8. Do you have any other medical conditions? 1 □ Yes 2 □ No
If yes, please list them____.

9. Since you started taking____________, do you develop any event including new symptoms, change in your condition, abnormal changes in laboratory tests, failure of therapeutic effect, accidents, falls etc

10. Have you been in hospital or emergency department for any reason after starting the drug?
1 □ Yes 2 □ No
If yes, what was the reason?

PART 2 (standardized system-related probes to elicit problems that may have been overlooked, and **drug-specific standardized probes** to elicit negative or confirmatory evidence on known possible drug class-specific effects).

After starting the drug, have you had any of the following symptoms
Only indicate the problems which were not present before you started taking ________________

1. Have you had any of the following symptoms related to your **skin**?

1 ☐ bleeding skin
2 ☐ bruising
3 ☐ burning
4 ☐ **flushing of skin/ hot flush**
5 ☐ **increased sensitivity of skin to light**
6 ☐ **itching of skin**
7 ☐ pale
8 ☐ **puffy skin sensation**
9 ☐ **pins and needles sensation**
10 ☐ **skin rash**
11 ☐ **yellowing of skin**
12 ☐ Other (please indicate)
13 ☐ None

2. Have you had any of the following symptoms related to your **hair or nails**?

1 ☐ change in fingernails
2 ☐ hair loss
3 ☐ Other (please indicate)
4 ☐ None

3. Have you had any of the following symptoms related to your **muscle, bones or joints**?

1 ☐ bone or joint pain
2 ☐ **muscle pain**
3 ☐ muscle weakness
4 ☐ trembling & shaking of fingers & hands
5 ☐ unsteadiness on feet
6 ☐ unusual or uncontrolled body movement
7 ☐ Other (please indicate)
8 ☐ None

4. Have you had any of the following symptoms related to **your head**?

1 ❑ **headache**
2 ❑ migraine headache
3 ❑ others
4 ❑ None

5. Have you had any of the following symptoms related to **your vision**?

1 ☐ blurred vision
2 ☐ double vision
3 ☐ Other (please indicate)
4 ☐ None

6. Have you had any of the following symptoms related to **your eyes**?

1 ☐ itchy or irritated or inflamed eyes or eyelids
2 ☐ inability to move eyes
3 ☐ unusual movement of the eyes
4 ☐ Other (please indicate)
5 ☐ None

7. Have you had any of the following symptoms related to **hearing or ears**?

1 ☐ change or difficulty in hearing
2 ☐ feeling of fullness in the ears
3 ☐ ringing, buzzing or noises in ears
4 ☐ Other (please indicate)
5 ☐ None

8. Have you had any of the following symptoms related to **mouth or gums**?

1 ☐ bleeding from gums
2 ☐ dry mouth or throat
3 ☐ Other (please indicate)
4 ☐ None

9. Have you had any of the following symptoms related to your **nose, throat, neck or voice**?

1 ☐ difficulty talking
2 ☐ slurred speech
3 ☐ runny or stuffy nose
4 ☐ sore throat

5 ☐ Other (please indicate)
6 ☐ None

10. Have you had any of the following symptoms related to your **breathing or lungs**?
1 ☐ **cough**
2 ☐ **difficulty breathing**
3 ☐ fast breathing
4☐ slow breathing
5 ☐ Other (please indicate)
6 ☐ None

11. Have you had any of the following symptoms related to your **heart or circulation**?
1 ☐ **palpitations/ racing heart**
2 ☐ missed heart beat
3 ❑ **shortness of breath**
4 ❑ **chest pain**
5 ❑ **swelling of feet**
6 ☐ Other (please indicate)
7 ☐ None

12. Have you had any of the following symptoms related to your **stomach or digestive system**?
1 ☐ bloated feeling or gas
2 ☐ decrease in appetite
3 ☐ indigestion or heartburn
4 ☐ increase in appetite
5 ☐ pain or cramps in lower abdomen
6 ☐ nausea or vomiting
7 ☐ vomiting blood or material that looks like coffee grounds
8 ☐ Other (please indicate)
9 ☐ None

13. Have you had any of the following symptoms related to your **rectum or bowel movements**?
1 ☐ **black tarry stool**
2 ☐ constipation
3 ☐ **diarrhoea**
4 ☐ Other (please indicate)
5 ☐ None

14. Have you had any of the following symptoms related to your **kidneys, bladder or urinary system**?
1 ☐ burning, discomfort or pain while passing water
2 ☐ dark brown urine
3 ☐ **difficulty in passing water**
4 ☐ **passing water less often**
5 ☐ **passing water more often**
6 ☐ bloody urine
7 ☐ Other (please indicate)
8 ☐ None

15. Have you had any of the following symptoms related to your **sexual function (ability)**?
1 ☐ **decrease in sexual desire**
2 ☐ **decrease in sexual ability**
3 ☐ increase in sexual desire
4 ☐ Other (please indicate)
5 ☐ None
6 ☐ Does not apply

16. Have you had any of the following symptoms related to your **reproductive (sex) organ**?
1 ☐ abnormal or change in vaginal bleeding
2 ☐ burning or irritated penis
3 ☐ Other (please indicate)
4 ☐ None

17. Have you had any of the following symptoms to your **nervous system**?
1 ☐ confusion or delirium
2 ☐ **light-headed when getting up from a lying or sitting position or feeling faint**
3 ☐ **dizziness or staggering** (vertigo)
4 ☐ **increase in convulsions (seizures)**
5 ☐ Other (please indicate)
6 ❑ None

18. Have you had any of the following symptoms related to your **mental health**?
1 ☐ anxiety (nervousness) or agitation
2 ☐ change in mood
3 ☐ difficulty concentrating or learning
4 ☐ **hallucinations (seeing, hearing or feeling things that are not there)**
5 ☐ nightmares
6 ☐ anger or aggression
7 ☐ loss of memory
8 ☐ **thought of suicide**
9 ☐ reduction in sleeping
10 ☐ increase sleep or drowsiness
11 ☐ Other (please indicate)

12 □ None

19. Have you had any of the following **symptoms or told to have change in laboratory values**?

1 □ increased sensitivity to cold
2 □ **excessive thirst**
3 □ fever
4 □ flu-like symptoms
5 □ increase sweating
6 □ **unusual tiredness or weakness**
7 □ **weight gain**
8 □ **increased liver enzymes**
9 ❑ **increased glucose level**
10 ❑ **decreased potassium level**
11 ❑ **decreased sodium level**
12 □ Other (please indicate)
13 □ None

20. Which, if any, of the symptoms in question 1 - 19 have bothered you most?

21.i. a How much has this symptom in question 20 bothered you at its worst?

1 □ minimally
2 □ mildly
3 □ moderately
4 □ severely
5 □ very severely
6 □ does not apply

21.i.b. How long did this symptom last?

1 □ less than a day
2 □ less than a week
3 □ one to two weeks
4 □ more than two weeks

21.ii.a. How much has the other symptom(s) bothered you at its worst?

1 □ minimally
2 □ mildly
3 □ moderately
4 □ severely
5 □ very severely
6 □ does not apply

21.ii.b. How long did the other symptoms last?

1 □ less than a day
2 □ less than a week
3 □ one to two weeks
4 □ more than two weeks

22. Have you told your doctor about any of these symptom(s)?

1 □ all
2 □ some
3 □ none
4 □ I'm not sure
5 □ does not apply

23. Have you stopped taking the drug in question?

1 □ No 2 □ Yes , please go to Part 3

24. Have you experienced any of the above symptoms with this drug previously (in the past) ?

1 ❑ No 2 ❑ Yes

If yes, month/year (__ __ / __ __)

PART 3 (Questions related to stopped drugs and rechallenges)

1. When did you stop this medicine? (__ __ / __ __ / __ __) date/month / year

2. Why did you stop?
1 I felt I didn't need it any longer
2 The doctor said I didn't need it any longer
3 The doctor told me to stop because I was having problems with it
4 I decided to stop because I was having problems with it
5 I felt it wasn't helping me
6 Other (please explain)

3. Have any of the symptoms you have described gone away?
1 Yes 2 No 3 Does not apply
If yes, please say which ones.

4. Have any other symptoms started after stopping______________?
1 Yes 2 No
If yes, please describe them here.

5. Have you re-started the drug?
1 ❑ Yes 2 ❑ No
If yes, please go to next question.

6. Have you experienced any of the symptoms in questions from 1 – 19 (Part 2) after the initiation of the drug?
1 ❑ Yes 2 ❑ No
If yes, please list them.

Part 4 (Questions on address and education).

1. Address, email,

2. What is the highest level of school you have completed?

a. some high school
b. graduated from high school
c. graduated from vocational, trade, or technical school
d. some college
e. college graduate
