# Supplementary material for: Detection of adverse drug events in e-prescribing and administrative health data: a validation study
Source: BMC Health Serv Res. 2021 Apr 23;21:376. doi: 10.1186/s12913-021-06346-y (PMC8063436; doi:10.1186/s12913-021-06346-y)
Supplement: Supplementary file 2 — Additional file 2: Appendix 1. Documented adverse effects of antidepressant and antihypertensive medications. Appendix 2. Expanded, Therapeutic Class-Targeted ICD Code Set. Appendix 3. Standard ICD Code Set. Appendix 4. Characteristics of consented patients, patients who declined consent, patients who were not dispensed the study drug, patients who were dispensed the study drug, and patients who were included in the final analysis. Appendix 5. Symptoms reported during interview, overall and by study drug therapeutic class, and among patients who reported all, none, or some of their symptoms to their physician. Appendix 6. Potential medication-related adverse effects experienced by antidepressant and antihypertensive users based on expanded, therapeutic class-targeted codeset. Appendix 7. Details of treatment changes among patients with a potential ADE based on e-prescribing data. Appendix 8. Characteristics of patients who reported all, none, or some of the symptoms they indicated in the interview to their physician. Appendix 9. Severity of symptoms experienced, as reported during interviews, by patients who reported all, none, or some of their symptoms to their physician. [file 12913_2021_6346_MOESM2_ESM.docx]

# Appendix 1. Documented adverse effects of antidepressant and antihypertensive medications

| Adverse Effect | Medication Class |
| --- | --- |
| AE by antidepressant | antidepressant |
| diarrhea | antidepressant |
| dizziness | antidepressant |
| dry mouth | antidepressant |
| fall | antidepressant |
| fatigue | antidepressant |
| fracture | antidepressant |
| headache | antidepressant |
| insomnia | antidepressant |
| ischemic stroke | antidepressant |
| nausea/vomiting | antidepressant |
| poisoning by antidepressant | antidepressant |
| sexual dysfunction | antidepressant |
| suicidality/self-harm | antidepressant |
| sweating | antidepressant |
| tremor | antidepressant |
| upper GI tract bleeding | antidepressant |
| weight gain | antidepressant |
| AE by antihypertensive | antihypertensive |
| angioedema | antihypertensive |
| bradycardia | antihypertensive |
| constipation | antihypertensive |
| cough | antihypertensive |
| dizziness/vertigo | antihypertensive |
| dyspnea | antihypertensive |
| fatigue | antihypertensive |
| flushing | antihypertensive |
| headache | antihypertensive |
| hepatotoxicity | antihypertensive |
| hypovolemia | antihypertensive |
| hyperglycemia | antihypertensive |
| hyperkalemia | antihypertensive |
| hyperlipidemia | antihypertensive |
| hypokalemia | antihypertensive |
| hypomagnesemia | antihypertensive |
| hyponatremia | antihypertensive |
| hypotension | antihypertensive |
| metabolic alkalosis | antihypertensive |
| nausea | antihypertensive |
| neutropenia | antihypertensive |
| peripheral edema | antihypertensive |
| photosensitivity dermatitis | antihypertensive |
| poisoning by antihypertensive | antihypertensive |
| psoriasis | antihypertensive |
| rash | antihypertensive |
| renal impairment | antihypertensive |
| sexual dysfunction | antihypertensive |
| taste disturbances | antihypertensive |
| weight gain | antihypertensive |

**Appendix 2. Expanded, Therapeutic Class-Targeted ICD Code Set**

| Adverse Effect | Code Type | Code | Code Description | Medication Class |
| --- | --- | --- | --- | --- |
| AE by antidep | icd10 | Y490 | Tricyclic and tetracyclic antidepressants causing adverse effect in therapeutic use | antidepressant |
| AE by antidep | icd10 | Y491 | Monoamine-oxidase-inhibitor antidepressants causing adverse effect in therapeutic use | antidepressant |
| AE by antidep | icd10 | Y492 | Other and unspecified antidepressants causing adverse effect in therapeutic use | antidepressant |
| diarrhea | icd10 | K528 | Other specified noninfective gastroenteritis and colitis | antidepressant |
| diarrhea | icd10 | K529 | Noninfective gastroenteritis and colitis, unspecified | antidepressant |
| diarrhea | icd10 | R194 | Change in bowel habit | antidepressant |
| diarrhea | icd10 | R198 | Other specified symptoms and signs involving the digestive system and abdomen | antidepressant |
| dizziness | icd10 | H814 | Vertigo of central origin | antidepressant |
| dizziness | icd10 | R42 | Dizziness and giddiness | antidepressant |
| dry mouth | icd10 | K117 | Disturbances of salivary secretion | antidepressant |
| dry mouth | icd10 | R682 | Dry mouth, unspecified | antidepressant |
| fall | icd10 | W00 | Fall on same level involving ice and snow | antidepressant |
| fall | icd10 | W00-W1 | Falls (W00-W19) | antidepressant |
| fall | icd10 | W01 | Fall on same level from slipping, tripping and stumbling | antidepressant |
| fall | icd10 | W02 | Fall involving skates, skis, sport boards and in-line skates | antidepressant |
| fall | icd10 | W0200 | Fall involving ice skates | antidepressant |
| fall | icd10 | W0201 | Fall involving skis | antidepressant |
| fall | icd10 | W0202 | Fall involving roller skates/in-line skates | antidepressant |
| fall | icd10 | W0203 | Fall involving skateboards | antidepressant |
| fall | icd10 | W0204 | Fall involving snowboards | antidepressant |
| fall | icd10 | W0208 | Fall other specified | antidepressant |
| fall | icd10 | W03 | Other fall on same level due to collision with, or pushing by, another person | antidepressant |
| fall | icd10 | W04 | Fall while being carried or supported by other persons | antidepressant |
| fall | icd10 | W05 | Fall involving wheelchair and other types of walking devices | antidepressant |
| fall | icd10 | W0500 | Fall involving wheelchair | antidepressant |
| fall | icd10 | W0501 | Fall involving adult walker | antidepressant |
| fall | icd10 | W0502 | Fall involving baby walker | antidepressant |
| fall | icd10 | W0503 | Fall involving stroller/carriage | antidepressant |
| fall | icd10 | W0504 | Chute d' un chariot d' épicerie | antidepressant |
| fall | icd10 | W0508 | Fall involving other specified walking devices | antidepressant |
| fall | icd10 | W0509 | Fall involving unspecified walking devices | antidepressant |
| fall | icd10 | W06 | Fall involving bed | antidepressant |
| fall | icd10 | W07 | Fall involving chair | antidepressant |
| fall | icd10 | W08 | Fall involving other furniture | antidepressant |
| fall | icd10 | W09 | Fall involving playground equipment | antidepressant |
| fall | icd10 | W0901 | Chute impliquant une balançoire | antidepressant |
| fall | icd10 | W0902 | Chute impliquant une glissoire, un toboggan | antidepressant |
| fall | icd10 | W0903 | Chute impliquant une balançoire à bascule | antidepressant |
| fall | icd10 | W0904 | Chute impliquant une cage à grimper | antidepressant |
| fall | icd10 | W0905 | Chute impliquant une trampoline | antidepressant |
| fall | icd10 | W0908 | Chute impliquant autre agrès équipant un terrain de jeux | antidepressant |
| fall | icd10 | W0909 | Chute impliquant agrès équipant un terrain de jeux, sans précision | antidepressant |
| fall | icd10 | W10 | Fall on and from stairs and steps | antidepressant |
| fall | icd10 | W11 | Fall on and from ladder | antidepressant |
| fall | icd10 | W12 | Fall on and from scaffolding | antidepressant |
| fall | icd10 | W13 | Fall from, out of or through building or structure | antidepressant |
| fall | icd10 | W14 | Fall from tree | antidepressant |
| fall | icd10 | W15 | Fall from cliff | antidepressant |
| fall | icd10 | W16 | Diving or jumping into water causing injury other than drowning or submersion | antidepressant |
| fall | icd10 | W17 | Other fall from one level to another | antidepressant |
| fall | icd10 | W18 | Other fall on same level | antidepressant |
| fall | icd10 | W19 | Unspecified fall | antidepressant |
| fall | icd10 | X590 | Exposure to unspecified factor causing fracture | antidepressant |
| fatigue | icd10 | R400 | Somnolence | antidepressant |
| fatigue | icd10 | R53 | Malaise and fatigue | antidepressant |
| fracture | icd10 | M484 | Fatigue fracture of vertebra | antidepressant |
| fracture | icd10 | M4840 | Fatigue fracture of vertebra, multiple sites in spine | antidepressant |
| fracture | icd10 | M4841 | Fatigue fracture of vertebra, occipito-atlanto-axial region | antidepressant |
| fracture | icd10 | M4842 | Fatigue fracture of vertebra, cervical region | antidepressant |
| fracture | icd10 | M4843 | Fatigue fracture of vertebra, cervicothoracic region | antidepressant |
| fracture | icd10 | M4844 | Fatigue fracture of vertebra, thoracic region | antidepressant |
| fracture | icd10 | M4845 | Fatigue fracture of vertebra, thoracolumbar region | antidepressant |
| fracture | icd10 | M4846 | Fatigue fracture of vertebra, lumbar region | antidepressant |
| fracture | icd10 | M4847 | Fatigue fracture of vertebra, lumbosacral region | antidepressant |
| fracture | icd10 | M4848 | Fatigue fracture of vertebra, sacral and sacrococcygeal region | antidepressant |
| fracture | icd10 | M4849 | Fatigue fracture of vertebra, unspecified site | antidepressant |
| fracture | icd10 | M840 | Malunion of fracture | antidepressant |
| fracture | icd10 | M8400 | Malunion of fracture, multiple sites | antidepressant |
| fracture | icd10 | M8401 | Malunion of fracture, shoulder region | antidepressant |
| fracture | icd10 | M8402 | Malunion of fracture, upper arm | antidepressant |
| fracture | icd10 | M8403 | Malunion of fracture, forearm | antidepressant |
| fracture | icd10 | M8404 | Malunion of fracture, hand | antidepressant |
| fracture | icd10 | M8405 | Malunion of fracture, pelvic region and thigh | antidepressant |
| fracture | icd10 | M8406 | Malunion of fracture, lower leg | antidepressant |
| fracture | icd10 | M8407 | Malunion of fracture, ankle and foot | antidepressant |
| fracture | icd10 | M8408 | Malunion of fracture, other site | antidepressant |
| fracture | icd10 | M8409 | Malunion of fracture, unspecified site | antidepressant |
| fracture | icd10 | M841 | Nonunion of fracture [pseudarthrosis] | antidepressant |
| fracture | icd10 | M8410 | Nonunion of fracture [pseudarthrosis], multiple sites | antidepressant |
| fracture | icd10 | M8411 | Nonunion of fracture [pseudarthrosis], shoulder region | antidepressant |
| fracture | icd10 | M8412 | Nonunion of fracture [pseudarthrosis], upper arm | antidepressant |
| fracture | icd10 | M8413 | Nonunion of fracture [pseudarthrosis], forearm | antidepressant |
| fracture | icd10 | M8414 | Nonunion of fracture [pseudarthrosis], hand | antidepressant |
| fracture | icd10 | M8415 | Nonunion of fracture [pseudarthrosis], pelvic region and thigh | antidepressant |
| fracture | icd10 | M8416 | Nonunion of fracture [pseudarthrosis], lower leg | antidepressant |
| fracture | icd10 | M8417 | Nonunion of fracture [pseudarthrosis], ankle and foot | antidepressant |
| fracture | icd10 | M8418 | Nonunion of fracture [pseudarthrosis], other site | antidepressant |
| fracture | icd10 | M8419 | Nonunion of fracture [pseudarthrosis], unspecified site | antidepressant |
| fracture | icd10 | M842 | Delayed union of fracture | antidepressant |
| fracture | icd10 | M8420 | Delayed union of fracture, multiple sites | antidepressant |
| fracture | icd10 | M8421 | Delayed union of fracture, shoulder region | antidepressant |
| fracture | icd10 | M8422 | Delayed union of fracture, upper arm | antidepressant |
| fracture | icd10 | M8423 | Delayed union of fracture, forearm | antidepressant |
| fracture | icd10 | M8424 | Delayed union of fracture, hand | antidepressant |
| fracture | icd10 | M8425 | Delayed union of fracture, pelvic region and thigh | antidepressant |
| fracture | icd10 | M8426 | Delayed union of fracture, lower leg | antidepressant |
| fracture | icd10 | M8427 | Delayed union of fracture, ankle and foot | antidepressant |
| fracture | icd10 | M8428 | Delayed union of fracture, other site | antidepressant |
| fracture | icd10 | M8429 | Delayed union of fracture, unspecified site | antidepressant |
| fracture | icd10 | M843 | Stress fracture, not elsewhere classified | antidepressant |
| fracture | icd10 | M8430 | Stress fracture, not elsewhere classified, multiple sites | antidepressant |
| fracture | icd10 | M8431 | Stress fracture, not elsewhere classified, shoulder region | antidepressant |
| fracture | icd10 | M8432 | Stress fracture, not elsewhere classified, upper arm | antidepressant |
| fracture | icd10 | M8433 | Stress fracture, not elsewhere classified, forearm | antidepressant |
| fracture | icd10 | M8434 | Stress fracture, not elsewhere classified, hand | antidepressant |
| fracture | icd10 | M8435 | Stress fracture, not elsewhere classified, pelvic region and thigh | antidepressant |
| fracture | icd10 | M8436 | Stress fracture, not elsewhere classified, lower leg | antidepressant |
| fracture | icd10 | M8437 | Stress fracture, not elsewhere classified, ankle and foot | antidepressant |
| fracture | icd10 | M8438 | Stress fracture, not elsewhere classified, other site | antidepressant |
| fracture | icd10 | M8439 | Stress fracture, not elsewhere classified, unspecified site | antidepressant |
| fracture | icd10 | M844 | Pathological fracture, not elsewhere classified | antidepressant |
| fracture | icd10 | M8440 | Pathological fracture, not elsewhere classified, multiple sites | antidepressant |
| fracture | icd10 | M8441 | Pathological fracture, not elsewhere classified, shoulder region | antidepressant |
| fracture | icd10 | M8442 | Pathological fracture, not elsewhere classified, upper arm | antidepressant |
| fracture | icd10 | M8443 | Pathological fracture, not elsewhere classified, forearm | antidepressant |
| fracture | icd10 | M8444 | Pathological fracture, not elsewhere classified, hand | antidepressant |
| fracture | icd10 | M8445 | Pathological fracture, not elsewhere classified, pelvic region and thigh | antidepressant |
| fracture | icd10 | M8446 | Pathological fracture, not elsewhere classified, lower leg | antidepressant |
| fracture | icd10 | M8447 | Pathological fracture, not elsewhere classified, ankle and foot | antidepressant |
| fracture | icd10 | M8448 | Pathological fracture, not elsewhere classified, other site | antidepressant |
| fracture | icd10 | M8449 | Pathological fracture, not elsewhere classified, unspecified site | antidepressant |
| fracture | icd10 | S02 | Fracture of skull and facial bones | antidepressant |
| fracture | icd10 | S020 | Fracture of vault of skull | antidepressant |
| fracture | icd10 | S02000 | Fracture of vault of skull, closed | antidepressant |
| fracture | icd10 | S02001 | Fracture of vault of skull, open | antidepressant |
| fracture | icd10 | S021 | Fracture of base of skull | antidepressant |
| fracture | icd10 | S02100 | Fracture of base of skull, closed | antidepressant |
| fracture | icd10 | S02101 | Fracture of base of skull, open | antidepressant |
| fracture | icd10 | S022 | Fracture of nasal bones | antidepressant |
| fracture | icd10 | S02200 | Fracture of nasal bones, closed | antidepressant |
| fracture | icd10 | S02201 | Fracture of nasal bones, open | antidepressant |
| fracture | icd10 | S023 | Fracture of orbital floor | antidepressant |
| fracture | icd10 | S02300 | Fracture of orbital floor, closed | antidepressant |
| fracture | icd10 | S02301 | Fracture of orbital floor, open | antidepressant |
| fracture | icd10 | S024 | Fracture of malar and maxillary bones | antidepressant |
| fracture | icd10 | S0240 | Fracture of malar and maxillary bones, LeFort 1 | antidepressant |
| fracture | icd10 | S02400 | Fracture of malar and maxillary bones, LeFort 1, closed | antidepressant |
| fracture | icd10 | S02401 | Fracture of malar and maxillary bones, LeFort 1, open | antidepressant |
| fracture | icd10 | S0241 | Fracture of malar and maxillary bones, LeFort 2 | antidepressant |
| fracture | icd10 | S02410 | Fracture of malar and maxillary bones, LeFort 2, closed | antidepressant |
| fracture | icd10 | S02411 | Fracture of malar and maxillary bones, LeFort 2, open | antidepressant |
| fracture | icd10 | S0242 | Fracture of malar and maxillary bones, combined midface | antidepressant |
| fracture | icd10 | S02420 | Fracture of malar and maxillary bones, combined midface, closed | antidepressant |
| fracture | icd10 | S02421 | Fracture of malar and maxillary bones, combined midface, open | antidepressant |
| fracture | icd10 | S0243 | Fracture of malar and maxillary bones, LeFort 3, unilateral | antidepressant |
| fracture | icd10 | S02430 | Fracture of malar and maxillary bones, LeFort 3, unilateral, closed | antidepressant |
| fracture | icd10 | S02431 | Fracture of malar and maxillary bones, LeFort 3, unilateral, open | antidepressant |
| fracture | icd10 | S0244 | Fracture of malar and maxillary bones, bilateral LeFort 3 | antidepressant |
| fracture | icd10 | S02440 | Fracture of malar and maxillary bones, bilateral LeFort 3, closed | antidepressant |
| fracture | icd10 | S02441 | Fracture of malar and maxillary bones, bilateral LeFort 3, open | antidepressant |
| fracture | icd10 | S0248 | Other fracture of malar and maxillary bones | antidepressant |
| fracture | icd10 | S02480 | Other fracture of malar and maxillary bones, closed | antidepressant |
| fracture | icd10 | S02481 | Other fracture of malar and maxillary bones, open | antidepressant |
| fracture | icd10 | S0249 | Unspecified fracture of malar and maxillary bones | antidepressant |
| fracture | icd10 | S02490 | Unspecified fracture of malar and maxillary bones, closed | antidepressant |
| fracture | icd10 | S02491 | Unspecified fracture of malar and maxillary bones, open | antidepressant |
| fracture | icd10 | S025 | Fracture of tooth | antidepressant |
| fracture | icd10 | S026 | Fracture of mandible | antidepressant |
| fracture | icd10 | S0260 | Fracture of mandible | antidepressant |
| fracture | icd10 | S02600 | Fracture of mandible, closed | antidepressant |
| fracture | icd10 | S02601 | Fracture of mandible, open | antidepressant |
| fracture | icd10 | S0261 | Fracture of ramus | antidepressant |
| fracture | icd10 | S02610 | Fracture of ramus, closed | antidepressant |
| fracture | icd10 | S02611 | Fracture of ramus, open | antidepressant |
| fracture | icd10 | S0262 | Fracture of temporomandibular joint | antidepressant |
| fracture | icd10 | S02620 | Fracture of temporomandibular joint, closed | antidepressant |
| fracture | icd10 | S02621 | Fracture of temporomandibular joint, open | antidepressant |
| fracture | icd10 | S0267 | Multiple mandibular fracture sites | antidepressant |
| fracture | icd10 | S02670 | Multiple mandibular fracture sites, closed | antidepressant |
| fracture | icd10 | S02671 | Multiple mandibular fracture sites, open | antidepressant |
| fracture | icd10 | S027 | Multiple fractures involving skull and facial bone | antidepressant |
| fracture | icd10 | S0270 | Multiple fractures involving skull and facial bone | antidepressant |
| fracture | icd10 | S02700 | Multiple fractures involving skull and facial bones, closed | antidepressant |
| fracture | icd10 | S02701 | Multiple fractures involving skull and facial bones, open | antidepressant |
| fracture | icd10 | S028 | Fractures of other skull and facial bones | antidepressant |
| fracture | icd10 | S0280 | Fracture of alveolus | antidepressant |
| fracture | icd10 | S02800 | Fracture of alveolus, closed | antidepressant |
| fracture | icd10 | S02801 | Fracture of alveolus, open | antidepressant |
| fracture | icd10 | S0281 | Fracture of hard palate | antidepressant |
| fracture | icd10 | S02810 | Fracture of hard palate, closed | antidepressant |
| fracture | icd10 | S02811 | Fracture of hard palate, open | antidepressant |
| fracture | icd10 | S0289 | Fractures of other unspecified skull and facial bones | antidepressant |
| fracture | icd10 | S02890 | Fracture of other and unspecified skull and facial bones NEC, closed | antidepressant |
| fracture | icd10 | S02891 | Fracture of other and unspecified skull and facial bones NEC, open | antidepressant |
| fracture | icd10 | S029 | Fracture of skull and facial bones, part unspecified | antidepressant |
| fracture | icd10 | S0290 | Fracture of skull and facial bones, part unspecified | antidepressant |
| fracture | icd10 | S02900 | Fracture of skull and facial bones, part unspecified, closed | antidepressant |
| fracture | icd10 | S02901 | Fracture of skull and facial bones, part unspecified, open | antidepressant |
| fracture | icd10 | S071 | Crushing injury of skull | antidepressant |
| fracture | icd10 | S078 | Crushing injury of other parts of head | antidepressant |
| fracture | icd10 | S079 | Crushing injury of head, part unspecified | antidepressant |
| fracture | icd10 | S12 | Fracture of neck | antidepressant |
| fracture | icd10 | S120 | Fracture of first cervical vertebra | antidepressant |
| fracture | icd10 | S12000 | Fracture of first cervical vertebra, closed | antidepressant |
| fracture | icd10 | S12001 | Fracture of first cervical vertebra, open | antidepressant |
| fracture | icd10 | S121 | Fracture of second cervical vertebra | antidepressant |
| fracture | icd10 | S12100 | Fracture of second cervical vertebra, closed | antidepressant |
| fracture | icd10 | S12101 | Fracture of second cervical vertebra, open | antidepressant |
| fracture | icd10 | S122 | Fracture of other specified cervical vertebra | antidepressant |
| fracture | icd10 | S1220 | Fracture of C3 - C4 vertebra | antidepressant |
| fracture | icd10 | S12200 | Fracture of C3 - C4 vertebra, closed | antidepressant |
| fracture | icd10 | S12201 | Fracture of C3 - C4 vertebra, open | antidepressant |
| fracture | icd10 | S1221 | Fracture of C5 - C7 vertebra | antidepressant |
| fracture | icd10 | S12210 | Fracture of C5 - C7 vertebra, closed | antidepressant |
| fracture | icd10 | S12211 | Fracture of C5 - C7 vertebra, open | antidepressant |
| fracture | icd10 | S127 | Multiple fractures of cervical spine | antidepressant |
| fracture | icd10 | S12700 | Multiple fractures of cervical spine, closed | antidepressant |
| fracture | icd10 | S12701 | Multiple fractures of cervical spine, open | antidepressant |
| fracture | icd10 | S128 | Fracture of other parts of neck | antidepressant |
| fracture | icd10 | S12800 | Fracture of other parts of neck, closed | antidepressant |
| fracture | icd10 | S12801 | Fracture of other parts of neck, open | antidepressant |
| fracture | icd10 | S129 | Fracture of neck, part unspecified | antidepressant |
| fracture | icd10 | S12900 | Fracture of neck, part unspecified, closed | antidepressant |
| fracture | icd10 | S12901 | Fracture of neck, part unspecified, open | antidepressant |
| fracture | icd10 | S22 | Fracture of rib(s), sternum and thoracic spine | antidepressant |
| fracture | icd10 | S220 | Fracture of thoracic vertebra | antidepressant |
| fracture | icd10 | S2200 | Fracture of thoracic vertebra T1 - T6 | antidepressant |
| fracture | icd10 | S22000 | Fracture of thoracic vertebra T1 - T6, closed | antidepressant |
| fracture | icd10 | S22001 | Fracture of thoracic vertebra T1 - T6, open | antidepressant |
| fracture | icd10 | S2201 | Fracture of thoracic vertebra T7- T12 | antidepressant |
| fracture | icd10 | S22010 | Fracture of thoracic vertebra T7- T12, closed | antidepressant |
| fracture | icd10 | S22011 | Fracture of thoracic vertebra T7- T12, open | antidepressant |
| fracture | icd10 | S2209 | Fracture of unspecified thoracic vertebra | antidepressant |
| fracture | icd10 | S22090 | Fracture of unspecified thoracic vertebra, closed | antidepressant |
| fracture | icd10 | S22091 | Fracture of unspecified thoracic vertebra, open | antidepressant |
| fracture | icd10 | S221 | Multiple fractures of thoracic spine | antidepressant |
| fracture | icd10 | S22100 | Multiple fractures of thoracic spine, closed | antidepressant |
| fracture | icd10 | S22101 | Multiple fractures of thoracic spine, open | antidepressant |
| fracture | icd10 | S222 | Fracture of sternum | antidepressant |
| fracture | icd10 | S22200 | Fracture of sternum, closed | antidepressant |
| fracture | icd10 | S22201 | Fracture of sternum, open | antidepressant |
| fracture | icd10 | S223 | Fracture of rib | antidepressant |
| fracture | icd10 | S22300 | Fracture of rib, closed | antidepressant |
| fracture | icd10 | S22301 | Fracture of rib, open | antidepressant |
| fracture | icd10 | S224 | Multiple fractures of ribs | antidepressant |
| fracture | icd10 | S2240 | Multiple fractures of 2 - 4 ribs | antidepressant |
| fracture | icd10 | S22400 | Multiple fractures of 2 - 4 ribs, closed | antidepressant |
| fracture | icd10 | S22401 | Multiple fractures of 2 - 4 ribs, open | antidepressant |
| fracture | icd10 | S2241 | Multiple fractures of 5 or more ribs | antidepressant |
| fracture | icd10 | S22410 | Multiple fractures of 5 or more ribs, closed | antidepressant |
| fracture | icd10 | S22411 | Multiple fractures of 5 or more ribs, open | antidepressant |
| fracture | icd10 | S2249 | Multiple fractures of unspecified number of ribs | antidepressant |
| fracture | icd10 | S22490 | Multiple fractures of unspecified number of ribs, closed | antidepressant |
| fracture | icd10 | S22491 | Multiple fractures of unspecified number of ribs, open | antidepressant |
| fracture | icd10 | S22500 | Flail chest, closed | antidepressant |
| fracture | icd10 | S22501 | Flail chest, open | antidepressant |
| fracture | icd10 | S228 | Fracture of other parts of bony thorax | antidepressant |
| fracture | icd10 | S22800 | Fracture of other parts of bony thorax, closed | antidepressant |
| fracture | icd10 | S22801 | Fracture of other parts of bony thorax, open | antidepressant |
| fracture | icd10 | S229 | Fracture of bony thorax, part unspecified | antidepressant |
| fracture | icd10 | S22900 | Fracture of bony thorax, part unspecified, closed | antidepressant |
| fracture | icd10 | S22901 | Fracture of bony thorax, part unspecified, open | antidepressant |
| fracture | icd10 | S32 | Fracture of lumbar spine and pelvis | antidepressant |
| fracture | icd10 | S320 | Fracture of lumbar vertebra | antidepressant |
| fracture | icd10 | S3200 | Fracture of lumbar vertebra, L1 level | antidepressant |
| fracture | icd10 | S32000 | Fracture of lumbar vertebra, L1 level, closed | antidepressant |
| fracture | icd10 | S32001 | Fracture of lumbar vertebra, L1 level, open | antidepressant |
| fracture | icd10 | S3201 | Fracture of lumbar vertebra, L2 level | antidepressant |
| fracture | icd10 | S32010 | Fracture of lumbar vertebra, L2 level, closed | antidepressant |
| fracture | icd10 | S32011 | Fracture of lumbar vertebra, L2 level, open | antidepressant |
| fracture | icd10 | S3202 | Fracture of lumbar vertebra, L3 level | antidepressant |
| fracture | icd10 | S32020 | Fracture of lumbar vertebra, L3 level, closed | antidepressant |
| fracture | icd10 | S32021 | Fracture of lumbar vertebra, L3 level, open | antidepressant |
| fracture | icd10 | S3203 | Fracture of lumbar vertebra, L4 level | antidepressant |
| fracture | icd10 | S32030 | Fracture of lumbar vertebra, L4 level, closed | antidepressant |
| fracture | icd10 | S32031 | Fracture of lumbar vertebra, L4 level, open | antidepressant |
| fracture | icd10 | S3204 | Fracture of lumbar vertebra, L5 level | antidepressant |
| fracture | icd10 | S32040 | Fracture of lumbar vertebra, L5 level, closed | antidepressant |
| fracture | icd10 | S32041 | Fracture of lumbar vertebra, L5 level, open | antidepressant |
| fracture | icd10 | S3209 | Fracture of lumbar vertebra, unspecified level | antidepressant |
| fracture | icd10 | S32090 | Fracture of lumbar vertebra, unspecified level, closed | antidepressant |
| fracture | icd10 | S32091 | Fracture of lumbar vertebra, unspecified level, open | antidepressant |
| fracture | icd10 | S321 | Fracture of sacrum | antidepressant |
| fracture | icd10 | S32100 | Fracture of sacrum, closed | antidepressant |
| fracture | icd10 | S32101 | Fracture of sacrum, open | antidepressant |
| fracture | icd10 | S322 | Fracture of coccyx | antidepressant |
| fracture | icd10 | S32200 | Fracture of coccyx, closed | antidepressant |
| fracture | icd10 | S32201 | Fracture of coccyx, open | antidepressant |
| fracture | icd10 | S323 | Fracture of ilium | antidepressant |
| fracture | icd10 | S32300 | Fracture of ilium, closed | antidepressant |
| fracture | icd10 | S32301 | Fracture of ilium, open | antidepressant |
| fracture | icd10 | S324 | Fracture of acetabulum | antidepressant |
| fracture | icd10 | S32400 | Fracture of acetabulum, closed | antidepressant |
| fracture | icd10 | S32401 | Fracture of acetabulum, open | antidepressant |
| fracture | icd10 | S325 | Fracture of pubis | antidepressant |
| fracture | icd10 | S32500 | Fracture of pubis, closed | antidepressant |
| fracture | icd10 | S32501 | Fracture of pubis, open | antidepressant |
| fracture | icd10 | S327 | Multiple fractures of lumbar spine and pelvis | antidepressant |
| fracture | icd10 | S32700 | Multiple fractures of lumbar spine and pelvis, closed | antidepressant |
| fracture | icd10 | S32701 | Multiple fractures of lumbar spine and pelvis, open | antidepressant |
| fracture | icd10 | S328 | Fracture of other and unspecified parts of lumbar spine and pelvis | antidepressant |
| fracture | icd10 | S32800 | Fracture of other and unspecified parts of lumbar spine and pelvis, closed | antidepressant |
| fracture | icd10 | S32801 | Fracture of other and unspecified parts of lumbar spine and pelvis, open | antidepressant |
| fracture | icd10 | S42 | Fracture of shoulder and upper arm | antidepressant |
| fracture | icd10 | S420 | Fracture of clavicle | antidepressant |
| fracture | icd10 | S4200 | Fracture of sternal end of clavicle | antidepressant |
| fracture | icd10 | S42000 | Fracture of sternal end of clavicle, closed | antidepressant |
| fracture | icd10 | S42001 | Fracture of sternal end of clavicle, open | antidepressant |
| fracture | icd10 | S4201 | Fracture of shaft of clavicle | antidepressant |
| fracture | icd10 | S42010 | Fracture of shaft of clavicle, closed | antidepressant |
| fracture | icd10 | S42011 | Fracture of shaft of clavicle, open | antidepressant |
| fracture | icd10 | S4202 | Fracture of acromial end of clavicle | antidepressant |
| fracture | icd10 | S42020 | Fracture of acromial end of clavicle, closed | antidepressant |
| fracture | icd10 | S42021 | Fracture of acromial end of clavicle, open | antidepressant |
| fracture | icd10 | S4209 | Fracture of unspecified part of clavicle | antidepressant |
| fracture | icd10 | S42090 | Fracture of unspecified part of clavicle, closed | antidepressant |
| fracture | icd10 | S42091 | Fracture of unspecified part of clavicle, open | antidepressant |
| fracture | icd10 | S421 | Fracture of scapula | antidepressant |
| fracture | icd10 | S4210 | Fracture of acromial process of scapula | antidepressant |
| fracture | icd10 | S42100 | Fracture of acromial process of scapula, closed | antidepressant |
| fracture | icd10 | S42101 | Fracture of acromial process of scapula, open | antidepressant |
| fracture | icd10 | S4211 | Fracture of coracoid process of scapula | antidepressant |
| fracture | icd10 | S42110 | Fracture of coracoid process of scapula, closed | antidepressant |
| fracture | icd10 | S42111 | Fracture of coracoid process of scapula, open | antidepressant |
| fracture | icd10 | S4212 | Fracture of glenoid cavity and neck of scapula | antidepressant |
| fracture | icd10 | S42120 | Fracture of glenoid cavity and neck of scapula, closed | antidepressant |
| fracture | icd10 | S42121 | Fracture of glenoid cavity and neck of scapula, open | antidepressant |
| fracture | icd10 | S4218 | Fracture of other part of scapula | antidepressant |
| fracture | icd10 | S42180 | Fracture of other part of scapula, closed | antidepressant |
| fracture | icd10 | S42181 | Fracture of other part of scapula, open | antidepressant |
| fracture | icd10 | S4219 | Fracture of unspecified part of scapula | antidepressant |
| fracture | icd10 | S42190 | Fracture of unspecified part of scapula, closed | antidepressant |
| fracture | icd10 | S42191 | Fracture of unspecified part of scapula, open | antidepressant |
| fracture | icd10 | S422 | Fracture of upper end of humerus | antidepressant |
| fracture | icd10 | S4220 | Fracture of surgical neck of humerus | antidepressant |
| fracture | icd10 | S42200 | Fracture of surgical neck of humerus, closed | antidepressant |
| fracture | icd10 | S42201 | Fracture of surgical neck of humerus, open | antidepressant |
| fracture | icd10 | S4221 | Fracture of anatomical neck of humerus | antidepressant |
| fracture | icd10 | S42210 | Fracture of anatomical neck of humerus, closed | antidepressant |
| fracture | icd10 | S42211 | Fracture of anatomical neck of humerus, open | antidepressant |
| fracture | icd10 | S4222 | Fracture of greater tuberosity of humerus | antidepressant |
| fracture | icd10 | S42220 | Fracture of greater tuberosity of humerus, closed | antidepressant |
| fracture | icd10 | S42221 | Fracture of greater tuberosity of humerus, open | antidepressant |
| fracture | icd10 | S4228 | Fracture of other part of upper end of humerus | antidepressant |
| fracture | icd10 | S42280 | Fracture of other part of upper end of humerus, closed | antidepressant |
| fracture | icd10 | S42281 | Fracture of other part of upper end of humerus, open | antidepressant |
| fracture | icd10 | S4229 | Fracture of unspecified part of upper end of humerus | antidepressant |
| fracture | icd10 | S42290 | Fracture of unspecified part of upper end of humerus, closed | antidepressant |
| fracture | icd10 | S42291 | Fracture of unspecified part of upper end of humerus, open | antidepressant |
| fracture | icd10 | S423 | Fracture of shaft of humerus | antidepressant |
| fracture | icd10 | S4230 | Fracture of shaft of humerus NOS | antidepressant |
| fracture | icd10 | S42300 | Fracture of shaft of humerus, closed | antidepressant |
| fracture | icd10 | S42301 | Fracture of shaft of humerus, open | antidepressant |
| fracture | icd10 | S4239 | Fracture of unspecified part of humerus | antidepressant |
| fracture | icd10 | S42390 | Fracture of unspecified part of humerus, closed | antidepressant |
| fracture | icd10 | S42391 | Fracture of unspecified part of humerus, open | antidepressant |
| fracture | icd10 | S424 | Fracture of lower end of humerus | antidepressant |
| fracture | icd10 | S4240 | Supracondylar fracture of humerus | antidepressant |
| fracture | icd10 | S42400 | Supracondylar fracture of humerus, closed | antidepressant |
| fracture | icd10 | S42401 | Supracondylar fracture of humerus, open | antidepressant |
| fracture | icd10 | S4241 | Fracture of lateral condyle of humerus | antidepressant |
| fracture | icd10 | S42410 | Fracture of lateral condyle of humerus, closed | antidepressant |
| fracture | icd10 | S42411 | Fracture of lateral condyle of humerus, open | antidepressant |
| fracture | icd10 | S4242 | Fracture of medial condyle of humerus | antidepressant |
| fracture | icd10 | S42420 | Fracture of medial condyle of humerus, closed | antidepressant |
| fracture | icd10 | S42421 | Fracture of medial condyle of humerus, open | antidepressant |
| fracture | icd10 | S4243 | Fracture of unspecified condyle of humerus | antidepressant |
| fracture | icd10 | S42430 | Fracture of unspecified condyle of humerus, closed | antidepressant |
| fracture | icd10 | S42431 | Fracture of unspecified condyle of humerus, open | antidepressant |
| fracture | icd10 | S4248 | Fracture of other part of lower end of humerus | antidepressant |
| fracture | icd10 | S42480 | Fracture of other part of lower end of humerus, closed | antidepressant |
| fracture | icd10 | S42481 | Fracture of other part of lower end of humerus, open | antidepressant |
| fracture | icd10 | S4249 | Fracture of unspecified part of lower part of humerus | antidepressant |
| fracture | icd10 | S42490 | Fracture of unspecified part of lower part of humerus, closed | antidepressant |
| fracture | icd10 | S42491 | Fracture of unspecified part of lower part of humerus, open | antidepressant |
| fracture | icd10 | S427 | Multiple fractures of clavicle, scapula and humerus | antidepressant |
| fracture | icd10 | S42700 | Multiple fractures of clavicle, scapula and humerus, closed | antidepressant |
| fracture | icd10 | S42701 | Multiple fractures of clavicle, scapula and humerus, open | antidepressant |
| fracture | icd10 | S428 | Fracture of other parts of shoulder and upper arm | antidepressant |
| fracture | icd10 | S42800 | Fracture of other parts of shoulder and upper arm, closed | antidepressant |
| fracture | icd10 | S42801 | Fracture of other parts of shoulder and upper arm, open | antidepressant |
| fracture | icd10 | S429 | Fracture of shoulder girdle, part unspecified | antidepressant |
| fracture | icd10 | S42900 | Fracture of shoulder girdle, part unspecified, closed | antidepressant |
| fracture | icd10 | S42901 | Fracture of shoulder girdle, part unspecified, open | antidepressant |
| fracture | icd10 | S52 | Fracture of forearm | antidepressant |
| fracture | icd10 | S520 | Fracture of upper end of ulna | antidepressant |
| fracture | icd10 | S5200 | Fracture of olecranon process of ulna | antidepressant |
| fracture | icd10 | S52000 | Fracture of olecranon process of ulna, closed | antidepressant |
| fracture | icd10 | S52001 | Fracture of olecranon process of ulna, open | antidepressant |
| fracture | icd10 | S5201 | Fracture of coronoid process of ulna | antidepressant |
| fracture | icd10 | S52010 | Fracture of coronoid process of ulna, closed | antidepressant |
| fracture | icd10 | S52011 | Fracture of coronoid process of ulna, open | antidepressant |
| fracture | icd10 | S5202 | Monteggia's fracture | antidepressant |
| fracture | icd10 | S52020 | Monteggia's fracture, closed | antidepressant |
| fracture | icd10 | S52021 | Monteggia's fracture, open | antidepressant |
| fracture | icd10 | S5208 | Other/multiple fractures of upper end of ulna | antidepressant |
| fracture | icd10 | S52080 | Other/multiple fractures of upper end of ulna, closed | antidepressant |
| fracture | icd10 | S52081 | Other/multiple fractures of upper end of ulna, open | antidepressant |
| fracture | icd10 | S5209 | Unspecified fracture of upper end of ulna | antidepressant |
| fracture | icd10 | S52090 | Unspecified fracture of upper end of ulna, closed | antidepressant |
| fracture | icd10 | S52091 | Unspecified fracture of upper end of ulna, open | antidepressant |
| fracture | icd10 | S521 | Fracture of upper end of radius | antidepressant |
| fracture | icd10 | S5210 | Fracture of head of radius | antidepressant |
| fracture | icd10 | S52100 | Fracture of head of radius, closed | antidepressant |
| fracture | icd10 | S52101 | Fracture of head of radius, open | antidepressant |
| fracture | icd10 | S5211 | Fracture of neck of radius | antidepressant |
| fracture | icd10 | S52110 | Fracture of neck of radius, closed | antidepressant |
| fracture | icd10 | S52111 | Fracture of neck of radius, open | antidepressant |
| fracture | icd10 | S5212 | Fracture of radius with ulna, upper end | antidepressant |
| fracture | icd10 | S52120 | Fracture of radius with ulna, upper end, closed | antidepressant |
| fracture | icd10 | S52121 | Fracture of radius with ulna, upper end, open | antidepressant |
| fracture | icd10 | S5218 | Other/multiple fracture of upper end of radius | antidepressant |
| fracture | icd10 | S52180 | Other/multiple fracture of upper end of radius, closed | antidepressant |
| fracture | icd10 | S52181 | Other/multiple fracture of upper end of radius, open | antidepressant |
| fracture | icd10 | S5219 | Unspecified fracture of upper end of radius | antidepressant |
| fracture | icd10 | S52190 | Unspecified fracture of upper end of radius, closed | antidepressant |
| fracture | icd10 | S52191 | Unspecified fracture of upper end of radius, open | antidepressant |
| fracture | icd10 | S522 | Fracture of shaft of ulna | antidepressant |
| fracture | icd10 | S52200 | Fracture of shaft of ulna, closed | antidepressant |
| fracture | icd10 | S52201 | Fracture of shaft of ulna, open | antidepressant |
| fracture | icd10 | S523 | Fracture of shaft of radius | antidepressant |
| fracture | icd10 | S52300 | Fracture of shaft of radius, closed | antidepressant |
| fracture | icd10 | S52301 | Fracture of shaft of radius, open | antidepressant |
| fracture | icd10 | S524 | Fracture of shafts of both ulna and radius | antidepressant |
| fracture | icd10 | S52400 | Fracture of shafts of both ulna and radius, closed | antidepressant |
| fracture | icd10 | S52401 | Fracture of shafts of both ulna and radius, open | antidepressant |
| fracture | icd10 | S525 | Fracture of lower end of radius | antidepressant |
| fracture | icd10 | S5250 | Colles' fracture | antidepressant |
| fracture | icd10 | S52500 | Colles' fracture, closed | antidepressant |
| fracture | icd10 | S52501 | Colles' fracture, open | antidepressant |
| fracture | icd10 | S5258 | Other fracture of lower end of radius | antidepressant |
| fracture | icd10 | S52580 | Other fracture of lower end of radius, closed | antidepressant |
| fracture | icd10 | S52581 | Other fracture of lower end of radius, open | antidepressant |
| fracture | icd10 | S5259 | Unspecified fracture of lower end of radius | antidepressant |
| fracture | icd10 | S52590 | Unspecified fracture of lower end of radius, closed | antidepressant |
| fracture | icd10 | S52591 | Unspecified fracture of lower end of radius, open | antidepressant |
| fracture | icd10 | S526 | Fracture of lower end of both ulna and radius | antidepressant |
| fracture | icd10 | S52600 | Fracture of lower end of both ulna and radius, closed | antidepressant |
| fracture | icd10 | S52601 | Fracture of lower end of both ulna and radius, open | antidepressant |
| fracture | icd10 | S527 | Multiple fractures of forearm | antidepressant |
| fracture | icd10 | S52700 | Multiple fractures of forearm, closed | antidepressant |
| fracture | icd10 | S52701 | Multiple fractures of forearm, open | antidepressant |
| fracture | icd10 | S528 | Fracture of other parts of forearm | antidepressant |
| fracture | icd10 | S52800 | Fracture of other parts of forearm, closed | antidepressant |
| fracture | icd10 | S52801 | Fracture of other parts of forearm, open | antidepressant |
| fracture | icd10 | S529 | Fracture of forearm, part unspecified | antidepressant |
| fracture | icd10 | S52900 | Fracture of forearm, part unspecified, closed | antidepressant |
| fracture | icd10 | S52901 | Fracture of forearm, part unspecified, open | antidepressant |
| fracture | icd10 | S62 | Fracture at wrist and hand level | antidepressant |
| fracture | icd10 | S620 | Fracture of navicular [scaphoid] bone of hand | antidepressant |
| fracture | icd10 | S62000 | Fracture of navicular [scaphoid] bone of hand, closed | antidepressant |
| fracture | icd10 | S62001 | Fracture of navicular [scaphoid] bone of hand, open | antidepressant |
| fracture | icd10 | S621 | Fracture of other carpal bone(s) | antidepressant |
| fracture | icd10 | S6210 | Fracture of lunate bone | antidepressant |
| fracture | icd10 | S62100 | Fracture of lunate bone, closed | antidepressant |
| fracture | icd10 | S62101 | Fracture of lunate bone, open | antidepressant |
| fracture | icd10 | S6211 | Fracture of triquetral bone | antidepressant |
| fracture | icd10 | S62110 | Fracture of triquetral bone, closed | antidepressant |
| fracture | icd10 | S62111 | Fracture of triquetral bone, open | antidepressant |
| fracture | icd10 | S6212 | Fracture of pisiform bone | antidepressant |
| fracture | icd10 | S62120 | Fracture of pisiform bone, closed | antidepressant |
| fracture | icd10 | S62121 | Fracture of pisiform bone, open | antidepressant |
| fracture | icd10 | S6213 | Fracture of trapezium bone | antidepressant |
| fracture | icd10 | S62130 | Fracture of trapezium bone, closed | antidepressant |
| fracture | icd10 | S62131 | Fracture of trapezium bone, open | antidepressant |
| fracture | icd10 | S6214 | Fracture of trapezoid bone | antidepressant |
| fracture | icd10 | S62140 | Fracture of trapezoid bone, closed | antidepressant |
| fracture | icd10 | S62141 | Fracture of trapezoid bone, open | antidepressant |
| fracture | icd10 | S6215 | Fracture of capitate bone | antidepressant |
| fracture | icd10 | S62150 | Fracture of capitate bone, closed | antidepressant |
| fracture | icd10 | S62151 | Fracture of capitate bone, open | antidepressant |
| fracture | icd10 | S6216 | Fracture of hamate bone | antidepressant |
| fracture | icd10 | S62160 | Fracture of hamate bone, closed | antidepressant |
| fracture | icd10 | S62161 | Fracture of hamate bone, open | antidepressant |
| fracture | icd10 | S6218 | Fracture of other carpal bones | antidepressant |
| fracture | icd10 | S62180 | Fracture of other carpal bones, closed | antidepressant |
| fracture | icd10 | S62181 | Fracture of other carpal bones, open | antidepressant |
| fracture | icd10 | S6219 | Fracture of unspecified carpal bones | antidepressant |
| fracture | icd10 | S62190 | Fracture of unspecified carpal bones, closed | antidepressant |
| fracture | icd10 | S62191 | Fracture of unspecified carpal bones, open | antidepressant |
| fracture | icd10 | S622 | Fracture of first metacarpal bone | antidepressant |
| fracture | icd10 | S6220 | Fracture of base of first metacarpal bone | antidepressant |
| fracture | icd10 | S62200 | Fracture of base of first metacarpal bone, closed | antidepressant |
| fracture | icd10 | S62201 | Fracture of base of first metacarpal bone, open | antidepressant |
| fracture | icd10 | S6221 | Fracture of shaft of first metacarpal bone | antidepressant |
| fracture | icd10 | S62210 | Fracture of shaft of first metacarpal bone, closed | antidepressant |
| fracture | icd10 | S62211 | Fracture of shaft of first metacarpal bone, open | antidepressant |
| fracture | icd10 | S6222 | Fracture of neck of first metacarpal bone | antidepressant |
| fracture | icd10 | S62220 | Fracture of neck of first metacarpal bone, closed | antidepressant |
| fracture | icd10 | S62221 | Fracture of neck of first metacarpal bone, open | antidepressant |
| fracture | icd10 | S6227 | Fracture of multiple sites of first metacarpal bone | antidepressant |
| fracture | icd10 | S62270 | Fracture of multiple sites of first metacarpal bone, closed | antidepressant |
| fracture | icd10 | S62271 | Fracture of multiple sites of first metacarpal bone, open | antidepressant |
| fracture | icd10 | S6229 | Fracture of unspecified site of first metacarpal bone | antidepressant |
| fracture | icd10 | S62290 | Fracture of unspecified site of first metacarpal bone, closed | antidepressant |
| fracture | icd10 | S62291 | Fracture of unspecified site of first metacarpal bone, open | antidepressant |
| fracture | icd10 | S623 | Fracture of other metacarpal bone | antidepressant |
| fracture | icd10 | S6230 | Fracture of base of other metacarpal bone | antidepressant |
| fracture | icd10 | S62300 | Fracture of base of other metacarpal bone, closed | antidepressant |
| fracture | icd10 | S62301 | Fracture of base of other metacarpal bone, open | antidepressant |
| fracture | icd10 | S6231 | Fracture of shaft of other metacarpal bone | antidepressant |
| fracture | icd10 | S62310 | Fracture of shaft of other metacarpal bone, closed | antidepressant |
| fracture | icd10 | S62311 | Fracture of shaft of other metacarpal bone, open | antidepressant |
| fracture | icd10 | S6232 | Fracture of head of other metacarpal bone | antidepressant |
| fracture | icd10 | S62320 | Fracture of head of other metacarpal bone, closed | antidepressant |
| fracture | icd10 | S62321 | Fracture of head of other metacarpal bone, open | antidepressant |
| fracture | icd10 | S6237 | Fracture of multiple sites of other metacarpal bones | antidepressant |
| fracture | icd10 | S62370 | Fracture of multiple sites of other metacarpal bones, closed | antidepressant |
| fracture | icd10 | S62371 | Fracture of multiple sites of other metacarpal bones, open | antidepressant |
| fracture | icd10 | S6239 | Fracture of unspecified site of other metacarpal bone | antidepressant |
| fracture | icd10 | S62390 | Fracture of unspecified site of other metacarpal bone, closed | antidepressant |
| fracture | icd10 | S62391 | Fracture of unspecified site of other metacarpal bone, open | antidepressant |
| fracture | icd10 | S624 | Multiple fractures of metacarpal bones | antidepressant |
| fracture | icd10 | S62400 | Multiple fractures of metacarpal bones, closed | antidepressant |
| fracture | icd10 | S62401 | Multiple fractures of metacarpal bones, open | antidepressant |
| fracture | icd10 | S62410 | Fractures multiples de la diaphyse d'autres os du métacarpe, fermée | antidepressant |
| fracture | icd10 | S62411 | Fractures multiples de la diaphyse d'autres os du métacarpe, ouverte | antidepressant |
| fracture | icd10 | S62420 | Fractures multiples de la tête d'autres os du métacarpe, fermée | antidepressant |
| fracture | icd10 | S62421 | Fractures multiples de la tête d'autres os du métacarpe, ouverte | antidepressant |
| fracture | icd10 | S62470 | Fractures multiples de sites multiples d'autres os du métacarpe, fermée | antidepressant |
| fracture | icd10 | S62471 | Fractures multiples de sites multiples d'autres os du métacarpe, ouverte | antidepressant |
| fracture | icd10 | S62490 | Fractures multiples d'autres os du métacarpe sans précision, fermée | antidepressant |
| fracture | icd10 | S62491 | Fractures multiples d'autres os du métacarpe sans précision, ouverte | antidepressant |
| fracture | icd10 | S625 | Fracture of thumb | antidepressant |
| fracture | icd10 | S6250 | Fracture of proximal phalanx | antidepressant |
| fracture | icd10 | S62500 | Fracture of proximal phalanx, closed | antidepressant |
| fracture | icd10 | S62501 | Fracture of proximal phalanx, open | antidepressant |
| fracture | icd10 | S6251 | Fracture of distal phalanx | antidepressant |
| fracture | icd10 | S62510 | Fracture of distal phalanx, closed | antidepressant |
| fracture | icd10 | S62511 | Fracture of distal phalanx, open | antidepressant |
| fracture | icd10 | S6257 | Fracture of multiple sites of thumb | antidepressant |
| fracture | icd10 | S62570 | Fracture of multiple sites of thumb, closed | antidepressant |
| fracture | icd10 | S62571 | Fracture of multiple sites of thumb, open | antidepressant |
| fracture | icd10 | S6259 | Fracture of unspecified part of phalanx | antidepressant |
| fracture | icd10 | S62590 | Fracture of unspecified part of phalanx, closed | antidepressant |
| fracture | icd10 | S62591 | Fracture of unspecified part of phalanx, open | antidepressant |
| fracture | icd10 | S626 | Fracture of other finger | antidepressant |
| fracture | icd10 | S6260 | Fracture of middle or proximal phalanx of finger | antidepressant |
| fracture | icd10 | S62600 | Fracture of middle or proximal phalanx of finger, closed | antidepressant |
| fracture | icd10 | S62601 | Fracture of middle or proximal phalanx of finger, open | antidepressant |
| fracture | icd10 | S6261 | Fracture of distal phalanx of finger | antidepressant |
| fracture | icd10 | S62610 | Fracture of distal phalanx of finger, closed | antidepressant |
| fracture | icd10 | S62611 | Fracture of distal phalanx of finger, open | antidepressant |
| fracture | icd10 | S6267 | Fracture of multiple sites of finger | antidepressant |
| fracture | icd10 | S62670 | Fracture of multiple sites of finger, closed | antidepressant |
| fracture | icd10 | S62671 | Fracture of multiple sites of finger, open | antidepressant |
| fracture | icd10 | S6269 | Fracture of unspecified part of phalanx of finger | antidepressant |
| fracture | icd10 | S62690 | Fracture of unspecified part of phalanx of finger, closed | antidepressant |
| fracture | icd10 | S62691 | Fracture of unspecified part of phalanx of finger, open | antidepressant |
| fracture | icd10 | S627 | Multiple fractures of fingers | antidepressant |
| fracture | icd10 | S62700 | Multiple fractures of fingers, closed | antidepressant |
| fracture | icd10 | S62701 | Multiple fractures of fingers, open | antidepressant |
| fracture | icd10 | S628 | Fracture of other and unspecified parts of wrist and hand | antidepressant |
| fracture | icd10 | S62800 | Fracture of other and unspecified parts of wrist and hand, closed | antidepressant |
| fracture | icd10 | S62801 | Fracture of other and unspecified parts of wrist and hand, open | antidepressant |
| fracture | icd10 | S72 | Fracture of femur | antidepressant |
| fracture | icd10 | S720 | Fracture of neck of femur | antidepressant |
| fracture | icd10 | S7200 | Fracture of upper femoral epiphysis (separation) | antidepressant |
| fracture | icd10 | S72000 | Fracture of upper femoral epiphysis (separation), closed | antidepressant |
| fracture | icd10 | S72001 | Fracture of upper femoral epiphysis (separation) open | antidepressant |
| fracture | icd10 | S7201 | Fracture of base of femoral neck (cervicotrochanteric) | antidepressant |
| fracture | icd10 | S72010 | Fracture of base of femoral neck (cervicotrochanteric) closed | antidepressant |
| fracture | icd10 | S72011 | Fracture of base of femoral neck (cervicotrochanteric) open | antidepressant |
| fracture | icd10 | S7208 | Other fracture of femoral neck | antidepressant |
| fracture | icd10 | S72080 | Other fracture of femoral neck, closed | antidepressant |
| fracture | icd10 | S72081 | Other fracture of femoral neck, open | antidepressant |
| fracture | icd10 | S7209 | Unspecified fracture of neck of femur | antidepressant |
| fracture | icd10 | S72090 | Unspecified fracture of neck of femur, closed | antidepressant |
| fracture | icd10 | S72091 | Unspecified fracture of neck of femur, open | antidepressant |
| fracture | icd10 | S721 | Pertrochanteric fracture | antidepressant |
| fracture | icd10 | S7210 | Intertrochanteric fracture | antidepressant |
| fracture | icd10 | S72100 | Intertrochanteric fracture, closed | antidepressant |
| fracture | icd10 | S72101 | Intertrochanteric fracture, open | antidepressant |
| fracture | icd10 | S7219 | Unspecified trochanteric fracture | antidepressant |
| fracture | icd10 | S72190 | Unspecified trochanteric fracture, closed | antidepressant |
| fracture | icd10 | S72191 | Unspecified trochanteric fracture, open | antidepressant |
| fracture | icd10 | S722 | Subtrochanteric fracture | antidepressant |
| fracture | icd10 | S72200 | Subtrochanteric fracture, closed | antidepressant |
| fracture | icd10 | S72201 | Subtrochanteric fracture, open | antidepressant |
| fracture | icd10 | S723 | Fracture of shaft of femur | antidepressant |
| fracture | icd10 | S72300 | Fracture of shaft of femur, closed | antidepressant |
| fracture | icd10 | S72301 | Fracture of shaft of femur, open | antidepressant |
| fracture | icd10 | S724 | Fracture of lower end of femur | antidepressant |
| fracture | icd10 | S7240 | Fracture of lower femoral epiphysis (separation) | antidepressant |
| fracture | icd10 | S72400 | Fracture of lower femoral epiphysis (separation), closed | antidepressant |
| fracture | icd10 | S72401 | Fracture of lower femoral epiphysis (separation), open | antidepressant |
| fracture | icd10 | S7241 | Condylar fracture of femur | antidepressant |
| fracture | icd10 | S72410 | Condylar fracture of femur, closed | antidepressant |
| fracture | icd10 | S72411 | Condylar fracture of femur, open | antidepressant |
| fracture | icd10 | S7242 | Supracondylar fracture of femur | antidepressant |
| fracture | icd10 | S72420 | Supracondylar fracture of femur, closed | antidepressant |
| fracture | icd10 | S72421 | Supracondylar fracture of femur, open | antidepressant |
| fracture | icd10 | S7249 | Unspecified fracture of lower (distal) end of femur | antidepressant |
| fracture | icd10 | S72490 | Unspecified fracture of lower (distal) end of femur, closed | antidepressant |
| fracture | icd10 | S72491 | Unspecified fracture of lower (distal) end of femur, open | antidepressant |
| fracture | icd10 | S727 | Multiple fractures of femur | antidepressant |
| fracture | icd10 | S72700 | Multiple fractures of femur, closed | antidepressant |
| fracture | icd10 | S72701 | Multiple fractures of femur, open | antidepressant |
| fracture | icd10 | S728 | Fractures of other parts of femur | antidepressant |
| fracture | icd10 | S72800 | Fractures of other parts of femur, closed | antidepressant |
| fracture | icd10 | S72801 | Fractures of other parts of femur, open | antidepressant |
| fracture | icd10 | S729 | Fracture of femur, part unspecified | antidepressant |
| fracture | icd10 | S72900 | Fracture of femur, part unspecified, closed | antidepressant |
| fracture | icd10 | S72901 | Fracture of femur, part unspecified, open | antidepressant |
| fracture | icd10 | S82 | Fracture of lower leg, including ankle | antidepressant |
| fracture | icd10 | S820 | Fracture of patella | antidepressant |
| fracture | icd10 | S82000 | Fracture of patella, closed | antidepressant |
| fracture | icd10 | S82001 | Fracture of patella, open | antidepressant |
| fracture | icd10 | S821 | Fracture of upper end of tibia | antidepressant |
| fracture | icd10 | S82100 | Fracture of upper (proximal) end of tibia with or without fibula, closed | antidepressant |
| fracture | icd10 | S82101 | Fracture of upper (proximal) end of tibia with or without fibula, open | antidepressant |
| fracture | icd10 | S822 | Fracture of shaft of tibia | antidepressant |
| fracture | icd10 | S82200 | Fracture of shaft of tibia with or without fibula, closed | antidepressant |
| fracture | icd10 | S82201 | Fracture of shaft of tibia with or without fibula, open | antidepressant |
| fracture | icd10 | S823 | Fracture of lower end of tibia | antidepressant |
| fracture | icd10 | S82300 | Fracture of lower (distal) end of tibia with or without fibula, closed | antidepressant |
| fracture | icd10 | S82301 | Fracture of lower (distal) end of tibia with or without fibula, open | antidepressant |
| fracture | icd10 | S824 | Fracture of fibula alone | antidepressant |
| fracture | icd10 | S82400 | Fracture of fibula alone, closed | antidepressant |
| fracture | icd10 | S82401 | Fracture of fibula alone, open | antidepressant |
| fracture | icd10 | S825 | Fracture of medial malleolus | antidepressant |
| fracture | icd10 | S82500 | Fracture of medial malleolus, closed | antidepressant |
| fracture | icd10 | S82501 | Fracture of medial malleolus, open | antidepressant |
| fracture | icd10 | S826 | Fracture of lateral malleolus | antidepressant |
| fracture | icd10 | S82600 | Fracture of lateral malleolus, closed | antidepressant |
| fracture | icd10 | S82601 | Fracture of lateral malleolus, open | antidepressant |
| fracture | icd10 | S827 | Multiple fractures of lower leg | antidepressant |
| fracture | icd10 | S82700 | Multiple fractures of lower leg, closed | antidepressant |
| fracture | icd10 | S82701 | Multiple fractures of lower leg, open | antidepressant |
| fracture | icd10 | S828 | Fractures of other parts of lower leg | antidepressant |
| fracture | icd10 | S8280 | Bimalleolar fracture of ankle | antidepressant |
| fracture | icd10 | S82800 | Bimalleolar fracture of ankle, closed | antidepressant |
| fracture | icd10 | S82801 | Bimalleolar fracture of ankle, open | antidepressant |
| fracture | icd10 | S8281 | Trimalleolar fracture of ankle | antidepressant |
| fracture | icd10 | S82810 | Trimalleolar fracture of ankle, closed | antidepressant |
| fracture | icd10 | S82811 | Trimalleolar fracture of ankle, open | antidepressant |
| fracture | icd10 | S8282 | Tibial plafond fracture of ankle with or without fibular/talar involvement | antidepressant |
| fracture | icd10 | S82820 | Tibial plafond fracture of ankle with or without fibular/talar involvement closed | antidepressant |
| fracture | icd10 | S82821 | Tibial plafond fracture of ankle with or without fibular/tarsal involvement open | antidepressant |
| fracture | icd10 | S8289 | Fracture of ankle NOS | antidepressant |
| fracture | icd10 | S82890 | Fracture of ankle NOS, closed | antidepressant |
| fracture | icd10 | S82891 | Fracture of ankle NOS, open | antidepressant |
| fracture | icd10 | S829 | Fracture of lower leg, part unspecified | antidepressant |
| fracture | icd10 | S82900 | Fracture of lower leg, part unspecified, closed | antidepressant |
| fracture | icd10 | S82901 | Fracture of lower leg, part unspecified, open | antidepressant |
| fracture | icd10 | S92 | Fracture of foot, except ankle | antidepressant |
| fracture | icd10 | S920 | Fracture of calcaneus | antidepressant |
| fracture | icd10 | S92000 | Fracture of calcaneus, closed | antidepressant |
| fracture | icd10 | S92001 | Fracture of calcaneus, open | antidepressant |
| fracture | icd10 | S921 | Fracture of talus | antidepressant |
| fracture | icd10 | S92100 | Fracture of talus, closed | antidepressant |
| fracture | icd10 | S92101 | Fracture of talus, open | antidepressant |
| fracture | icd10 | S922 | Fracture of other tarsal bone(s) | antidepressant |
| fracture | icd10 | S9220 | Fracture of navicular bone | antidepressant |
| fracture | icd10 | S92200 | Fracture of navicular bone, closed | antidepressant |
| fracture | icd10 | S92201 | Fracture of navicular bone, open | antidepressant |
| fracture | icd10 | S9221 | Fracture of cuboid bone | antidepressant |
| fracture | icd10 | S92210 | Fracture of cuboid bone, closed | antidepressant |
| fracture | icd10 | S92211 | Fracture of cuboid bone, open | antidepressant |
| fracture | icd10 | S9222 | Fracture of cuneiform bone | antidepressant |
| fracture | icd10 | S92220 | Fracture of cuneiform bone, closed | antidepressant |
| fracture | icd10 | S92221 | Fracture of cuneiform bone, open | antidepressant |
| fracture | icd10 | S9229 | Fracture of unspecified tarsal bone | antidepressant |
| fracture | icd10 | S92290 | Fracture of unspecified tarsal bone, closed | antidepressant |
| fracture | icd10 | S92291 | Fracture of unspecified tarsal bone, open | antidepressant |
| fracture | icd10 | S923 | Fracture of metatarsal bone | antidepressant |
| fracture | icd10 | S92300 | Fracture of metatarsal bone, closed | antidepressant |
| fracture | icd10 | S92301 | Fracture of metatarsal bone, open | antidepressant |
| fracture | icd10 | S924 | Fracture of great toe | antidepressant |
| fracture | icd10 | S92400 | Fracture of great toe, closed | antidepressant |
| fracture | icd10 | S92401 | Fracture of great toe, open | antidepressant |
| fracture | icd10 | S925 | Fracture of other toe | antidepressant |
| fracture | icd10 | S92500 | Fracture of other toe, closed | antidepressant |
| fracture | icd10 | S92501 | Fracture of other toe, open | antidepressant |
| fracture | icd10 | S927 | Multiple fractures of foot | antidepressant |
| fracture | icd10 | S92700 | Multiple fractures of foot, closed | antidepressant |
| fracture | icd10 | S92701 | Multiple fractures of foot, open | antidepressant |
| fracture | icd10 | S929 | Fracture of foot, unspecified | antidepressant |
| fracture | icd10 | S92900 | Fracture of foot, unspecified, closed | antidepressant |
| fracture | icd10 | S92901 | Fracture of foot, unspecified, open | antidepressant |
| fracture | icd10 | T02 | Fractures involving multiple body regions | antidepressant |
| fracture | icd10 | T020 | Fractures involving head with neck | antidepressant |
| fracture | icd10 | T0200 | Fractures involving head with neck, closed | antidepressant |
| fracture | icd10 | T0201 | Fractures involving head with neck, open | antidepressant |
| fracture | icd10 | T021 | Fractures involving thorax with lower back and pelvis | antidepressant |
| fracture | icd10 | T0210 | Fractures involving thorax with lower back and pelvis, closed | antidepressant |
| fracture | icd10 | T0211 | Fractures involving thorax with lower back and pelvis, open | antidepressant |
| fracture | icd10 | T022 | Fractures involving multiple regions of one upper limb | antidepressant |
| fracture | icd10 | T0220 | Fractures involving multiple regions of one upper limb, closed | antidepressant |
| fracture | icd10 | T0221 | Fractures involving multiple regions of one upper limb, open | antidepressant |
| fracture | icd10 | T023 | Fractures involving multiple regions of one lower limb | antidepressant |
| fracture | icd10 | T0230 | Fractures involving multiple regions of one lower limb, closed | antidepressant |
| fracture | icd10 | T0231 | Fractures involving multiple regions of one lower limb, open | antidepressant |
| fracture | icd10 | T024 | Fractures involving multiple regions of both upper limbs | antidepressant |
| fracture | icd10 | T0240 | Fractures involving multiple regions of both upper limbs, closed | antidepressant |
| fracture | icd10 | T0241 | Fractures involving multiple regions of both upper limbs, open | antidepressant |
| fracture | icd10 | T025 | Fractures involving multiple regions of both lower limbs | antidepressant |
| fracture | icd10 | T0250 | Fractures involving multiple regions of both lower limbs, closed | antidepressant |
| fracture | icd10 | T0251 | Fractures involving multiple regions of both lower limbs, open | antidepressant |
| fracture | icd10 | T026 | Fractures involving multiple regions of upper limb(s) | antidepressant |
| fracture | icd10 | T0260 | Fractures involving multiple regions of upper limb(s) with lower limb(s), closed | antidepressant |
| fracture | icd10 | T0261 | Fractures involving multiple regions of upper limb(s) with lower limb(s), open | antidepressant |
| fracture | icd10 | T027 | Fractures involving thorax with lower back and pelvis with limb(s) | antidepressant |
| fracture | icd10 | T0270 | Fractures involving thorax with lower back and pelvis with limb(s), closed | antidepressant |
| fracture | icd10 | T0271 | Fractures involving thorax with lower back and pelvis with limb(s), open | antidepressant |
| fracture | icd10 | T028 | Fractures involving other combinations of body regions | antidepressant |
| fracture | icd10 | T0280 | Fractures involving other combinations of body regions, closed | antidepressant |
| fracture | icd10 | T0281 | Fractures involving other combinations of body regions, open | antidepressant |
| fracture | icd10 | T029 | Multiple fractures, unspecified | antidepressant |
| fracture | icd10 | T0290 | Multiple fractures, unspecified, closed | antidepressant |
| fracture | icd10 | T0291 | Multiple fractures, unspecified, open | antidepressant |
| fracture | icd10 | T08 | Fracture of spine, level unspecified | antidepressant |
| fracture | icd10 | T080 | Fracture of spine, level unspecified, closed | antidepressant |
| fracture | icd10 | T081 | Fracture of spine, level unspecified, open | antidepressant |
| fracture | icd10 | T10 | Fracture of upper limb, level unspecified | antidepressant |
| fracture | icd10 | T100 | Fracture of upper limb, level unspecified, closed | antidepressant |
| fracture | icd10 | T101 | Fracture of upper limb, level unspecified, open | antidepressant |
| fracture | icd10 | T12 | Fracture of lower limb, level unspecified | antidepressant |
| fracture | icd10 | T120 | Fracture of lower limb, level unspecified, closed | antidepressant |
| fracture | icd10 | T121 | Fracture of lower limb, level unspecified, open | antidepressant |
| fracture | icd10 | T142 | Fracture of unspecified body region | antidepressant |
| fracture | icd10 | T1420 | Fracture of unspecified body region, closed | antidepressant |
| fracture | icd10 | T1421 | Fracture of unspecified body region, open | antidepressant |
| headache | icd10 | G444 | Drug-induced headache, not elsewhere classified | antidepressant |
| headache | icd10 | G448 | Other specified headache syndromes | antidepressant |
| headache | icd10 | R51 | Headache | antidepressant |
| insomnia | icd10 | F510 | Nonorganic insomnia | antidepressant |
| insomnia | icd10 | F518 | Other nonorganic sleep disorders | antidepressant |
| insomnia | icd10 | F519 | Nonorganic sleep disorder, unspecified | antidepressant |
| insomnia | icd10 | G470 | Disorders of initiating and maintaining sleep [insomnias] | antidepressant |
| insomnia | icd10 | G478 | Other sleep disorders | antidepressant |
| insomnia | icd10 | G479 | Sleep disorder, unspecified | antidepressant |
| ischemic stroke | icd10 | G464 | Cerebellar stroke syndrome | antidepressant |
| ischemic stroke | icd10 | I630 | Cerebral infarction due to thrombosis of precerebral arteries | antidepressant |
| ischemic stroke | icd10 | I631 | Cerebral infarction due to embolism of precerebral arteries | antidepressant |
| ischemic stroke | icd10 | I632 | Cerebral infarction due to unspecified occlusion or stenosis of precerebral arteries | antidepressant |
| ischemic stroke | icd10 | I633 | Cerebral infarction due to thrombosis of cerebral arteries | antidepressant |
| ischemic stroke | icd10 | I634 | Cerebral infarction due to embolism of cerebral arteries | antidepressant |
| ischemic stroke | icd10 | I635 | Cerebral infarction due to unspecified occlusion or stenosis of cerebral arteries | antidepressant |
| ischemic stroke | icd10 | I638 | Other cerebral infarction | antidepressant |
| ischemic stroke | icd10 | I639 | Cerebral infarction, unspecified | antidepressant |
| ischemic stroke | icd10 | I679 | Cerebrovascular disease, unspecified | antidepressant |
| nausea/vomiting | icd10 | R111 | Nausea alone | antidepressant |
| nausea/vomiting | icd10 | R112 | Vomiting alone | antidepressant |
| nausea/vomiting | icd10 | R113 | Nausea with vomiting | antidepressant |
| poisoning by antidep | icd10 | T430 | Poisoning by tricyclic and tetracyclic antidepressants | antidepressant |
| poisoning by antidep | icd10 | T431 | Poisoning by monoamine-oxidase-inhibitor antidepressants | antidepressant |
| poisoning by antidep | icd10 | T432 | Poisoning by other and unspecified antidepressants | antidepressant |
| sexual dysfunction | icd10 | F520 | Lack or loss of sexual desire | antidepressant |
| sexual dysfunction | icd10 | F521 | Sexual aversion and lack of sexual enjoyment | antidepressant |
| sexual dysfunction | icd10 | F522 | Failure of genital response | antidepressant |
| sexual dysfunction | icd10 | F523 | Orgasmic dysfunction | antidepressant |
| sexual dysfunction | icd10 | F524 | Premature ejaculation | antidepressant |
| sexual dysfunction | icd10 | F528 | Other sexual dysfunction, not caused by organic disorder or disease | antidepressant |
| sexual dysfunction | icd10 | F529 | Unspecified sexual dysfunction, not caused by organic disorder or disease | antidepressant |
| sexual dysfunction | icd10 | N4848 | Male erectile dysfunction due to other cause | antidepressant |
| sexual dysfunction | icd10 | N4849 | Male erectile dysfunction, unspecified | antidepressant |
| suicidality/self-harm | icd10 | X60 | Intentional self-poisoning by and exposure to nonopioid analgesics, antipyretics and antirheumatics | antidepressant |
| suicidality/self-harm | icd10 | X60-X8 | Intentional self-harm (X60-X84) | antidepressant |
| suicidality/self-harm | icd10 | X61 | Intentional self-poisoning by and exposure to antiepileptic, sedative-hypnotic, antiparkinsonism and psychotropic drugs, not elsewhere classified | antidepressant |
| suicidality/self-harm | icd10 | X62 | Intentional self-poisoning by and exposure to narcotics and psychodysleptics [hallucinogens], not elsewhere classified | antidepressant |
| suicidality/self-harm | icd10 | X63 | Intentional self-poisoning by and exposure to other drugs acting on the autonomic nervous system | antidepressant |
| suicidality/self-harm | icd10 | X64 | Intentional self-poisoning by and exposure to other and unspecified drugs, medicaments and biological substances | antidepressant |
| suicidality/self-harm | icd10 | X65 | Intentional self-poisoning by and exposure to alcohol | antidepressant |
| suicidality/self-harm | icd10 | X66 | Intentional self-poisoning by and exposure to organic solvents and halogenated hydrocarbons and their vapours | antidepressant |
| suicidality/self-harm | icd10 | X67 | Intentional self-poisoning by and exposure to other gases and vapours | antidepressant |
| suicidality/self-harm | icd10 | X68 | Intentional self-poisoning by and exposure to pesticides | antidepressant |
| suicidality/self-harm | icd10 | X69 | Intentional self-poisoning by and exposure to other and unspecified chemicals and noxious substances | antidepressant |
| suicidality/self-harm | icd10 | X70 | Intentional self-harm by hanging, strangulation and suffocation | antidepressant |
| suicidality/self-harm | icd10 | X71 | Intentional self-harm by drowning and submersion | antidepressant |
| suicidality/self-harm | icd10 | X72 | Intentional self-harm by handgun discharge | antidepressant |
| suicidality/self-harm | icd10 | X73 | Intentional self-harm by rifle, shotgun and larger firearm discharge | antidepressant |
| suicidality/self-harm | icd10 | X74 | Intentional self-harm by other and unspecified firearm discharge | antidepressant |
| suicidality/self-harm | icd10 | X7400 | Intentional self-harm by BB gun discharge | antidepressant |
| suicidality/self-harm | icd10 | X7401 | Intentional self-harm by air gun discharge | antidepressant |
| suicidality/self-harm | icd10 | X7408 | Intentional self-harm by other specified firearm discharge | antidepressant |
| suicidality/self-harm | icd10 | X7409 | Intentional self-harm by unspecified firearm discharge | antidepressant |
| suicidality/self-harm | icd10 | X75 | Intentional self-harm by explosive material | antidepressant |
| suicidality/self-harm | icd10 | X76 | Intentional self-harm by smoke, fire and flames | antidepressant |
| suicidality/self-harm | icd10 | X77 | Intentional self-harm by steam, hot vapours and hot objects | antidepressant |
| suicidality/self-harm | icd10 | X78 | Intentional self-harm by sharp object | antidepressant |
| suicidality/self-harm | icd10 | X79 | Intentional self-harm by blunt object | antidepressant |
| suicidality/self-harm | icd10 | X80 | Intentional self-harm by jumping from a high place | antidepressant |
| suicidality/self-harm | icd10 | X81 | Intentional self-harm by jumping or lying before moving object | antidepressant |
| suicidality/self-harm | icd10 | X82 | Intentional self-harm by crashing of motor vehicle | antidepressant |
| suicidality/self-harm | icd10 | X83 | Intentional self-harm by other specified means | antidepressant |
| suicidality/self-harm | icd10 | X84 | Intentional self-harm by unspecified means | antidepressant |
| suicidality/self-harm | icd10 | Y22 | Handgun discharge, undetermined intent | antidepressant |
| suicidality/self-harm | icd10 | Y23 | Rifle, shotgun and larger firearm discharge, undetermined intent | antidepressant |
| suicidality/self-harm | icd10 | Y2400 | BB gun discharge, undetermined intent | antidepressant |
| suicidality/self-harm | icd10 | Y2401 | Air gun discharge, undetermined intent | antidepressant |
| suicidality/self-harm | icd10 | Y2408 | Other specified firearm discharge, undetermined intent | antidepressant |
| suicidality/self-harm | icd10 | Y2409 | Unspecified firearm discharge, undetermined intent | antidepressant |
| suicidality/self-harm | icd10 | Y25 | Contact with explosive material, undetermined intent | antidepressant |
| suicidality/self-harm | icd10 | Y26 | Exposure to smoke, fire and flames, undetermined intent | antidepressant |
| suicidality/self-harm | icd10 | Y27 | Contact with steam, hot vapours and hot objects, undetermined intent | antidepressant |
| suicidality/self-harm | icd10 | Y28 | Contact with sharp object, undetermined intent | antidepressant |
| suicidality/self-harm | icd10 | Y29 | Contact with blunt object, undetermined intent | antidepressant |
| suicidality/self-harm | icd10 | Y30 | Falling, jumping or pushed from a high place, undetermined intent | antidepressant |
| suicidality/self-harm | icd10 | Y31 | Falling, lying or running before or into moving object, undetermined intent | antidepressant |
| suicidality/self-harm | icd10 | Y32 | Crashing of motor vehicle, undetermined intent | antidepressant |
| suicidality/self-harm | icd10 | Y33 | Other specified events, undetermined intent | antidepressant |
| suicidality/self-harm | icd10 | Y34 | Unspecified event, undetermined intent | antidepressant |
| suicidality/self-harm | icd10 | Y870 | Sequelae of intentional self-harm | antidepressant |
| sweating | icd10 | R611 | Generalized hyperhidrosis | antidepressant |
| sweating | icd10 | R619 | Hyperhidrosis, unspecified | antidepressant |
| tremor | icd10 | G250 | Essential tremor | antidepressant |
| tremor | icd10 | G251 | Drug-induced tremor | antidepressant |
| tremor | icd10 | G252 | Other specified forms of tremor | antidepressant |
| tremor | icd10 | R251 | Tremor, unspecified | antidepressant |
| upper GI tract bleeding | icd10 | K250 | Gastric ulcer, acute with haemorrhage | antidepressant |
| upper GI tract bleeding | icd10 | K251 | Gastric ulcer, acute with perforation | antidepressant |
| upper GI tract bleeding | icd10 | K252 | Gastric ulcer, acute with both haemorrhage and perforation | antidepressant |
| upper GI tract bleeding | icd10 | K253 | Gastric ulcer, acute without haemorrhage or perforation | antidepressant |
| upper GI tract bleeding | icd10 | K254 | Gastric ulcer, chronic or unspecified with haemorrhage | antidepressant |
| upper GI tract bleeding | icd10 | K255 | Gastric ulcer, chronic or unspecified with perforation | antidepressant |
| upper GI tract bleeding | icd10 | K256 | Gastric ulcer, chronic or unspecified with both haemorrhage and perforation | antidepressant |
| upper GI tract bleeding | icd10 | K257 | Gastric ulcer, chronic without haemorrhage or perforation | antidepressant |
| upper GI tract bleeding | icd10 | K259 | Gastric ulcer, unspecified as acute or chronic, without haemorrhage or perforation | antidepressant |
| upper GI tract bleeding | icd10 | K260 | Duodenal ulcer, acute with haemorrhage | antidepressant |
| upper GI tract bleeding | icd10 | K261 | Duodenal ulcer, acute with perforation | antidepressant |
| upper GI tract bleeding | icd10 | K262 | Duodenal ulcer, acute with both haemorrhage and perforation | antidepressant |
| upper GI tract bleeding | icd10 | K263 | Duodenal ulcer, acute without haemorrhage or perforation | antidepressant |
| upper GI tract bleeding | icd10 | K264 | Duodenal ulcer, chronic or unspecified with haemorrhage | antidepressant |
| upper GI tract bleeding | icd10 | K265 | Duodenal ulcer, chronic or unspecified with perforation | antidepressant |
| upper GI tract bleeding | icd10 | K266 | Duodenal ulcer, chronic or unspecified with both haemorrhage and perforation | antidepressant |
| upper GI tract bleeding | icd10 | K267 | Duodenal ulcer, chronic without haemorrhage or perforation | antidepressant |
| upper GI tract bleeding | icd10 | K269 | Duodenal ulcer, unspecified as acute or chronic, without haemorrhage or perforation | antidepressant |
| upper GI tract bleeding | icd10 | K270 | Peptic ulcer, acute with haemorrhage | antidepressant |
| upper GI tract bleeding | icd10 | K271 | Peptic ulcer, acute with perforation | antidepressant |
| upper GI tract bleeding | icd10 | K272 | Peptic ulcer, acute with both haemorrhage and perforation | antidepressant |
| upper GI tract bleeding | icd10 | K273 | Peptic ulcer, acute without haemorrhage or perforation | antidepressant |
| upper GI tract bleeding | icd10 | K274 | Peptic ulcer, chronic or unspecified with haemorrhage | antidepressant |
| upper GI tract bleeding | icd10 | K275 | Peptic ulcer, chronic or unspecified with perforation | antidepressant |
| upper GI tract bleeding | icd10 | K276 | Peptic ulcer, chronic or unspecified with both haemorrhage and perforation | antidepressant |
| upper GI tract bleeding | icd10 | K277 | Peptic ulcer, chronic without haemorrhage or perforation | antidepressant |
| upper GI tract bleeding | icd10 | K279 | Peptic ulcer, unspecified as acute or chronic, without haemorrhage or perforation | antidepressant |
| upper GI tract bleeding | icd10 | K280 | Gastrojejunal ulcer, acute with haemorrhage | antidepressant |
| upper GI tract bleeding | icd10 | K281 | Gastrojejunal ulcer, acute with perforation | antidepressant |
| upper GI tract bleeding | icd10 | K282 | Gastrojejunal ulcer, acute with both haemorrhage and perforation | antidepressant |
| upper GI tract bleeding | icd10 | K283 | Gastrojejunal ulcer, acute without haemorrhage or perforation | antidepressant |
| upper GI tract bleeding | icd10 | K284 | Gastrojejunal ulcer, chronic or unspecified with haemorrhage | antidepressant |
| upper GI tract bleeding | icd10 | K285 | Gastrojejunal ulcer, chronic or unspecified with perforation | antidepressant |
| upper GI tract bleeding | icd10 | K286 | Gastrojejunal ulcer, chronic or unspecified with both haemorrhage and perforation | antidepressant |
| upper GI tract bleeding | icd10 | K287 | Gastrojejunal ulcer, chronic without haemorrhage or perforation | antidepressant |
| upper GI tract bleeding | icd10 | K289 | Gastrojejunal ulcer, unspecified as acute or chronic, without haemorrhage or perforation | antidepressant |
| upper GI tract bleeding | icd10 | K920 | Haematemesis | antidepressant |
| upper GI tract bleeding | icd10 | K921 | Melaena | antidepressant |
| upper GI tract bleeding | icd10 | K922 | Gastrointestinal haemorrhage, unspecified | antidepressant |
| weight gain | icd10 | R635 | Abnormal weight gain | antidepressant |
| AE by antidep | icd9 | E9390 | Antidepressants causing adverse effects in therapeutic use | antidepressant |
| diarrhea | icd9 | 7879 | Symptoms involving digestive system, other (diarrhea) | antidepressant |
| diarrhea | icd9 | 55899 | Other and unspecified noninfectious gastroenteritis and colitis | antidepressant |
| dizziness | icd9 | 3862 | Vertigo of central origin | antidepressant |
| dizziness | icd9 | 7804 | General symptoms, dizziness and giddiness | antidepressant |
| dry mouth | icd9 | 5277 | Diseases of the salivary glands, disturbance of salivary secretion | antidepressant |
| fall | icd9 | E8810 | Fall from ladder | antidepressant |
| fall | icd9 | E8811 | Fall from scaffolding | antidepressant |
| fall | icd9 | E8829 | Accidental fall from or out of building or other structure. | antidepressant |
| fall | icd9 | E8831 | Accidental fall into well | antidepressant |
| fall | icd9 | E8832 | Accidental fall into storm drain or manhole | antidepressant |
| fall | icd9 | E8839 | Fall into other hole or other opening in surface | antidepressant |
| fall | icd9 | E8840 | Fall from playground equipment | antidepressant |
| fall | icd9 | E8841 | Fall from cliff | antidepressant |
| fall | icd9 | E8842 | Fall from chair | antidepressant |
| fall | icd9 | E8849 | Other fall from one level to another | antidepressant |
| fall | icd9 | E8859 | Fall from other slipping, tripping, or stumbling | antidepressant |
| fall | icd9 | E8889 | Fall, unspecified | antidepressant |
| fatigue | icd9 | 7807 | Malaise and fatigue | antidepressant |
| fatigue | icd9 | 78009 | Other alteration of consciousness | antidepressant |
| fracture | icd9 | 8000 | Fracture of vault of skull, closed without mention of intracranial injury | antidepressant |
| fracture | icd9 | 8001 | Fracture of vault of skull, closed with intracranial injury | antidepressant |
| fracture | icd9 | 8002 | Fracture of vault of skull, open without mention of intracranial injury | antidepressant |
| fracture | icd9 | 8003 | Fracture of vault of skull, open with intracranial injury | antidepressant |
| fracture | icd9 | 8009 | Fracture of vault of skull, fracture of vault of skull unspecified | antidepressant |
| fracture | icd9 | 8010 | Fracture of base of skull, closed without mention of intracranial injury | antidepressant |
| fracture | icd9 | 8011 | Fracture of base of skull, closed with intracranial injury | antidepressant |
| fracture | icd9 | 8012 | Fracture of base of skull, open without mention of intracranial injury | antidepressant |
| fracture | icd9 | 8013 | Fracture of base of skull, open with intracranial injury | antidepressant |
| fracture | icd9 | 8019 | Fracture of base of skull, fracture of base of skull unspecified | antidepressant |
| fracture | icd9 | 8020 | Fracture of face bones, nasal bones, closed | antidepressant |
| fracture | icd9 | 8021 | Fracture of face bones, nasal bones, open | antidepressant |
| fracture | icd9 | 8022 | Fracture of face bones, mandible, closed | antidepressant |
| fracture | icd9 | 8023 | Fracture of face bones, mandible, open | antidepressant |
| fracture | icd9 | 8024 | Fracture of face bones, malar and maxillary bones, closed | antidepressant |
| fracture | icd9 | 8025 | Fracture of face bones, malar and maxillary bones, open | antidepressant |
| fracture | icd9 | 8026 | Fracture of face bones, orbital floor (blow-out), closed | antidepressant |
| fracture | icd9 | 8027 | Fracture of face bones, orbital floor (blow-out), open | antidepressant |
| fracture | icd9 | 8028 | Fracture of face bones, other facial bones, closed | antidepressant |
| fracture | icd9 | 8029 | Fracture of face bones, other facial bones, open | antidepressant |
| fracture | icd9 | 8031 | Other and unqualified skull fractures, closed with intracranial injury | antidepressant |
| fracture | icd9 | 8033 | Other and unqualified skull fractures, open with intracranial injury | antidepressant |
| fracture | icd9 | 8039 | Other and unqualified skull fractures unspecified | antidepressant |
| fracture | icd9 | 8049 | Multiple fractures involving skull or face with other bones unspecified | antidepressant |
| fracture | icd9 | 8050 | Cervical, closed | antidepressant |
| fracture | icd9 | 8051 | Cervical, open | antidepressant |
| fracture | icd9 | 8052 | Closed fracture of dorsal [thoracic] vertebra without mention of spinal cord injury | antidepressant |
| fracture | icd9 | 8053 | Open fracture of dorsal [thoracic] vertebra without mention of spinal cord injury | antidepressant |
| fracture | icd9 | 8054 | Closed fracture of lumbar vertebra without mention of spinal cord injury | antidepressant |
| fracture | icd9 | 8055 | Open fracture of lumbar vertebra without mention of spinal cord injury | antidepressant |
| fracture | icd9 | 8056 | Closed fracture of sacrum and coccyx without mention of spinal cord injury | antidepressant |
| fracture | icd9 | 8057 | Open fracture of sacrum and coccyx without mention of spinal cord injury | antidepressant |
| fracture | icd9 | 8058 | Closed fracture of unspecified vertebral column without mention of spinal cord injury | antidepressant |
| fracture | icd9 | 8059 | Open fracture of unspecified vertebral column without mention of spinal cord injury | antidepressant |
| fracture | icd9 | 8060 | Fracture of vertebral column with spinal cord lesion, cervical, closed | antidepressant |
| fracture | icd9 | 8061 | Fracture of vertebral column with spinal cord lesion, cervical, open | antidepressant |
| fracture | icd9 | 8064 | Fracture of vertebral column with spinal cord lesion, lumbar, closed | antidepressant |
| fracture | icd9 | 8065 | Fracture of vertebral column with spinal cord lesion, lumbar, open | antidepressant |
| fracture | icd9 | 8068 | Fracture of vertebral column with spinal cord lesion, unspecified, closed | antidepressant |
| fracture | icd9 | 8069 | Fracture of vertebral column with spinal cord lesion, unspecified, open | antidepressant |
| fracture | icd9 | 8070 | Fracture of rib(s), closed | antidepressant |
| fracture | icd9 | 8071 | Fracture of rib(s), open | antidepressant |
| fracture | icd9 | 8072 | Fracture of sternum, closed | antidepressant |
| fracture | icd9 | 8073 | Fracture of sternum, open | antidepressant |
| fracture | icd9 | 8074 | Fracture of flail chest | antidepressant |
| fracture | icd9 | 8075 | Fracture of larynx and trachea, closed | antidepressant |
| fracture | icd9 | 8076 | Fracture of larynx and trachea, open | antidepressant |
| fracture | icd9 | 8079 | Fractures of ribs, sternum, larynx and trachea unspecified | antidepressant |
| fracture | icd9 | 8080 | Fracture of pelvis, acetabulum, closed | antidepressant |
| fracture | icd9 | 8081 | Fracture of pelvis, acetabulum, open | antidepressant |
| fracture | icd9 | 8082 | Fracture of pelvis, pubis, closed | antidepressant |
| fracture | icd9 | 8083 | Fracture of pelvis, pubis, open | antidepressant |
| fracture | icd9 | 8084 | Fracture of pelvis, other specified part, closed | antidepressant |
| fracture | icd9 | 8085 | Fracture of pelvis, other specified part, open | antidepressant |
| fracture | icd9 | 8088 | Fracture of pelvis, unspecified, closed | antidepressant |
| fracture | icd9 | 8089 | Fracture of pelvis, unspecified, open | antidepressant |
| fracture | icd9 | 8090 | Ill-defined fractures of trunk, closed | antidepressant |
| fracture | icd9 | 8091 | Ill-defined fractures of trunk, open | antidepressant |
| fracture | icd9 | 8099 | Ill-defined fractures of trunk unspecified | antidepressant |
| fracture | icd9 | 8100 | Fracture of clavicle, closed | antidepressant |
| fracture | icd9 | 8101 | Fracture of clavicle, open | antidepressant |
| fracture | icd9 | 8109 | Fracture of clavicle unspecified | antidepressant |
| fracture | icd9 | 8110 | Fracture of scapula, closed | antidepressant |
| fracture | icd9 | 8111 | Fracture of scapula, open | antidepressant |
| fracture | icd9 | 8119 | Fracture of scapula, unspecified | antidepressant |
| fracture | icd9 | 8120 | Fracture of humerus, upper end, closed | antidepressant |
| fracture | icd9 | 8121 | Fracture of humerus, upper end, open | antidepressant |
| fracture | icd9 | 8122 | Fracture of humerus, shaft or unspecified part, closed | antidepressant |
| fracture | icd9 | 8123 | Fracture of humerus, shaft or unspecified part, open | antidepressant |
| fracture | icd9 | 8124 | Fracture of humerus, lower end, closed | antidepressant |
| fracture | icd9 | 8125 | Fracture of humerus, lower end, open | antidepressant |
| fracture | icd9 | 8129 | Fracture of humerus, unspecified | antidepressant |
| fracture | icd9 | 8130 | Fracture of radius and ulna, upper end or unspecified part, closed | antidepressant |
| fracture | icd9 | 8131 | Fracture of radius and ulna, upper end or unspecified part, open | antidepressant |
| fracture | icd9 | 8132 | Fracture of radius and ulna, shaft, closed | antidepressant |
| fracture | icd9 | 8133 | Fracture of radius and ulna, shaft, open | antidepressant |
| fracture | icd9 | 8134 | Fracture of radius and ulna, lower end, closed | antidepressant |
| fracture | icd9 | 8135 | Fracture of radius and ulna, lower end, open | antidepressant |
| fracture | icd9 | 8139 | Fracture of radius and ulna, unspecified | antidepressant |
| fracture | icd9 | 8140 | Fracture of carpal bone(s), closed | antidepressant |
| fracture | icd9 | 8141 | Fracture of carpal bone(s), open | antidepressant |
| fracture | icd9 | 8149 | Fracture of carpal bone(s), unspecified | antidepressant |
| fracture | icd9 | 8150 | Fracture of metacarpal bone(s), closed | antidepressant |
| fracture | icd9 | 8151 | Fracture of metacarpal bone(s), open | antidepressant |
| fracture | icd9 | 8159 | Fracture of metacarpal bone(s), unspecified | antidepressant |
| fracture | icd9 | 8160 | Fracture of one or more phalanges of hand, closed | antidepressant |
| fracture | icd9 | 8161 | Fracture of one or more phalanges of hand, open | antidepressant |
| fracture | icd9 | 8169 | Fracture of one or more phalanges of hand unspecified | antidepressant |
| fracture | icd9 | 8170 | Multiple fractures of hand bones, closed | antidepressant |
| fracture | icd9 | 8171 | Multiple fractures of hand bones, open | antidepressant |
| fracture | icd9 | 8179 | Multiple fractures of hand bones unspecified | antidepressant |
| fracture | icd9 | 8180 | Ill-defined fractures of upper limb, closed | antidepressant |
| fracture | icd9 | 8181 | Ill-defined fractures of upper limb, open | antidepressant |
| fracture | icd9 | 8189 | Ill-defined fractures of upper limb, unspecified | antidepressant |
| fracture | icd9 | 8190 | Multiple closed fractures involving both upper limbs, and upper limb with rib(s) and sternum | antidepressant |
| fracture | icd9 | 8191 | Multiple open fractures involving both upper limbs, and upper limb with rib(s) and sternum | antidepressant |
| fracture | icd9 | 8199 | Multiple fractures of upper limbs unspecified | antidepressant |
| fracture | icd9 | 8200 | Fracture of neck of femur, transcervical fracture, closed | antidepressant |
| fracture | icd9 | 8201 | Fracture of neck of femur, transcervical fracture, open | antidepressant |
| fracture | icd9 | 8202 | Fracture of neck of femur, pertrochanteric fracture, closed | antidepressant |
| fracture | icd9 | 8203 | Fracture of neck of femur, pertrochanteric fracture, open | antidepressant |
| fracture | icd9 | 8208 | Fracture of neck of femur, unspecified part, closed | antidepressant |
| fracture | icd9 | 8209 | Fracture of neck of femur, unspecified part, open | antidepressant |
| fracture | icd9 | 8210 | Shaft or unspecified part, closed | antidepressant |
| fracture | icd9 | 8211 | Shaft or unspecified part, open | antidepressant |
| fracture | icd9 | 8212 | Fracture of other and unspecified parts of femur, lower end, closed | antidepressant |
| fracture | icd9 | 8213 | Fracture of other and unspecified parts of femur, lower end, open | antidepressant |
| fracture | icd9 | 8219 | Fracture of other and unspecified parts of femur | antidepressant |
| fracture | icd9 | 8220 | Fracture of patella, closed | antidepressant |
| fracture | icd9 | 8221 | Fracture of patella, open | antidepressant |
| fracture | icd9 | 8229 | Fracture of patella, unspecified | antidepressant |
| fracture | icd9 | 8230 | Fracture of tibia and fibula, upper end or unspecified part, closed | antidepressant |
| fracture | icd9 | 8231 | Fracture of tibia and fibula, upper end or unspecified part, open | antidepressant |
| fracture | icd9 | 8232 | Fracture of tibia and fibula, shaft, closed | antidepressant |
| fracture | icd9 | 8233 | Fracture of tibia and fibula, shaft, open | antidepressant |
| fracture | icd9 | 8239 | Fracture of tibia and fibula, unspecified | antidepressant |
| fracture | icd9 | 8240 | Fracture of ankle, medial malleolus, closed | antidepressant |
| fracture | icd9 | 8241 | Fracture of ankle, medial malleolus, open | antidepressant |
| fracture | icd9 | 8242 | Fracture of ankle, lateral malleolus, closed | antidepressant |
| fracture | icd9 | 8243 | Fracture of ankle, lateral malleolus, open | antidepressant |
| fracture | icd9 | 8244 | Fracture of ankle, bimalleolar, closed | antidepressant |
| fracture | icd9 | 8245 | Fracture of ankle, bimalleolar, open | antidepressant |
| fracture | icd9 | 8246 | Fracture of ankle, trimalleolar, closed | antidepressant |
| fracture | icd9 | 8247 | Fracture of ankle, trimalleolar, open | antidepressant |
| fracture | icd9 | 8248 | Fracture of ankle, unspecified, closed | antidepressant |
| fracture | icd9 | 8249 | Fracture of ankle, unspecified, open | antidepressant |
| fracture | icd9 | 8250 | Fracture of calcaneus, closed | antidepressant |
| fracture | icd9 | 8251 | Fracture of calcaneus, open | antidepressant |
| fracture | icd9 | 8252 | Fracture of other tarsal and metatarsal bones, closed | antidepressant |
| fracture | icd9 | 8253 | Fracture of other tarsal and metatarsal bones, open | antidepressant |
| fracture | icd9 | 8259 | Fracture of tarsal and metatarsal bones unspecified | antidepressant |
| fracture | icd9 | 8260 | Fracture of one or more phalanges of foot, closed | antidepressant |
| fracture | icd9 | 8261 | Fracture of one or more phalanges of foot, open | antidepressant |
| fracture | icd9 | 8269 | Fracture of phalanges of foot unspecified | antidepressant |
| fracture | icd9 | 8270 | Other, multiple and ill-defined fractures of lower limb, closed | antidepressant |
| fracture | icd9 | 8271 | Other, multiple and ill-defined fractures of lower limb, open | antidepressant |
| fracture | icd9 | 8279 | Other multiple and ill-defined fractures of lower limb unspecified | antidepressant |
| fracture | icd9 | 8280 | Closed | antidepressant |
| fracture | icd9 | 8281 | Open | antidepressant |
| fracture | icd9 | 8289 | Multiple fractures of lower limbs unspecified | antidepressant |
| fracture | icd9 | 8290 | Fracture of unspecified bones, closed | antidepressant |
| fracture | icd9 | 8291 | Fracture of unspecified bones, open | antidepressant |
| fracture | icd9 | 8299 | Fracture of unspecified bones | antidepressant |
| fracture | icd9 | 80000 | Closed fracture of vault of skull without mention of intracranial injury, unspecified state of consciousness | antidepressant |
| fracture | icd9 | 80001 | Closed fracture of vault of skull without mention of intracranial injury, with no loss of consciousness | antidepressant |
| fracture | icd9 | 80002 | Closed fracture of vault of skull without mention of intracranial injury, with brief [less than one hour] loss of consciousness | antidepressant |
| fracture | icd9 | 80003 | Closed fracture of vault of skull without mention of intracranial injury, with moderate [1-24 hours] loss of consciousness | antidepressant |
| fracture | icd9 | 80004 | Closed fracture of vault of skull without mention of intracranial injury, with prolonged [more than 24 hours] loss of consciousness and return to pre-existing conscious level | antidepressant |
| fracture | icd9 | 80005 | Closed fracture of vault of skull without mention of intracranial injury, with prolonged [more than 24 hours] loss of consciousness, without return to pre-existing conscious level | antidepressant |
| fracture | icd9 | 80006 | Closed fracture of vault of skull without mention of intracranial injury, with loss of consciousness of unspecified duration | antidepressant |
| fracture | icd9 | 80009 | Closed fracture of vault of skull without mention of intracranial injury, with concussion, unspecified | antidepressant |
| fracture | icd9 | 80010 | Closed fracture of vault of skull with cerebral laceration and contusion, unspecified state of consciousness | antidepressant |
| fracture | icd9 | 80011 | Closed fracture of vault of skull with cerebral laceration and contusion, with no loss of consciousness | antidepressant |
| fracture | icd9 | 80012 | Closed fracture of vault of skull with cerebral laceration and contusion, with brief [less than one hour] loss of consciousness | antidepressant |
| fracture | icd9 | 80013 | Closed fracture of vault of skull with cerebral laceration and contusion, with moderate [1-24 hours] loss of consciousness | antidepressant |
| fracture | icd9 | 80014 | Closed fracture of vault of skull with cerebral laceration and contusion, with prolonged [more than 24 hours] loss of consciousness and return to pre-existing conscious level | antidepressant |
| fracture | icd9 | 80015 | Closed fracture of vault of skull with cerebral laceration and contusion, with prolonged [more than 24 hours] loss of consciousness, without return to pre-existing conscious level | antidepressant |
| fracture | icd9 | 80016 | Closed fracture of vault of skull with cerebral laceration and contusion, with loss of consciousness of unspecified duration | antidepressant |
| fracture | icd9 | 80019 | Closed fracture of vault of skull with cerebral laceration and contusion, with concussion, unspecified | antidepressant |
| fracture | icd9 | 80020 | Fracture, voûte crâne, ouverte, sans traumatisme intracrânien, commotion¿ | antidepressant |
| fracture | icd9 | 80021 | Fracture, voûte crâne, ouverte, sans traumatisme intracrânien, sans coma | antidepressant |
| fracture | icd9 | 80022 | Fracture, voûte crâne, ouverte, sans traumatisme intracrânien, avec coma <1h | antidepressant |
| fracture | icd9 | 80023 | Fracture, voûte crâne, ouverte, sans traumatisme intracrânien, coma 1-24h | antidepressant |
| fracture | icd9 | 80024 | Fracture, voûte crâne, ouverte, sans traumatisme intracrânien, coma >24h r | antidepressant |
| fracture | icd9 | 80025 | Fracture, voûte crâne, ouverte, sans traumatisme intracrânien, coma >24h | antidepressant |
| fracture | icd9 | 80026 | Fracture, voûte crâne, ouverte, sans traumatisme intracrânien, coma SAI | antidepressant |
| fracture | icd9 | 80029 | Fracture, voûte crâne, ouverte, sans traumatisme intracrânien, état SAI | antidepressant |
| fracture | icd9 | 80030 | Fracture, voûte crâne, ouverte, avec traumatisme intracrânien, commotion¿ | antidepressant |
| fracture | icd9 | 80031 | Fracture, voûte crâne, ouverte, avec traumatisme intracrânien, sans coma | antidepressant |
| fracture | icd9 | 80032 | Fracture, voûte crâne, ouverte, avec traumatisme intracrânien, avec coma <1h | antidepressant |
| fracture | icd9 | 80033 | Fracture, voûte crâne, ouverte, avec traumatisme intracrânien, coma 1-24h | antidepressant |
| fracture | icd9 | 80034 | Fracture, voûte crâne, ouverte, avec traumatisme intracrânien, coma >24h r | antidepressant |
| fracture | icd9 | 80035 | Fracture, voûte crâne, ouverte, avec traumatisme intracrânien, coma >24h | antidepressant |
| fracture | icd9 | 80036 | Fracture, voûte crâne, ouverte, avec traumatisme intracrânien, coma SAI | antidepressant |
| fracture | icd9 | 80039 | Fracture, voûte crâne, ouverte, avec traumatisme intracrânien, état SAI | antidepressant |
| fracture | icd9 | 80100 | Closed fracture of base of skull without mention of intra cranial injury, unspecified state of consciousness | antidepressant |
| fracture | icd9 | 80101 | Closed fracture of base of skull without mention of intra cranial injury, with no loss of consciousness | antidepressant |
| fracture | icd9 | 80102 | Closed fracture of base of skull without mention of intra cranial injury, with brief [less than one hour] loss of consciousness | antidepressant |
| fracture | icd9 | 80103 | Closed fracture of base of skull without mention of intra cranial injury, with moderate [1-24 hours] loss of consciousness | antidepressant |
| fracture | icd9 | 80104 | Closed fracture of base of skull without mention of intra cranial injury, with prolonged [more than 24 hours] loss of consciousness and return to pre-existing conscious level | antidepressant |
| fracture | icd9 | 80105 | Closed fracture of base of skull without mention of intra cranial injury, with prolonged [more than 24 hours] loss of consciousness, without return to pre-existing conscious level | antidepressant |
| fracture | icd9 | 80106 | Closed fracture of base of skull without mention of intra cranial injury, with loss of consciousness of unspecified duration | antidepressant |
| fracture | icd9 | 80109 | Closed fracture of base of skull without mention of intra cranial injury, with concussion, unspecified | antidepressant |
| fracture | icd9 | 80110 | Closed fracture of base of skull with cerebral laceration and contusion, unspecified state of consciousness | antidepressant |
| fracture | icd9 | 80111 | Closed fracture of base of skull with cerebral laceration and contusion, with no loss of consciousness | antidepressant |
| fracture | icd9 | 80112 | Closed fracture of base of skull with cerebral laceration and contusion, with brief [less than one hour] loss of consciousness | antidepressant |
| fracture | icd9 | 80113 | Closed fracture of base of skull with cerebral laceration and contusion, with moderate [1-24 hours] loss of consciousness | antidepressant |
| fracture | icd9 | 80114 | Closed fracture of base of skull with cerebral laceration and contusion, with prolonged [more than 24 hours] loss of consciousness and return to pre-existing conscious level | antidepressant |
| fracture | icd9 | 80115 | Closed fracture of base of skull with cerebral laceration and contusion, with prolonged [more than 24 hours] loss of consciousness, without return to pre-existing conscious level | antidepressant |
| fracture | icd9 | 80116 | Closed fracture of base of skull with cerebral laceration and contusion, with loss of consciousness of unspecified duration | antidepressant |
| fracture | icd9 | 80119 | Closed fracture of base of skull with cerebral laceration and contusion, with concussion, unspecified | antidepressant |
| fracture | icd9 | 80120 | Fracture, base crâne, ouverte, sans traumatisme intracrânien, commotion¿ | antidepressant |
| fracture | icd9 | 80121 | Fracture, base crâne, ouverte, sans traumatisme intracrânien, sans coma | antidepressant |
| fracture | icd9 | 80122 | Fracture, base crâne, ouverte, sans traumatisme intracrânien, avec coma <1h | antidepressant |
| fracture | icd9 | 80123 | Fracture, base crâne, ouverte, sans traumatisme intracrânien, coma 1-24h | antidepressant |
| fracture | icd9 | 80124 | Fracture, base crâne, ouverte, sans traumatisme intracrânien, coma >24h r | antidepressant |
| fracture | icd9 | 80125 | Fracture, base crâne, ouverte, sans traumatisme intracrânien, coma >24h | antidepressant |
| fracture | icd9 | 80126 | Fracture, base crâne, ouverte, sans traumatisme intracrânien, coma SAI | antidepressant |
| fracture | icd9 | 80129 | Fracture, base crâne, ouverte, sans traumatisme intracrânien, état SAI | antidepressant |
| fracture | icd9 | 80130 | Fracture, base crâne, ouverte, avec traumatisme intracrânien, commotion¿ | antidepressant |
| fracture | icd9 | 80131 | Fracture, base crâne, ouverte, avec traumatisme intracrânien, sans coma | antidepressant |
| fracture | icd9 | 80132 | Fracture, base crâne, ouverte, avec traumatisme intracrânien, avec coma <1h | antidepressant |
| fracture | icd9 | 80133 | Fracture, base crâne, ouverte, avec traumatisme intracrânien, coma 1-24h | antidepressant |
| fracture | icd9 | 80134 | Fracture, base crâne, ouverte, avec traumatisme intracrânien, coma >24h r | antidepressant |
| fracture | icd9 | 80135 | Fracture, base crâne, ouverte, avec traumatisme intracrânien, coma >24h | antidepressant |
| fracture | icd9 | 80136 | Fracture, base crâne, ouverte, avec traumatisme intracrânien, coma SAI | antidepressant |
| fracture | icd9 | 80139 | Fracture, base crâne, ouverte, avec traumatisme intracrânien, état SAI | antidepressant |
| fracture | icd9 | 80300 | Other closed skull fracture without mention of intracranial injury, unspecified state of consciousness | antidepressant |
| fracture | icd9 | 80301 | Other closed skull fracture without mention of intracranial injury, with no loss of consciousness | antidepressant |
| fracture | icd9 | 80302 | Other closed skull fracture without mention of intracranial injury, with brief [less than one hour] loss of consciousness | antidepressant |
| fracture | icd9 | 80303 | Other closed skull fracture without mention of intracranial injury, with moderate [1-24 hours] loss of consciousness | antidepressant |
| fracture | icd9 | 80304 | Other closed skull fracture without mention of intracranial injury, with prolonged [more than 24 hours] loss of consciousness and return to pre-existing conscious level | antidepressant |
| fracture | icd9 | 80305 | Other closed skull fracture without mention of intracranial injury, with prolonged [more than 24 hours] loss of consciousness, without return to pre-existing conscious level | antidepressant |
| fracture | icd9 | 80306 | Other closed skull fracture without mention of intracranial injury, with loss of consciousness of unspecified duration | antidepressant |
| fracture | icd9 | 80309 | Other closed skull fracture without mention of intracranial injury, with concussion, unspecified | antidepressant |
| fracture | icd9 | 80310 | Other closed skull fracture with cerebral laceration and contusion, unspecified state of consciousness | antidepressant |
| fracture | icd9 | 80311 | Other closed skull fracture with cerebral laceration and contusion, with no loss of consciousness | antidepressant |
| fracture | icd9 | 80312 | Other closed skull fracture with cerebral laceration and contusion, with brief [less than one hour] loss of consciousness | antidepressant |
| fracture | icd9 | 80313 | Other closed skull fracture with cerebral laceration and contusion, with moderate [1-24 hours] loss of consciousness | antidepressant |
| fracture | icd9 | 80314 | Other closed skull fracture with cerebral laceration and contusion, with prolonged [more than 24 hours] loss of consciousness and return to pre-existing conscious level | antidepressant |
| fracture | icd9 | 80315 | Other closed skull fracture with cerebral laceration and contusion, with prolonged [more than 24 hours] loss of consciousness, without return to pre-existing conscious level | antidepressant |
| fracture | icd9 | 80316 | Other closed skull fracture with cerebral laceration and contusion, with loss of consciousness of unspecified duration | antidepressant |
| fracture | icd9 | 80319 | Other closed skull fracture with cerebral laceration and contusion, with concussion, unspecified | antidepressant |
| fracture | icd9 | 80320 | Fractures, crâne SAI, ouverte, sans traumatisme intracrânien, commotion¿ | antidepressant |
| fracture | icd9 | 80321 | Fractures, crâne SAI, ouverte, sans traumatisme intracrânien, sans coma | antidepressant |
| fracture | icd9 | 80322 | Fractures, crâne SAI, ouverte, sans traumatisme intracrânien, avec coma <1h | antidepressant |
| fracture | icd9 | 80323 | Fractures, crâne SAI, ouverte, sans traumatisme intracrânien, coma 1-24h | antidepressant |
| fracture | icd9 | 80324 | Fractures, crâne SAI, ouverte, sans traumatisme intracrânien, coma >24h r | antidepressant |
| fracture | icd9 | 80325 | Fractures, crâne SAI, ouverte, sans traumatisme intracrânien, coma >24h | antidepressant |
| fracture | icd9 | 80326 | Fractures, crâne SAI, ouverte, sans traumatisme intracrânien, coma SAI | antidepressant |
| fracture | icd9 | 80329 | Fractures, crâne SAI, ouverte, sans traumatisme intracrânien, état SAI | antidepressant |
| fracture | icd9 | 80330 | Fractures, crâne SAI, ouverte, avec traumatisme intracrânien, commotion¿ | antidepressant |
| fracture | icd9 | 80331 | Fractures, crâne SAI, ouverte, avec traumatisme intracrânien, sans coma | antidepressant |
| fracture | icd9 | 80332 | Fractures, crâne SAI, ouverte, avec traumatisme intracrânien, avec coma <1h | antidepressant |
| fracture | icd9 | 80333 | Fractures, crâne SAI, ouverte, avec traumatisme intracrânien, coma 1-24h | antidepressant |
| fracture | icd9 | 80334 | Fractures, crâne SAI, ouverte, avec traumatisme intracrânien, coma >24h r | antidepressant |
| fracture | icd9 | 80335 | Fractures, crâne SAI, ouverte, avec traumatisme intracrânien, coma >24h | antidepressant |
| fracture | icd9 | 80336 | Fractures, crâne SAI, ouverte, avec traumatisme intracrânien, coma SAI | antidepressant |
| fracture | icd9 | 80339 | Fractures, crâne SAI, ouverte, avec traumatisme intracrânien, état SAI | antidepressant |
| fracture | icd9 | 80400 | Closed fractures involving skull or face with other bones, without mention of intracranial injury, unspecified state of consciousness | antidepressant |
| fracture | icd9 | 80401 | Closed fractures involving skull or face with other bones, without mention of intracranial injury, with no loss of consciousness | antidepressant |
| fracture | icd9 | 80402 | Closed fractures involving skull or face with other bones, without mention of intracranial injury, with brief [less than one hour] loss of consciousness | antidepressant |
| fracture | icd9 | 80403 | Closed fractures involving skull or face with other bones, without mention of intracranial injury, with moderate [1-24 hours] loss of consciousness | antidepressant |
| fracture | icd9 | 80404 | Closed fractures involving skull or face with other bones, without mention or intracranial injury, with prolonged [more than 24 hours] loss of consciousness and return to pre-existing conscious level | antidepressant |
| fracture | icd9 | 80405 | Closed fractures involving skull of face with other bones, without mention of intracranial injury, with prolonged [more than 24 hours] loss of consciousness, without return to pre-existing conscious level | antidepressant |
| fracture | icd9 | 80406 | Closed fractures involving skull of face with other bones, without mention of intracranial injury, with loss of consciousness of unspecified duration | antidepressant |
| fracture | icd9 | 80409 | Closed fractures involving skull of face with other bones, without mention of intracranial injury, with concussion, unspecified | antidepressant |
| fracture | icd9 | 80410 | Closed fractures involving skull or face with other bones, with cerebral laceration and contusion, unspecified state of consciousness | antidepressant |
| fracture | icd9 | 80411 | Closed fractures involving skull or face with other bones, with cerebral laceration and contusion, with no loss of consciousness | antidepressant |
| fracture | icd9 | 80412 | Closed fractures involving skull or face with other bones, with cerebral laceration and contusion, with brief [less than one hour] loss of consciousness | antidepressant |
| fracture | icd9 | 80413 | Closed fractures involving skull or face with other bones, with cerebral laceration and contusion, with moderate [1-24 hours] loss of consciousness | antidepressant |
| fracture | icd9 | 80414 | Closed fractures involving skull or face with other bones, with cerebral laceration and contusion, with prolonged [more than 24 hours] loss of consciousness and return to pre-existing conscious level | antidepressant |
| fracture | icd9 | 80415 | Closed fractures involving skull or face with other bones, with cerebral laceration and contusion, with prolonged [more than 24 hours] loss of consciousness, without return to pre-existing conscious level | antidepressant |
| fracture | icd9 | 80416 | Closed fractures involving skull or face with other bones, with cerebral laceration and contusion, with loss of consciousness of unspecified duration | antidepressant |
| fracture | icd9 | 80419 | Closed fractures involving skull or face with other bones, with cerebral laceration and contusion, with concussion, unspecified | antidepressant |
| fracture | icd9 | 80420 | Fractures, crâne/face, ouverte, sans traumatisme intracrânien, commotion¿ | antidepressant |
| fracture | icd9 | 80421 | Fractures, crâne/face, ouverte, sans traumatisme intracrânien, sans coma | antidepressant |
| fracture | icd9 | 80422 | Fractures, crâne/face, ouverte, sans traumatisme intracrânien, avec coma <1h | antidepressant |
| fracture | icd9 | 80423 | Fractures, crâne/face, ouverte, sans traumatisme intracrânien, coma 1-24h | antidepressant |
| fracture | icd9 | 80424 | Fractures, crâne/face, ouverte, sans traumatisme intracrânien, coma >24h r | antidepressant |
| fracture | icd9 | 80425 | Fractures, crâne/face, ouverte, sans traumatisme intracrânien, coma >24h | antidepressant |
| fracture | icd9 | 80426 | Fractures, crâne/face, ouverte, sans traumatisme intracrânien, coma SAI | antidepressant |
| fracture | icd9 | 80429 | Fractures, crâne/face, ouverte, sans traumatisme intracrânien, état SAI | antidepressant |
| fracture | icd9 | 80430 | Fractures, crâne/face, ouverte, avec traumatisme intracrânien, commotion¿ | antidepressant |
| fracture | icd9 | 80431 | Fractures, crâne/face, ouverte, avec traumatisme intracrânien, sans coma | antidepressant |
| fracture | icd9 | 80432 | Fractures, crâne/face, ouverte, avec traumatisme intracrânien, avec coma <1h | antidepressant |
| fracture | icd9 | 80433 | Fractures, crâne/face, ouverte, avec traumatisme intracrânien, coma 1-24h | antidepressant |
| fracture | icd9 | 80434 | Fractures, crâne/face, ouverte, avec traumatisme intracrânien, coma >24h r | antidepressant |
| fracture | icd9 | 80435 | Fractures, crâne/face, ouverte, avec traumatisme intracrânien, coma >24h | antidepressant |
| fracture | icd9 | 80436 | Fractures, crâne/face, ouverte, avec traumatisme intracrânien, coma SAI | antidepressant |
| fracture | icd9 | 80439 | Fractures, crâne/face, ouverte, avec traumatisme intracrânien, état SAI | antidepressant |
| fracture | icd9 | 80701 | Fracture simple, une ou deux côtes | antidepressant |
| fracture | icd9 | 80702 | Fracture simple, trois ou quatre côtes | antidepressant |
| fracture | icd9 | 80703 | Fracture simple, cinq côtes et plus | antidepressant |
| fracture | icd9 | 80709 | Closed fracture of multiple ribs, unspecified | antidepressant |
| fracture | icd9 | 80711 | Fracture ouverte, une ou deux côtes | antidepressant |
| fracture | icd9 | 80712 | Fracture ouverte, trois ou quatre côtes | antidepressant |
| fracture | icd9 | 80713 | Fracture ouverte, cinq côtes et plus | antidepressant |
| fracture | icd9 | 80719 | Fracture ouverte, nombre de côtes non précisé | antidepressant |
| headache | icd9 | 3393 | Drug induced headache, not elsewhere classified | antidepressant |
| headache | icd9 | 7840 | Headache | antidepressant |
| headache | icd9 | 30781 | Tension headache | antidepressant |
| insomnia | icd9 | 3074 | Specific disorders of sleep | antidepressant |
| insomnia | icd9 | 78050 | Sleep disturbance, unspecified | antidepressant |
| insomnia | icd9 | 78052 | Insomnia, unspecified | antidepressant |
| insomnia | icd9 | 78059 | Other sleep disturbances | antidepressant |
| ischemic stroke | icd9 | 4369 | Acute but ill-defined cerebrovascular disease | antidepressant |
| ischemic stroke | icd9 | 4379 | Other and ill-defined cerebrovascular disease, unspecified | antidepressant |
| ischemic stroke | icd9 | 43391 | Occlusion and stenosis of unspecified precerebral artery with cerebral infarction | antidepressant |
| ischemic stroke | icd9 | 43401 | Cerebral thrombosis with cerebral infarction | antidepressant |
| ischemic stroke | icd9 | 43411 | Cerebral embolism with cerebral infarction | antidepressant |
| ischemic stroke | icd9 | 43491 | Cerebral artery occlusion, unspecified with cerebral infarction | antidepressant |
| nausea/vomiting | icd9 | 7870 | Symptoms involving digestive system, nausea and vomiting | antidepressant |
| poisoning by antidep | icd9 | 9690 | Poisoning by psychotropic agents, antidepressants | antidepressant |
| poisoning by antidep | icd9 | E8540 | Accidental poisoning by antidepressants | antidepressant |
| sexual dysfunction | icd9 | 3027 | Psychosexual dysfunction | antidepressant |
| sexual dysfunction | icd9 | 3029 | Sexual deviations and disorders, unspecified | antidepressant |
| sexual dysfunction | icd9 | 6078 | Disorders of penis, other | antidepressant |
| sexual dysfunction | icd9 | V417 | Problems with sexual function | antidepressant |
| suicidality/self-harm | icd9 | E9500 | Suicide and self-inflicted poisoning by analgesics, antipyretics, and antirheumatics | antidepressant |
| suicidality/self-harm | icd9 | E9501 | Suicide and self-inflicted poisoning by barbiturates | antidepressant |
| suicidality/self-harm | icd9 | E9502 | Suicide and self-inflicted poisoning by other sedatives and hypnotics | antidepressant |
| suicidality/self-harm | icd9 | E9503 | Suicide and self-inflicted poisoning by tranquilizers and other psychotropic agents | antidepressant |
| suicidality/self-harm | icd9 | E9504 | Suicide and self-inflicted poisoning by other specified drugs and medicinal substances | antidepressant |
| suicidality/self-harm | icd9 | E9505 | Suicide and self-inflicted poisoning by unspecified drug or medicinal substance | antidepressant |
| suicidality/self-harm | icd9 | E9506 | Suicide and self-inflicted poisoning by agricultural and horticultural chemical and pharmaceutical preparations other than plant foods and fertilizers | antidepressant |
| suicidality/self-harm | icd9 | E9507 | Suicide and self-inflicted poisoning by corrosive and caustic substances | antidepressant |
| suicidality/self-harm | icd9 | E9508 | Suicide and self-inflicted poisoning by arsenic and its compounds | antidepressant |
| suicidality/self-harm | icd9 | E9509 | Suicide and self-inflicted poisoning by other and unspecified solid and liquid substances | antidepressant |
| suicidality/self-harm | icd9 | E9510 | Suicide and self-inflicted poisoning by gas distributed by pipeline | antidepressant |
| suicidality/self-harm | icd9 | E9511 | Suicide and self-inflicted poisoning by liquefied petroleum gas distributed in mobile containers | antidepressant |
| suicidality/self-harm | icd9 | E9518 | Suicide and self-inflicted poisoning by other utility gas | antidepressant |
| suicidality/self-harm | icd9 | E9520 | Suicide and self-inflicted poisoning by motor vehicle exhaust gas | antidepressant |
| suicidality/self-harm | icd9 | E9521 | Suicide and self-inflicted poisoning by other carbon monoxide | antidepressant |
| suicidality/self-harm | icd9 | E9528 | Suicide and self-inflicted poisoning by other specified gases and vapors | antidepressant |
| suicidality/self-harm | icd9 | E9529 | Suicide and self-inflicted poisoning by unspecified gases and vapors | antidepressant |
| suicidality/self-harm | icd9 | E9530 | Suicide and self-inflicted injury by hanging | antidepressant |
| suicidality/self-harm | icd9 | E9531 | Suicide and self-inflicted injury by suffocation by plastic bag | antidepressant |
| suicidality/self-harm | icd9 | E9538 | Suicide and self-inflicted injury by other specified means | antidepressant |
| suicidality/self-harm | icd9 | E9539 | Suicide and self-inflicted injury by unspecified means | antidepressant |
| suicidality/self-harm | icd9 | E9549 | Suicide and self-inflicted injury by submersion [drowning] | antidepressant |
| suicidality/self-harm | icd9 | E9550 | Suicide and self-inflicted injury by handgun | antidepressant |
| suicidality/self-harm | icd9 | E9551 | Suicide and self-inflicted injury by shotgun | antidepressant |
| suicidality/self-harm | icd9 | E9552 | Suicide and self-inflicted injury by hunting rifle | antidepressant |
| suicidality/self-harm | icd9 | E9553 | Suicide and self-inflicted injury by military firearms | antidepressant |
| suicidality/self-harm | icd9 | E9554 | Suicide and self-inflicted injury by other and unspecified firearm | antidepressant |
| suicidality/self-harm | icd9 | E9555 | Suicide and self-inflicted injury by explosives | antidepressant |
| suicidality/self-harm | icd9 | E9559 | Suicide and self-inflicted injury by firearms and explosives, unspecified | antidepressant |
| suicidality/self-harm | icd9 | E9569 | Suicide and self-inflicted injury by cutting and piercing instrument | antidepressant |
| suicidality/self-harm | icd9 | E9570 | Suicide and self-inflicted injuries by jumping from residential premises | antidepressant |
| suicidality/self-harm | icd9 | E9571 | Suicide and self-inflicted injuries by jumping from other man-made structures | antidepressant |
| suicidality/self-harm | icd9 | E9572 | Suicide and self-inflicted injuries by jumping from natural sites | antidepressant |
| suicidality/self-harm | icd9 | E9579 | Suicide and self-inflicted injuries by jumping from unspecified site | antidepressant |
| suicidality/self-harm | icd9 | E9580 | Suicide and self-inflicted injury by jumping or lying before moving object | antidepressant |
| suicidality/self-harm | icd9 | E9581 | Suicide and self-inflicted injury by burns, fire | antidepressant |
| suicidality/self-harm | icd9 | E9582 | Suicide and self-inflicted injury by scald | antidepressant |
| suicidality/self-harm | icd9 | E9583 | Suicide and self-inflicted injury by extremes of cold | antidepressant |
| suicidality/self-harm | icd9 | E9584 | Suicide and self-inflicted injury by electrocution | antidepressant |
| suicidality/self-harm | icd9 | E9585 | Suicide and self-inflicted injury by crashing of motor vehicle | antidepressant |
| suicidality/self-harm | icd9 | E9586 | Suicide and self-inflicted injury by crashing of aircraft | antidepressant |
| suicidality/self-harm | icd9 | E9587 | Suicide and self-inflicted injury by caustic substances, except poisoning | antidepressant |
| suicidality/self-harm | icd9 | E9588 | Suicide and self-inflicted injury by other specified means | antidepressant |
| suicidality/self-harm | icd9 | E9589 | Suicide and self-inflicted injury by unspecified means | antidepressant |
| suicidality/self-harm | icd9 | E9599 | Late effects of self-inflicted injury | antidepressant |
| suicidality/self-harm | icd9 | E9850 | Injury by handgun, undetermined whether accidentally or purposely inflicted | antidepressant |
| suicidality/self-harm | icd9 | E9852 | Injury by hunting rifle, undetermined whether accidentally or purposely inflicted | antidepressant |
| suicidality/self-harm | icd9 | E9854 | Injury by other and unspecified firearm, undetermined whether accidentally or purposely inflicted | antidepressant |
| suicidality/self-harm | icd9 | E9855 | Injury by explosives, undetermined whether accidentally or purposely inflicted | antidepressant |
| suicidality/self-harm | icd9 | E9869 | Injury by cutting and piercing instruments, undetermined whether accidentally or purposely inflicted | antidepressant |
| suicidality/self-harm | icd9 | E9879 | Falling from unspecified site, undetermined whether accidentally or purposely inflicted | antidepressant |
| suicidality/self-harm | icd9 | E9880 | Injury by jumping or lying before moving object, undetermined whether accidentally or purposely inflicted | antidepressant |
| suicidality/self-harm | icd9 | E9881 | Injury by burns or fire, undetermined whether accidentally or purposely inflicted | antidepressant |
| suicidality/self-harm | icd9 | E9882 | Injury by scald, undetermined whether accidentally or purposely inflicted | antidepressant |
| suicidality/self-harm | icd9 | E9885 | Injury by crashing of motor vehicle, undetermined whether accidentally or purposely inflicted | antidepressant |
| suicidality/self-harm | icd9 | E9888 | Injury by other specified means, undetermined whether accidentally or purposely inflicted | antidepressant |
| suicidality/self-harm | icd9 | E9889 | Injury by unspecified means, undetermined whether accidentally or purposely inflicted | antidepressant |
| sweating | icd9 | 7808 | Generalized hyperhidrosis | antidepressant |
| tremor | icd9 | 3331 | Essential and other specified forms of tremor | antidepressant |
| tremor | icd9 | 7810 | Abnormal involuntary movements | antidepressant |
| upper GI tract bleeding | icd9 | 5310 | Gastric ulcer, acute with haemorrhage | antidepressant |
| upper GI tract bleeding | icd9 | 5311 | Gastric ulcer, acute with perforation | antidepressant |
| upper GI tract bleeding | icd9 | 5312 | Gastric ulcer, acute with haemorrhage and perforation | antidepressant |
| upper GI tract bleeding | icd9 | 5313 | Gastric ulcer, acute without mention of haemorrhage or perforation | antidepressant |
| upper GI tract bleeding | icd9 | 5314 | Gastric ulcer, chronic or unspecified with haemorrhage | antidepressant |
| upper GI tract bleeding | icd9 | 5315 | Gastric ulcer, chronic or unspecified with perforation | antidepressant |
| upper GI tract bleeding | icd9 | 5316 | Gastric ulcer, chronic or unspecified with haemorrhage and perforation | antidepressant |
| upper GI tract bleeding | icd9 | 5317 | Gastric ulcer, chronic without mention of haemorrhage or perforation | antidepressant |
| upper GI tract bleeding | icd9 | 5319 | Gastric ulcer, without mention of haemorrhage or perforation | antidepressant |
| upper GI tract bleeding | icd9 | 5320 | Duodenal ulcer, acute with haemorrhage | antidepressant |
| upper GI tract bleeding | icd9 | 5321 | Duodenal ulcer, acute with perforation | antidepressant |
| upper GI tract bleeding | icd9 | 5322 | Duodenal ulcer, acute with haemorrhage and perforation | antidepressant |
| upper GI tract bleeding | icd9 | 5323 | Duodenal ulcer, acute without mention of haemorrhage or perforation | antidepressant |
| upper GI tract bleeding | icd9 | 5324 | Duodenal ulcer, chronic or unspecified with haemorrhage | antidepressant |
| upper GI tract bleeding | icd9 | 5325 | Duodenal ulcer, chronic or unspecified with perforation | antidepressant |
| upper GI tract bleeding | icd9 | 5326 | Duodenal ulcer, chronic or unspecified with haemorrhage and perforation | antidepressant |
| upper GI tract bleeding | icd9 | 5327 | Duodenal ulcer, chronic without mention of haemorrhage or perforation | antidepressant |
| upper GI tract bleeding | icd9 | 5329 | Duodenal ulcer unspecified, without mention of haemorrhage or perforation | antidepressant |
| upper GI tract bleeding | icd9 | 5330 | Peptic ulcer, site unspecified, acute with haemorrhage | antidepressant |
| upper GI tract bleeding | icd9 | 5331 | Peptic ulcer, site unspecified, acute with perforation | antidepressant |
| upper GI tract bleeding | icd9 | 5332 | Peptic ulcer, site unspecified, acute with haemorrhage and perforation | antidepressant |
| upper GI tract bleeding | icd9 | 5333 | Acute without mention of haemorrhage or perforation | antidepressant |
| upper GI tract bleeding | icd9 | 5334 | Peptic ulcer, site unspecified, chronic or unspecified with haemorrhage | antidepressant |
| upper GI tract bleeding | icd9 | 5335 | Peptic ulcer, site unspecified, chronic or unspecified with perforation | antidepressant |
| upper GI tract bleeding | icd9 | 5336 | Chronic or unspecified with haemorrhage and perforation | antidepressant |
| upper GI tract bleeding | icd9 | 5337 | Chronic without mention of haemorrhage or perforation | antidepressant |
| upper GI tract bleeding | icd9 | 5339 | Peptic ulcer, unspecified, without mention of haemorrhage or perforation | antidepressant |
| upper GI tract bleeding | icd9 | 5340 | Gastrojejunal ulcer, acute with haemorrhage | antidepressant |
| upper GI tract bleeding | icd9 | 5341 | Gastrojejunal ulcer, acute with perforation | antidepressant |
| upper GI tract bleeding | icd9 | 5342 | Gastrojejunal ulcer, acute with haemorrhage and perforation | antidepressant |
| upper GI tract bleeding | icd9 | 5343 | Gastrojejunal ulcer, acute without mention of haemorrhage or perforation | antidepressant |
| upper GI tract bleeding | icd9 | 5344 | Gastrojejunal ulcer, chronic or unspecified with haemorrhage | antidepressant |
| upper GI tract bleeding | icd9 | 5345 | Gastrojejunal ulcer, chronic or unspecified with perforation | antidepressant |
| upper GI tract bleeding | icd9 | 5346 | Chronic or unspecified with haemorrhage and perforation | antidepressant |
| upper GI tract bleeding | icd9 | 5347 | Gastrojejunal ulcer, chronic without mention of haemorrhage or perforation | antidepressant |
| upper GI tract bleeding | icd9 | 5349 | Gastrojejunal ulcer, without mention of haemorrhage or perforation | antidepressant |
| upper GI tract bleeding | icd9 | 5780 | Gastrointestinal haemorrhage, haematemesis | antidepressant |
| upper GI tract bleeding | icd9 | 5781 | Melaena | antidepressant |
| upper GI tract bleeding | icd9 | 5789 | Haemorrhage of gastrointestinal tract, unspecified | antidepressant |
| weight gain | icd9 | 7831 | Abnormal weight gain | antidepressant |
| AE by antihyp | icd10 | Y514 | Predominantly alpha-adrenoreceptor agonists, not elsewhere classified, causing adverse effect in therapeutic use | antihypertensive |
| AE by antihyp | icd10 | Y516 | alpha-Adrenoreceptor antagonists, not elsewhere classified, causing adverse effect in therapeutic use | antihypertensive |
| AE by antihyp | icd10 | Y517 | beta-Adrenoreceptor antagonists, not elsewhere classified, causing adverse effect in therapeutic use | antihypertensive |
| AE by antihyp | icd10 | Y518 | Centrally acting and adrenergic-neuron-blocking agents, not elsewhere classified, causing adverse effect in therapeutic use | antihypertensive |
| AE by antihyp | icd10 | Y521 | Calcium-channel blockers causing adverse effect in therapeutic use | antihypertensive |
| AE by antihyp | icd10 | Y523 | Coronary vasodilators, not elsewhere classified, causing adverse effect in therapeutic use | antihypertensive |
| AE by antihyp | icd10 | Y524 | Angiotensin-converting-enzyme inhibitors causing adverse effect in therapeutic use | antihypertensive |
| AE by antihyp | icd10 | Y525 | Other antihypertensive drugs, not elsewhere classified, causing adverse effect in therapeutic use | antihypertensive |
| AE by antihyp | icd10 | Y527 | Peripheral vasodilators causing adverse effect in therapeutic use | antihypertensive |
| AE by antihyp | icd10 | Y541 | Mineralocorticoid antagonists [aldosterone antagonists] causing adverse effect in therapeutic use | antihypertensive |
| AE by antihyp | icd10 | Y542 | Carbonic-anhydrase inhibitors causing adverse effect in therapeutic use | antihypertensive |
| AE by antihyp | icd10 | Y544 | Loop [high-ceiling] diuretics causing adverse effect in therapeutic use | antihypertensive |
| AE by antihyp | icd10 | Y545 | Other diuretics causing adverse effect in therapeutic use | antihypertensive |
| angioedema | icd10 | T783 | Angioneurotic oedema | antihypertensive |
| bradycardia | icd10 | I44 | Atrioventricular and left bundle-branch block | antihypertensive |
| bradycardia | icd10 | I440 | Atrioventricular block, first degree | antihypertensive |
| bradycardia | icd10 | I441 | Atrioventricular block, second degree | antihypertensive |
| bradycardia | icd10 | I442 | Atrioventricular block, complete | antihypertensive |
| bradycardia | icd10 | I443 | Other and unspecified atrioventricular block | antihypertensive |
| bradycardia | icd10 | I444 | Left anterior fascicular block | antihypertensive |
| bradycardia | icd10 | I445 | Left posterior fascicular block | antihypertensive |
| bradycardia | icd10 | I446 | Other and unspecified fascicular block | antihypertensive |
| bradycardia | icd10 | I447 | Left bundle-branch block, unspecified | antihypertensive |
| bradycardia | icd10 | I458 | Other specified conduction disorders | antihypertensive |
| bradycardia | icd10 | I495 | Sick sinus syndrome | antihypertensive |
| bradycardia | icd10 | I498 | Other specified cardiac arrhythmias | antihypertensive |
| bradycardia | icd10 | R001 | Bradycardia, unspecified | antihypertensive |
| constipation | icd10 | K590 | Constipation | antihypertensive |
| cough | icd10 | R05 | Cough | antihypertensive |
| dizziness/vertigo | icd10 | H814 | Vertigo of central origin | antihypertensive |
| dizziness/vertigo | icd10 | R42 | Dizziness and giddiness | antihypertensive |
| dyspnea | icd10 | R060 | Dyspnoea | antihypertensive |
| fatigue | icd10 | R400 | Somnolence | antihypertensive |
| fatigue | icd10 | R53 | Malaise and fatigue | antihypertensive |
| flushing | icd10 | R232 | Flushing | antihypertensive |
| headache | icd10 | G444 | Drug-induced headache, not elsewhere classified | antihypertensive |
| headache | icd10 | G448 | Other specified headache syndromes | antihypertensive |
| headache | icd10 | R51 | Headache | antihypertensive |
| hepatotoxicity | icd10 | K710 | Toxic liver disease with cholestasis | antihypertensive |
| hepatotoxicity | icd10 | K711 | Toxic liver disease with hepatic necrosis | antihypertensive |
| hepatotoxicity | icd10 | K712 | Toxic liver disease with acute hepatitis | antihypertensive |
| hepatotoxicity | icd10 | K713 | Toxic liver disease with chronic persistent hepatitis | antihypertensive |
| hepatotoxicity | icd10 | K714 | Toxic liver disease with chronic lobular hepatitis | antihypertensive |
| hepatotoxicity | icd10 | K715 | Toxic liver disease with chronic active hepatitis | antihypertensive |
| hepatotoxicity | icd10 | K716 | Toxic liver disease with hepatitis, not elsewhere classified | antihypertensive |
| hepatotoxicity | icd10 | K717 | Toxic liver disease with fibrosis and cirrhosis of liver | antihypertensive |
| hepatotoxicity | icd10 | K718 | Toxic liver disease with other disorders of liver | antihypertensive |
| hepatotoxicity | icd10 | K719 | Toxic liver disease, unspecified | antihypertensive |
| hepatotoxicity | icd10 | K720 | Acute and subacute hepatic failure | antihypertensive |
| hepatotoxicity | icd10 | K752 | Nonspecific reactive hepatitis | antihypertensive |
| hepatotoxicity | icd10 | K754 | Autoimmune hepatitis | antihypertensive |
| hepatotoxicity | icd10 | K759 | Inflammatory liver disease, unspecified | antihypertensive |
| hepatotoxicity | icd10 | K762 | Central haemorrhagic necrosis of liver | antihypertensive |
| hepatotoxicity | icd10 | K768 | Other specified diseases of liver | antihypertensive |
| hepatotoxicity | icd10 | K769 | Liver disease, unspecified | antihypertensive |
| hepatotoxicity | icd10 | K838 | Other specified diseases of biliary tract | antihypertensive |
| hyopvolemia | icd10 | E868 | Other volume depletion | antihypertensive |
| hyperglycemia | icd10 | E110 | Type 2 diabetes mellitus with coma | antihypertensive |
| hyperglycemia | icd10 | E1120 | Type 2 diabetes mellitus with incipient diabetic nephropathy | antihypertensive |
| hyperglycemia | icd10 | E1121 | Type 2 diabetes mellitus with established diabetic nephropathy | antihypertensive |
| hyperglycemia | icd10 | E1122 | Type 2 diabetes mellitus with end-stage renal disease [ESRD] | antihypertensive |
| hyperglycemia | icd10 | E1123 | Type 2 diabetes mellitus with established or advanced kidney disease | antihypertensive |
| hyperglycemia | icd10 | E1128 | Type 2 diabetes mellitus with other specified renal complication not elsewhere classified | antihypertensive |
| hyperglycemia | icd10 | E119 | Type 2 diabetes mellitus without (mention of) complications | antihypertensive |
| hyperglycemia | icd10 | E130 | Other specified diabetes mellitus with coma | antihypertensive |
| hyperglycemia | icd10 | E1320 | Other specified diabetes mellitus with incipient diabetic nephropathy | antihypertensive |
| hyperglycemia | icd10 | E1321 | Other specified diabetes mellitus with established diabetic nephropathy | antihypertensive |
| hyperglycemia | icd10 | E1322 | Other specified diabetes mellitus with end-stage renal disease [ESRD] | antihypertensive |
| hyperglycemia | icd10 | E1323 | Other specified diabetes mellitus with established or advanced kidney disease | antihypertensive |
| hyperglycemia | icd10 | E1328 | Other specified diabetes mellitus with other specified renal complication not elsewhere classified | antihypertensive |
| hyperglycemia | icd10 | E139 | Other specified diabetes mellitus without (mention of) complication | antihypertensive |
| hyperglycemia | icd10 | E140 | Unspecified diabetes mellitus with coma | antihypertensive |
| hyperglycemia | icd10 | E1420 | Unspecified diabetes mellitus with incipient diabetic nephropathy | antihypertensive |
| hyperglycemia | icd10 | E1421 | Unspecified diabetes mellitus with established diabetic nephropathy | antihypertensive |
| hyperglycemia | icd10 | E1422 | Unspecified diabetes mellitus with end-stage renal disease [ESRD] | antihypertensive |
| hyperglycemia | icd10 | E1423 | Unspecified diabetes mellitus with established or advanced kidney disease | antihypertensive |
| hyperglycemia | icd10 | E1428 | Unspecified diabetes mellitus with other specified renal complication not elsewhere classified | antihypertensive |
| hyperglycemia | icd10 | E149 | Unspecified diabetes mellitus without (mention of) complication | antihypertensive |
| hyperglycemia | icd10 | R73 | Elevated blood glucose level | antihypertensive |
| hyperglycemia | icd10 | R730 | Abnormal glucose tolerance test | antihypertensive |
| hyperglycemia | icd10 | R738 | Other evidence of elevated blood glucose level | antihypertensive |
| hyperglycemia | icd10 | R73800 | Blood glucose between 8.0 - 11.9 mmol/L pre-meal (fasting) | antihypertensive |
| hyperglycemia | icd10 | R73801 | Blood glucose between 12.0 - 13.9 mmol/L pre-meal (fasting) | antihypertensive |
| hyperglycemia | icd10 | R73802 | Blood glucose greater than or equal to 14.0 mmol/L pre-meal (fasting) | antihypertensive |
| hyperglycemia | icd10 | R73811 | Blood glucose between 12.0 - 13.9 mmol/L post-meal (or NOS) | antihypertensive |
| hyperglycemia | icd10 | R73812 | Blood glucose greater than or equal to 14.0 mmol/L post-meal (or NOS) | antihypertensive |
| hyperglycemia | icd10 | R739 | Hyperglycaemia, unspecified | antihypertensive |
| hyperkalemia | icd10 | E875 | Hyperkalaemia | antihypertensive |
| hyperlipidemia | icd10 | E780 | Pure hypercholesterolaemia | antihypertensive |
| hyperlipidemia | icd10 | E781 | Pure hyperglyceridaemia | antihypertensive |
| hyperlipidemia | icd10 | E782 | Mixed hyperlipidaemia | antihypertensive |
| hyperlipidemia | icd10 | E783 | Hyperchylomicronaemia | antihypertensive |
| hyperlipidemia | icd10 | E784 | Other hyperlipidaemia | antihypertensive |
| hyperlipidemia | icd10 | E785 | Hyperlipidaemia, unspecified | antihypertensive |
| hypokalemia | icd10 | E876 | Hypokalaemia | antihypertensive |
| hypomagnesemia | icd10 | E834 | Disorders of magnesium metabolism | antihypertensive |
| hyponatremia | icd10 | E871 | Hypo-osmolality and hyponatraemia | antihypertensive |
| hypotension | icd10 | I950 | Idiopathic hypotension | antihypertensive |
| hypotension | icd10 | I951 | Orthostatic hypotension | antihypertensive |
| hypotension | icd10 | I952 | Hypotension due to drugs | antihypertensive |
| hypotension | icd10 | I958 | Other hypotension | antihypertensive |
| hypotension | icd10 | I959 | Hypotension, unspecified | antihypertensive |
| hypovolemia | icd10 | E86 | Volume depletion | antihypertensive |
| metabolic alkalosis | icd10 | E873 | Alkalosis | antihypertensive |
| nausea | icd10 | R111 | Nausea alone | antihypertensive |
| nausea | icd10 | R113 | Nausea with vomiting | antihypertensive |
| neutropenia | icd10 | D700 | Neutropenia | antihypertensive |
| peripheral edema | icd10 | R600 | Localized oedema | antihypertensive |
| peripheral edema | icd10 | R601 | Generalized oedema | antihypertensive |
| peripheral edema | icd10 | R609 | Oedema, unspecified | antihypertensive |
| photosensitivity dermatitis | icd10 | L560 | Drug phototoxic response | antihypertensive |
| photosensitivity dermatitis | icd10 | L561 | Drug photoallergic response | antihypertensive |
| poisoning by antihyp | icd10 | T444 | Poisoning by predominantly alpha-adrenoreceptor agonists, not elsewhere classified | antihypertensive |
| poisoning by antihyp | icd10 | T446 | Poisoning by alpha-adrenoreceptor antagonists, not elsewhere classified | antihypertensive |
| poisoning by antihyp | icd10 | T447 | Poisoning by beta-Adrenoreceptor antagonists, not elsewhere classified | antihypertensive |
| poisoning by antihyp | icd10 | T448 | Poisoning by centrally acting and adrenergic-neuron-blocking agents, not elsewhere classified | antihypertensive |
| poisoning by antihyp | icd10 | T461 | Poisoning by calcium-channel blockers | antihypertensive |
| poisoning by antihyp | icd10 | T463 | Poisoning by coronary vasodilators, not elsewhere classified | antihypertensive |
| poisoning by antihyp | icd10 | T464 | Poisoning by angiotensin-converting-enzyme inhibitors | antihypertensive |
| poisoning by antihyp | icd10 | T465 | Poisoning by other antihypertensive drugs, not elsewhere classified | antihypertensive |
| poisoning by antihyp | icd10 | T467 | Poisoning by peripheral vasodilators | antihypertensive |
| poisoning by antihyp | icd10 | T501 | Poisoning by loop [high-ceiling] diuretics | antihypertensive |
| poisoning by antihyp | icd10 | T502 | Poisoning by carbonic-anhydrase inhibitors, benzothiadiazides and other diuretics | antihypertensive |
| psoriasis | icd10 | L408 | Other psoriasis | antihypertensive |
| psoriasis | icd10 | L409 | Psoriasis, unspecified | antihypertensive |
| rash | icd10 | L270 | Generalized skin eruption due to drugs and medicaments | antihypertensive |
| rash | icd10 | L271 | Localized skin eruption due to drugs and medicaments | antihypertensive |
| rash | icd10 | L278 | Dermatitis due to other substances taken internally | antihypertensive |
| rash | icd10 | L279 | Dermatitis due to unspecified substance taken internally | antihypertensive |
| rash | icd10 | R21 | Rash and other nonspecific skin eruption | antihypertensive |
| renal impairment | icd10 | N170 | Acute renal failure with tubular necrosis | antihypertensive |
| renal impairment | icd10 | N171 | Acute renal failure with acute cortical necrosis | antihypertensive |
| renal impairment | icd10 | N172 | Acute renal failure with medullary necrosis | antihypertensive |
| renal impairment | icd10 | N178 | Other acute renal failure | antihypertensive |
| renal impairment | icd10 | N179 | Acute renal failure, unspecified | antihypertensive |
| renal impairment | icd10 | N19 | Unspecified renal failure | antihypertensive |
| renal impairment | icd10 | N289 | Disorder of kidney and ureter, unspecified | antihypertensive |
| sexual dysfunction | icd10 | F520 | Lack or loss of sexual desire | antihypertensive |
| sexual dysfunction | icd10 | F521 | Sexual aversion and lack of sexual enjoyment | antihypertensive |
| sexual dysfunction | icd10 | F522 | Failure of genital response | antihypertensive |
| sexual dysfunction | icd10 | F523 | Orgasmic dysfunction | antihypertensive |
| sexual dysfunction | icd10 | F524 | Premature ejaculation | antihypertensive |
| sexual dysfunction | icd10 | F528 | Other sexual dysfunction, not caused by organic disorder or disease | antihypertensive |
| sexual dysfunction | icd10 | F529 | Unspecified sexual dysfunction, not caused by organic disorder or disease | antihypertensive |
| sexual dysfunction | icd10 | N4848 | Male erectile dysfunction due to other cause | antihypertensive |
| sexual dysfunction | icd10 | N4849 | Male erectile dysfunction, unspecified | antihypertensive |
| taste disturbances | icd10 | R432 | Parageusia | antihypertensive |
| weight gain | icd10 | R635 | Abnormal weight gain | antihypertensive |
| AE by antihyp | icd9 | E9413 | Sympatholytics [antiadrenergics] causing adverse effects in therapeutic use | antihypertensive |
| AE by antihyp | icd9 | E9424 | Coronary vasodilators causing adverse effects in therapeutic use | antihypertensive |
| AE by antihyp | icd9 | E9425 | Other vasodilators causing adverse effects in therapeutic use | antihypertensive |
| AE by antihyp | icd9 | E9426 | Other antihypertensive agents causing adverse effects in therapeutic use | antihypertensive |
| AE by antihyp | icd9 | E9440 | Mercurial diuretics causing adverse effects in therapeutic use | antihypertensive |
| AE by antihyp | icd9 | E9441 | Purine derivative diuretics causing adverse effects in therapeutic use | antihypertensive |
| AE by antihyp | icd9 | E9442 | Carbonic acid anhydrase inhibitors causing adverse effects in therapeutic use | antihypertensive |
| AE by antihyp | icd9 | E9443 | Saluretics causing adverse effects in therapeutic use | antihypertensive |
| AE by antihyp | icd9 | E9444 | Other diuretics causing adverse effects in therapeutic use | antihypertensive |
| angioedema | icd9 | 9951 | Angioneurotic edema, not elsewhere classified | antihypertensive |
| bradycardia | icd9 | 4260 | Conduction disorders, atrioventricular block, complete | antihypertensive |
| bradycardia | icd9 | 4261 | Conduction disorders, atrioventricular block, other and unspecified | antihypertensive |
| bradycardia | icd9 | 4262 | Conduction disorders, left bundle branch hemiblock | antihypertensive |
| bradycardia | icd9 | 4263 | Conduction disorders, other left bundle branch block | antihypertensive |
| bradycardia | icd9 | 4264 | Conduction disorders, right bundle branch block | antihypertensive |
| bradycardia | icd9 | 4265 | Conduction disorders, bundle branch block, unspecified | antihypertensive |
| bradycardia | icd9 | 4266 | Conduction disorders, other heart block | antihypertensive |
| bradycardia | icd9 | 4267 | Conduction disorders, anomalous atrioventricular excitation | antihypertensive |
| bradycardia | icd9 | 4268 | Conduction disorders, other | antihypertensive |
| bradycardia | icd9 | 4269 | Conduction disorders, unspecified | antihypertensive |
| bradycardia | icd9 | 42610 | Atrioventricular block, unspecified | antihypertensive |
| bradycardia | icd9 | 42611 | First degree atrioventricular block | antihypertensive |
| bradycardia | icd9 | 42612 | Mobitz (type) II atrioventricular block | antihypertensive |
| bradycardia | icd9 | 42613 | Other second degree atrioventricular block | antihypertensive |
| bradycardia | icd9 | 42650 | Bundle branch block, unspecified | antihypertensive |
| bradycardia | icd9 | 42651 | Right bundle branch block and left posterior fascicular block | antihypertensive |
| bradycardia | icd9 | 42652 | Right bundle branch block and left anterior fascicular block | antihypertensive |
| bradycardia | icd9 | 42653 | Other bilateral bundle branch block | antihypertensive |
| bradycardia | icd9 | 42654 | Trifascicular block | antihypertensive |
| bradycardia | icd9 | 42781 | Sinoatrial node dysfunction | antihypertensive |
| bradycardia | icd9 | 42789 | Other specified cardiac dysrhythmias | antihypertensive |
| constipation | icd9 | 5640 | Functional digestive disorders, not elsewhere classified, constipation | antihypertensive |
| cough | icd9 | 7862 | Symptoms involving respiratory system and other chest symptoms, cough | antihypertensive |
| dizziness/vertigo | icd9 | 3862 | Vertigo of central origin | antihypertensive |
| dizziness/vertigo | icd9 | 7804 | General symptoms, dizziness and giddiness | antihypertensive |
| dyspnea | icd9 | 7860 | Dyspnea and respiratory abnormalities | antihypertensive |
| fatigue | icd9 | 7807 | Malaise and fatigue | antihypertensive |
| fatigue | icd9 | 78009 | Other alteration of consciousness | antihypertensive |
| flushing | icd9 | 7826 | Symptoms involving skin and other integumentary tissue, pallor and flushing | antihypertensive |
| headache | icd9 | 3393 | Drug induced headache, not elsewhere classified | antihypertensive |
| headache | icd9 | 7840 | Headache | antihypertensive |
| headache | icd9 | 30781 | Tension headache | antihypertensive |
| hepatotoxicity | icd9 | 5709 | Acute and subacute necrosis of liver | antihypertensive |
| hepatotoxicity | icd9 | 5733 | Hepatitis unspecified | antihypertensive |
| hepatotoxicity | icd9 | 5738 | Other disorders of liver | antihypertensive |
| hepatotoxicity | icd9 | 5739 | Disorders of liver, unspecified | antihypertensive |
| hepatotoxicity | icd9 | 5768 | Other disorders of biliary tract, other | antihypertensive |
| hyperglycemia | icd9 | 7902 | Abnormal glucose tolerance test | antihypertensive |
| hyperglycemia | icd9 | 25000 | Diabète sucré sans complication, adulte | antihypertensive |
| hyperglycemia | icd9 | 25009 | Diabète sucré sans complication, forme non précisée | antihypertensive |
| hyperglycemia | icd9 | 25020 | Diabète avec coma, adulte | antihypertensive |
| hyperglycemia | icd9 | 25029 | Diabète avec coma, forme non précisée | antihypertensive |
| hyperglycemia | icd9 | 25030 | Diabète avec complications rénales, adulte | antihypertensive |
| hyperglycemia | icd9 | 25039 | Diabète avec complications rénales, forme non précisée | antihypertensive |
| hyperkalemia | icd9 | 2767 | Hyperpotassemia | antihypertensive |
| hyperlipidemia | icd9 | 2720 | Disorders of lipoid metabolism, pure hypercholesterolaemia | antihypertensive |
| hyperlipidemia | icd9 | 2721 | Disorders of lipoid metabolism, pure hyperglyceridaemia | antihypertensive |
| hyperlipidemia | icd9 | 2722 | Disorders of lipoid metabolism, mixed hyperlipidaemia | antihypertensive |
| hyperlipidemia | icd9 | 2723 | Disorders of lipoid metabolism, hyperchylomicronaemia | antihypertensive |
| hyperlipidemia | icd9 | 2724 | Disorders of lipoid metabolism, other and unspecified hyperlipidaemia | antihypertensive |
| hypokalemia | icd9 | 2768 | Hypopotassemia | antihypertensive |
| hypomagnesemia | icd9 | 2752 | Disorders of mineral metabolism, disorders of magnesium metabolism | antihypertensive |
| hyponatremia | icd9 | 2761 | Hyposmolality and/or hyponatremia | antihypertensive |
| hypotension | icd9 | 4580 | Hypotension, orthostatic hypotension | antihypertensive |
| hypotension | icd9 | 4581 | Hypotension, chronic hypotension | antihypertensive |
| hypotension | icd9 | 4589 | Hypotension, unspecified | antihypertensive |
| hypotension | icd9 | 45829 | Other iatrogenic hypotension | antihypertensive |
| hypovalemia | icd9 | 2765 | Disorders of fluid, electrolyte and acid-base balance, volume depletion | antihypertensive |
| metabolic alkalosis | icd9 | 2763 | Disorders of fluid, electrolyte and acid-base balance, alkalosis | antihypertensive |
| nausea | icd9 | 7870 | Symptoms involving digestive system, nausea and vomiting | antihypertensive |
| neutropenia | icd9 | 2880 | Diseases of white blood cells, agranulocytosis | antihypertensive |
| peripheral edema | icd9 | 7823 | Edema | antihypertensive |
| photosensitivity dermatitis | icd9 | 6927 | Contact dermatitis and other eczema, due to solar radiation | antihypertensive |
| poisoning by antihyp | icd9 | 9713 | Poisoning by sympatholytics [antiadrenergics] | antihypertensive |
| poisoning by antihyp | icd9 | 9720 | Poisoning by cardiac rhythm regulators | antihypertensive |
| poisoning by antihyp | icd9 | 9724 | Poisoning by coronary vasodilators | antihypertensive |
| poisoning by antihyp | icd9 | 9725 | Poisoning by other vasodilators | antihypertensive |
| poisoning by antihyp | icd9 | 9726 | Poisoning by other antihypertensive agents | antihypertensive |
| poisoning by antihyp | icd9 | 9729 | Poisoning by other and unspecified agents primarily affecting the cardiovascular system | antihypertensive |
| poisoning by antihyp | icd9 | 9742 | Poisoning by carbonic acid anhydrase inhibitors | antihypertensive |
| poisoning by antihyp | icd9 | 9744 | Poisoning by other diuretics | antihypertensive |
| poisoning by antihyp | icd9 | E8556 | Accidental poisoning by sympatholytics [antiadrenergics] | antihypertensive |
| poisoning by antihyp | icd9 | E8585 | Accidental poisoning by water, mineral, and uric acid metabolism drugs | antihypertensive |
| psoriasis | icd9 | 6960 | Psoriasis and similar disorders, psoriatic arthropathy | antihypertensive |
| psoriasis | icd9 | 6961 | Psoriasis and similar disorders, other psoriasis | antihypertensive |
| rash | icd9 | 6930 | Dermatitis due to substances taken internally, due to drugs and medicaments | antihypertensive |
| rash | icd9 | 6938 | Dermatitis due to other specified substances taken internally | antihypertensive |
| rash | icd9 | 6939 | Dermatitis due to substances taken internally, due to unspecified substance | antihypertensive |
| rash | icd9 | 7821 | Rash and other nonspecific skin eruption | antihypertensive |
| renal impairment | icd9 | 5845 | Acute renal failure, with lesion of tubular necrosis | antihypertensive |
| renal impairment | icd9 | 5846 | Acute renal failure, with lesion of renal cortical necrosis | antihypertensive |
| renal impairment | icd9 | 5847 | Acute renal failure, with lesion of renal medullary (papillary) necrosis | antihypertensive |
| renal impairment | icd9 | 5848 | Acute renal failure, with other specified pathological lesion in kidney | antihypertensive |
| renal impairment | icd9 | 5849 | Acute renal failure, unspecified | antihypertensive |
| renal impairment | icd9 | 5869 | Renal failure, unspecified | antihypertensive |
| renal impairment | icd9 | 5939 | Other disorders of kidney and ureter, unspecified | antihypertensive |
| sexual dysfunction | icd9 | 3027 | Psychosexual dysfunction | antihypertensive |
| sexual dysfunction | icd9 | 3029 | Sexual deviations and disorders, unspecified | antihypertensive |
| sexual dysfunction | icd9 | 6078 | Disorders of penis, other | antihypertensive |
| sexual dysfunction | icd9 | V417 | Problems with sexual function | antihypertensive |
| taste disturbances | icd9 | 7811 | Disturbances of sensation of smell and taste | antihypertensive |
| weight gain | icd9 | 7831 | Abnormal weight gain | antihypertensive |

**Appendix 3. Standard ICD Code Set**

| Adverse Effect | Code Type | Code | Code Description | Medication Class |
| --- | --- | --- | --- | --- |
| AE by antidep | icd10 | Y490 | Tricyclic and tetracyclic antidepressants causing adverse effect in therapeutic use | antidepressant |
| AE by antidep | icd10 | Y491 | Monoamine-oxidase-inhibitor antidepressants causing adverse effect in therapeutic use | antidepressant |
| AE by antidep | icd10 | Y492 | Other and unspecified antidepressants causing adverse effect in therapeutic use | antidepressant |
| dizziness | icd10 | R42 | Dizziness and giddiness | antidepressant |
| headache | icd10 | G444 | Drug-induced headache, not elsewhere classified | antidepressant |
| headache | icd10 | R51 | Headache | antidepressant |
| nausea/vomiting | icd10 | R111 | Nausea alone | antidepressant |
| nausea/vomiting | icd10 | R112 | Vomiting alone | antidepressant |
| nausea/vomiting | icd10 | R113 | Nausea with vomiting | antidepressant |
| poisoning by antidep | icd10 | T430 | Poisoning by tricyclic and tetracyclic antidepressants | antidepressant |
| poisoning by antidep | icd10 | T431 | Poisoning by monoamine-oxidase-inhibitor antidepressants | antidepressant |
| poisoning by antidep | icd10 | T432 | Poisoning by other and unspecified antidepressants | antidepressant |
| sexual dysfunction | icd10 | F520 | Lack or loss of sexual desire | antidepressant |
| sexual dysfunction | icd10 | F521 | Sexual aversion and lack of sexual enjoyment | antidepressant |
| sexual dysfunction | icd10 | F522 | Failure of genital response | antidepressant |
| sexual dysfunction | icd10 | F523 | Orgasmic dysfunction | antidepressant |
| sexual dysfunction | icd10 | F524 | Premature ejaculation | antidepressant |
| sexual dysfunction | icd10 | F528 | Other sexual dysfunction, not caused by organic disorder or disease | antidepressant |
| sexual dysfunction | icd10 | F529 | Unspecified sexual dysfunction, not caused by organic disorder or disease | antidepressant |
| suicidality/self-harm | icd10 | X64 | Intentional self-poisoning by and exposure to other and unspecified drugs, medicaments and biological substances | antidepressant |
| tremor | icd10 | G250 | Essential tremor | antidepressant |
| tremor | icd10 | G251 | Drug-induced tremor | antidepressant |
| upper GI tract bleeding | icd10 | K250 | Gastric ulcer, acute with haemorrhage | antidepressant |
| upper GI tract bleeding | icd10 | K251 | Gastric ulcer, acute with perforation | antidepressant |
| upper GI tract bleeding | icd10 | K252 | Gastric ulcer, acute with both haemorrhage and perforation | antidepressant |
| upper GI tract bleeding | icd10 | K253 | Gastric ulcer, acute without haemorrhage or perforation | antidepressant |
| upper GI tract bleeding | icd10 | K254 | Gastric ulcer, chronic or unspecified with haemorrhage | antidepressant |
| upper GI tract bleeding | icd10 | K255 | Gastric ulcer, chronic or unspecified with perforation | antidepressant |
| upper GI tract bleeding | icd10 | K256 | Gastric ulcer, chronic or unspecified with both haemorrhage and perforation | antidepressant |
| upper GI tract bleeding | icd10 | K257 | Gastric ulcer, chronic without haemorrhage or perforation | antidepressant |
| upper GI tract bleeding | icd10 | K259 | Gastric ulcer, unspecified as acute or chronic, without haemorrhage or perforation | antidepressant |
| upper GI tract bleeding | icd10 | K260 | Duodenal ulcer, acute with haemorrhage | antidepressant |
| upper GI tract bleeding | icd10 | K261 | Duodenal ulcer, acute with perforation | antidepressant |
| upper GI tract bleeding | icd10 | K262 | Duodenal ulcer, acute with both haemorrhage and perforation | antidepressant |
| upper GI tract bleeding | icd10 | K263 | Duodenal ulcer, acute without haemorrhage or perforation | antidepressant |
| upper GI tract bleeding | icd10 | K264 | Duodenal ulcer, chronic or unspecified with haemorrhage | antidepressant |
| upper GI tract bleeding | icd10 | K265 | Duodenal ulcer, chronic or unspecified with perforation | antidepressant |
| upper GI tract bleeding | icd10 | K266 | Duodenal ulcer, chronic or unspecified with both haemorrhage and perforation | antidepressant |
| upper GI tract bleeding | icd10 | K267 | Duodenal ulcer, chronic without haemorrhage or perforation | antidepressant |
| upper GI tract bleeding | icd10 | K269 | Duodenal ulcer, unspecified as acute or chronic, without haemorrhage or perforation | antidepressant |
| upper GI tract bleeding | icd10 | K270 | Peptic ulcer, acute with haemorrhage | antidepressant |
| upper GI tract bleeding | icd10 | K271 | Peptic ulcer, acute with perforation | antidepressant |
| upper GI tract bleeding | icd10 | K272 | Peptic ulcer, acute with both haemorrhage and perforation | antidepressant |
| upper GI tract bleeding | icd10 | K273 | Peptic ulcer, acute without haemorrhage or perforation | antidepressant |
| upper GI tract bleeding | icd10 | K274 | Peptic ulcer, chronic or unspecified with haemorrhage | antidepressant |
| upper GI tract bleeding | icd10 | K275 | Peptic ulcer, chronic or unspecified with perforation | antidepressant |
| upper GI tract bleeding | icd10 | K276 | Peptic ulcer, chronic or unspecified with both haemorrhage and perforation | antidepressant |
| upper GI tract bleeding | icd10 | K277 | Peptic ulcer, chronic without haemorrhage or perforation | antidepressant |
| upper GI tract bleeding | icd10 | K279 | Peptic ulcer, unspecified as acute or chronic, without haemorrhage or perforation | antidepressant |
| upper GI tract bleeding | icd10 | K280 | Gastrojejunal ulcer, acute with haemorrhage | antidepressant |
| upper GI tract bleeding | icd10 | K281 | Gastrojejunal ulcer, acute with perforation | antidepressant |
| upper GI tract bleeding | icd10 | K282 | Gastrojejunal ulcer, acute with both haemorrhage and perforation | antidepressant |
| upper GI tract bleeding | icd10 | K283 | Gastrojejunal ulcer, acute without haemorrhage or perforation | antidepressant |
| upper GI tract bleeding | icd10 | K284 | Gastrojejunal ulcer, chronic or unspecified with haemorrhage | antidepressant |
| upper GI tract bleeding | icd10 | K285 | Gastrojejunal ulcer, chronic or unspecified with perforation | antidepressant |
| upper GI tract bleeding | icd10 | K286 | Gastrojejunal ulcer, chronic or unspecified with both haemorrhage and perforation | antidepressant |
| upper GI tract bleeding | icd10 | K287 | Gastrojejunal ulcer, chronic without haemorrhage or perforation | antidepressant |
| upper GI tract bleeding | icd10 | K289 | Gastrojejunal ulcer, unspecified as acute or chronic, without haemorrhage or perforation | antidepressant |
| upper GI tract bleeding | icd10 | K922 | Gastrointestinal haemorrhage, unspecified | antidepressant |
| AE by antidep | icd9 | E9390 | Antidepressants causing adverse effects in therapeutic use | antidepressant |
| dizziness | icd9 | 7804 | General symptoms, dizziness and giddiness | antidepressant |
| headache | icd9 | 3393 | Drug induced headache, not elsewhere classified | antidepressant |
| headache | icd9 | 7840 | Symptoms involving head and neck, headache | antidepressant |
| nausea/vomiting | icd9 | 7870 | Symptoms involving digestive system, nausea and vomiting | antidepressant |
| poisoning by antidep | icd9 | 9690 | Poisoning by psychotropic agents, antidepressants | antidepressant |
| poisoning by antidep | icd9 | E8540 | Accidental poisoning by antidepressants | antidepressant |
| sexual dysfunction | icd9 | 3027 | Psychosexual dysfunction | antidepressant |
| sexual dysfunction | icd9 | 3029 | Sexual deviations and disorders, unspecified | antidepressant |
| sexual dysfunction | icd9 | 6078 | Disorders of penis, other | antidepressant |
| sexual dysfunction | icd9 | V417 | Problems with sexual function | antidepressant |
| suicidality/self-harm | icd9 | E9500 | Suicide and self-inflicted poisoning by analgesics, antipyretics, and antirheumatics | antidepressant |
| suicidality/self-harm | icd9 | E9501 | Suicide and self-inflicted poisoning by barbiturates | antidepressant |
| suicidality/self-harm | icd9 | E9502 | Suicide and self-inflicted poisoning by other sedatives and hypnotics | antidepressant |
| suicidality/self-harm | icd9 | E9503 | Suicide and self-inflicted poisoning by tranquilizers and other psychotropic agents | antidepressant |
| suicidality/self-harm | icd9 | E9504 | Suicide and self-inflicted poisoning by other specified drugs and medicinal substances | antidepressant |
| suicidality/self-harm | icd9 | E9505 | Suicide and self-inflicted poisoning by unspecified drug or medicinal substance | antidepressant |
| tremor | icd9 | 3331 | Essential and other specified forms of tremor | antidepressant |
| upper GI tract bleeding | icd9 | 5310 | Gastric ulcer, acute with haemorrhage | antidepressant |
| upper GI tract bleeding | icd9 | 5311 | Gastric ulcer, acute with perforation | antidepressant |
| upper GI tract bleeding | icd9 | 5312 | Gastric ulcer, acute with haemorrhage and perforation | antidepressant |
| upper GI tract bleeding | icd9 | 5313 | Gastric ulcer, acute without mention of haemorrhage or perforation | antidepressant |
| upper GI tract bleeding | icd9 | 5314 | Gastric ulcer, chronic or unspecified with haemorrhage | antidepressant |
| upper GI tract bleeding | icd9 | 5315 | Gastric ulcer, chronic or unspecified with perforation | antidepressant |
| upper GI tract bleeding | icd9 | 5316 | Gastric ulcer, chronic or unspecified with haemorrhage and perforation | antidepressant |
| upper GI tract bleeding | icd9 | 5317 | Gastric ulcer, chronic without mention of haemorrhage or perforation | antidepressant |
| upper GI tract bleeding | icd9 | 5319 | Gastric ulcer, without mention of haemorrhage or perforation | antidepressant |
| upper GI tract bleeding | icd9 | 5320 | Duodenal ulcer, acute with haemorrhage | antidepressant |
| upper GI tract bleeding | icd9 | 5321 | Duodenal ulcer, acute with perforation | antidepressant |
| upper GI tract bleeding | icd9 | 5322 | Duodenal ulcer, acute with haemorrhage and perforation | antidepressant |
| upper GI tract bleeding | icd9 | 5323 | Duodenal ulcer, acute without mention of haemorrhage or perforation | antidepressant |
| upper GI tract bleeding | icd9 | 5324 | Duodenal ulcer, chronic or unspecified with haemorrhage | antidepressant |
| upper GI tract bleeding | icd9 | 5325 | Duodenal ulcer, chronic or unspecified with perforation | antidepressant |
| upper GI tract bleeding | icd9 | 5326 | Duodenal ulcer, chronic or unspecified with haemorrhage and perforation | antidepressant |
| upper GI tract bleeding | icd9 | 5327 | Duodenal ulcer, chronic without mention of haemorrhage or perforation | antidepressant |
| upper GI tract bleeding | icd9 | 5329 | Duodenal ulcer unspecified, without mention of haemorrhage or perforation | antidepressant |
| upper GI tract bleeding | icd9 | 5330 | Peptic ulcer, site unspecified, acute with haemorrhage | antidepressant |
| upper GI tract bleeding | icd9 | 5331 | Peptic ulcer, site unspecified, acute with perforation | antidepressant |
| upper GI tract bleeding | icd9 | 5332 | Peptic ulcer, site unspecified, acute with haemorrhage and perforation | antidepressant |
| upper GI tract bleeding | icd9 | 5333 | Acute without mention of haemorrhage or perforation | antidepressant |
| upper GI tract bleeding | icd9 | 5334 | Peptic ulcer, site unspecified, chronic or unspecified with haemorrhage | antidepressant |
| upper GI tract bleeding | icd9 | 5335 | Peptic ulcer, site unspecified, chronic or unspecified with perforation | antidepressant |
| upper GI tract bleeding | icd9 | 5336 | Chronic or unspecified with haemorrhage and perforation | antidepressant |
| upper GI tract bleeding | icd9 | 5337 | Chronic without mention of haemorrhage or perforation | antidepressant |
| upper GI tract bleeding | icd9 | 5339 | Peptic ulcer, unspecified, without mention of haemorrhage or perforation | antidepressant |
| upper GI tract bleeding | icd9 | 5340 | Gastrojejunal ulcer, acute with haemorrhage | antidepressant |
| upper GI tract bleeding | icd9 | 5341 | Gastrojejunal ulcer, acute with perforation | antidepressant |
| upper GI tract bleeding | icd9 | 5342 | Gastrojejunal ulcer, acute with haemorrhage and perforation | antidepressant |
| upper GI tract bleeding | icd9 | 5343 | Gastrojejunal ulcer, acute without mention of haemorrhage or perforation | antidepressant |
| upper GI tract bleeding | icd9 | 5344 | Gastrojejunal ulcer, chronic or unspecified with haemorrhage | antidepressant |
| upper GI tract bleeding | icd9 | 5345 | Gastrojejunal ulcer, chronic or unspecified with perforation | antidepressant |
| upper GI tract bleeding | icd9 | 5346 | Chronic or unspecified with haemorrhage and perforation | antidepressant |
| upper GI tract bleeding | icd9 | 5347 | Gastrojejunal ulcer, chronic without mention of haemorrhage or perforation | antidepressant |
| upper GI tract bleeding | icd9 | 5349 | Gastrojejunal ulcer, without mention of haemorrhage or perforation | antidepressant |
| upper GI tract bleeding | icd9 | 5789 | Haemorrhage of gastrointestinal tract, unspecified | antidepressant |
| AE by antihyp | icd10 | Y514 | Predominantly alpha-adrenoreceptor agonists, not elsewhere classified, causing adverse effect in therapeutic use | antihypertensive |
| AE by antihyp | icd10 | Y516 | alpha-Adrenoreceptor antagonists, not elsewhere classified, causing adverse effect in therapeutic use | antihypertensive |
| AE by antihyp | icd10 | Y517 | beta-Adrenoreceptor antagonists, not elsewhere classified, causing adverse effect in therapeutic use | antihypertensive |
| AE by antihyp | icd10 | Y518 | Centrally acting and adrenergic-neuron-blocking agents, not elsewhere classified, causing adverse effect in therapeutic use | antihypertensive |
| AE by antihyp | icd10 | Y521 | Calcium-channel blockers causing adverse effect in therapeutic use | antihypertensive |
| AE by antihyp | icd10 | Y523 | Coronary vasodilators, not elsewhere classified, causing adverse effect in therapeutic use | antihypertensive |
| AE by antihyp | icd10 | Y524 | Angiotensin-converting-enzyme inhibitors causing adverse effect in therapeutic use | antihypertensive |
| AE by antihyp | icd10 | Y525 | Other antihypertensive drugs, not elsewhere classified, causing adverse effect in therapeutic use | antihypertensive |
| AE by antihyp | icd10 | Y527 | Peripheral vasodilators causing adverse effect in therapeutic use | antihypertensive |
| AE by antihyp | icd10 | Y541 | Mineralocorticoid antagonists [aldosterone antagonists] causing adverse effect in therapeutic use | antihypertensive |
| AE by antihyp | icd10 | Y542 | Carbonic-anhydrase inhibitors causing adverse effect in therapeutic use | antihypertensive |
| AE by antihyp | icd10 | Y544 | Loop [high-ceiling] diuretics causing adverse effect in therapeutic use | antihypertensive |
| AE by antihyp | icd10 | Y545 | Other diuretics causing adverse effect in therapeutic use | antihypertensive |
| angioedema | icd10 | T783 | Angioneurotic oedema | antihypertensive |
| bradycardia | icd10 | I440 | Atrioventricular block, first degree | antihypertensive |
| bradycardia | icd10 | I441 | Atrioventricular block, second degree | antihypertensive |
| bradycardia | icd10 | I442 | Atrioventricular block, complete | antihypertensive |
| bradycardia | icd10 | I443 | Other and unspecified atrioventricular block | antihypertensive |
| bradycardia | icd10 | I444 | Left anterior fascicular block | antihypertensive |
| bradycardia | icd10 | I445 | Left posterior fascicular block | antihypertensive |
| bradycardia | icd10 | I446 | Other and unspecified fascicular block | antihypertensive |
| bradycardia | icd10 | I447 | Left bundle-branch block, unspecified | antihypertensive |
| bradycardia | icd10 | I458 | Other specified conduction disorders | antihypertensive |
| bradycardia | icd10 | R001 | Bradycardia, unspecified | antihypertensive |
| dizziness/vertigo | icd10 | R42 | Dizziness and giddiness | antihypertensive |
| dyspnea | icd10 | R060 | Dyspnoea | antihypertensive |
| headache | icd10 | G444 | Drug-induced headache, not elsewhere classified | antihypertensive |
| headache | icd10 | R51 | Headache | antihypertensive |
| hepatotoxicity | icd10 | K710 | Toxic liver disease with cholestasis | antihypertensive |
| hepatotoxicity | icd10 | K711 | Toxic liver disease with hepatic necrosis | antihypertensive |
| hepatotoxicity | icd10 | K712 | Toxic liver disease with acute hepatitis | antihypertensive |
| hepatotoxicity | icd10 | K713 | Toxic liver disease with chronic persistent hepatitis | antihypertensive |
| hepatotoxicity | icd10 | K714 | Toxic liver disease with chronic lobular hepatitis | antihypertensive |
| hepatotoxicity | icd10 | K715 | Toxic liver disease with chronic active hepatitis | antihypertensive |
| hepatotoxicity | icd10 | K716 | Toxic liver disease with hepatitis, not elsewhere classified | antihypertensive |
| hepatotoxicity | icd10 | K717 | Toxic liver disease with fibrosis and cirrhosis of liver | antihypertensive |
| hepatotoxicity | icd10 | K718 | Toxic liver disease with other disorders of liver | antihypertensive |
| hepatotoxicity | icd10 | K719 | Toxic liver disease, unspecified | antihypertensive |
| hepatotoxicity | icd10 | K720 | Acute and subacute hepatic failure | antihypertensive |
| hyperglycemia | icd10 | R739 | Hyperglycaemia, unspecified | antihypertensive |
| hyperkalemia | icd10 | E875 | Hyperkalaemia | antihypertensive |
| hypokalemia | icd10 | E876 | Hypokalaemia | antihypertensive |
| hyponatremia | icd10 | E871 | Hypo-osmolality and hyponatraemia | antihypertensive |
| hypotension | icd10 | I952 | Hypotension due to drugs | antihypertensive |
| hypovolemia | icd10 | E86 | Volume depletion | antihypertensive |
| metabolic alkalosis | icd10 | E873 | Alkalosis | antihypertensive |
| nausea | icd10 | R111 | Nausea alone | antihypertensive |
| nausea | icd10 | R113 | Nausea with vomiting | antihypertensive |
| photosensitivity dermatitis | icd10 | L560 | Drug phototoxic response | antihypertensive |
| photosensitivity dermatitis | icd10 | L561 | Drug photoallergic response | antihypertensive |
| poisoning by antihyp | icd10 | T444 | Poisoning by predominantly alpha-adrenoreceptor agonists, not elsewhere classified | antihypertensive |
| poisoning by antihyp | icd10 | T446 | Poisoning by alpha-adrenoreceptor antagonists, not elsewhere classified | antihypertensive |
| poisoning by antihyp | icd10 | T447 | Poisoning by beta-Adrenoreceptor antagonists, not elsewhere classified | antihypertensive |
| poisoning by antihyp | icd10 | T448 | Poisoning by centrally acting and adrenergic-neuron-blocking agents, not elsewhere classified | antihypertensive |
| poisoning by antihyp | icd10 | T461 | Poisoning by calcium-channel blockers | antihypertensive |
| poisoning by antihyp | icd10 | T463 | Poisoning by coronary vasodilators, not elsewhere classified | antihypertensive |
| poisoning by antihyp | icd10 | T464 | Poisoning by angiotensin-converting-enzyme inhibitors | antihypertensive |
| poisoning by antihyp | icd10 | T465 | Poisoning by other antihypertensive drugs, not elsewhere classified | antihypertensive |
| poisoning by antihyp | icd10 | T467 | Poisoning by peripheral vasodilators | antihypertensive |
| poisoning by antihyp | icd10 | T501 | Poisoning by loop [high-ceiling] diuretics | antihypertensive |
| poisoning by antihyp | icd10 | T502 | Poisoning by carbonic-anhydrase inhibitors, benzothiadiazides and other diuretics | antihypertensive |
| rash | icd10 | L270 | Generalized skin eruption due to drugs and medicaments | antihypertensive |
| rash | icd10 | L271 | Localized skin eruption due to drugs and medicaments | antihypertensive |
| rash | icd10 | L278 | Dermatitis due to other substances taken internally | antihypertensive |
| rash | icd10 | L279 | Dermatitis due to unspecified substance taken internally | antihypertensive |
| rash | icd10 | R21 | Rash and other nonspecific skin eruption | antihypertensive |
| renal impairment | icd10 | N170 | Acute renal failure with tubular necrosis | antihypertensive |
| renal impairment | icd10 | N171 | Acute renal failure with acute cortical necrosis | antihypertensive |
| renal impairment | icd10 | N172 | Acute renal failure with medullary necrosis | antihypertensive |
| renal impairment | icd10 | N178 | Other acute renal failure | antihypertensive |
| renal impairment | icd10 | N179 | Acute renal failure, unspecified | antihypertensive |
| renal impairment | icd10 | N19 | Unspecified renal failure | antihypertensive |
| sexual dysfunction | icd10 | F520 | Lack or loss of sexual desire | antihypertensive |
| sexual dysfunction | icd10 | F521 | Sexual aversion and lack of sexual enjoyment | antihypertensive |
| sexual dysfunction | icd10 | F522 | Failure of genital response | antihypertensive |
| sexual dysfunction | icd10 | F523 | Orgasmic dysfunction | antihypertensive |
| sexual dysfunction | icd10 | F524 | Premature ejaculation | antihypertensive |
| sexual dysfunction | icd10 | F528 | Other sexual dysfunction, not caused by organic disorder or disease | antihypertensive |
| sexual dysfunction | icd10 | F529 | Unspecified sexual dysfunction, not caused by organic disorder or disease | antihypertensive |
| AE by antihyp | icd9 | E9413 | Sympatholytics [antiadrenergics] causing adverse effects in therapeutic use | antihypertensive |
| AE by antihyp | icd9 | E9424 | Coronary vasodilators causing adverse effects in therapeutic use | antihypertensive |
| AE by antihyp | icd9 | E9425 | Other vasodilators causing adverse effects in therapeutic use | antihypertensive |
| AE by antihyp | icd9 | E9426 | Other antihypertensive agents causing adverse effects in therapeutic use | antihypertensive |
| AE by antihyp | icd9 | E9440 | Mercurial diuretics causing adverse effects in therapeutic use | antihypertensive |
| AE by antihyp | icd9 | E9441 | Purine derivative diuretics causing adverse effects in therapeutic use | antihypertensive |
| AE by antihyp | icd9 | E9442 | Carbonic acid anhydrase inhibitors causing adverse effects in therapeutic use | antihypertensive |
| AE by antihyp | icd9 | E9443 | Saluretics causing adverse effects in therapeutic use | antihypertensive |
| AE by antihyp | icd9 | E9444 | Other diuretics causing adverse effects in therapeutic use | antihypertensive |
| angioedema | icd9 | 9951 | Angioneurotic edema, not elsewhere classified | antihypertensive |
| bradycardia | icd9 | 4260 | Conduction disorders, atrioventricular block, complete | antihypertensive |
| bradycardia | icd9 | 4262 | Conduction disorders, left bundle branch hemiblock | antihypertensive |
| bradycardia | icd9 | 4264 | Conduction disorders, right bundle branch block | antihypertensive |
| bradycardia | icd9 | 4265 | Conduction disorders, bundle branch block, unspecified | antihypertensive |
| bradycardia | icd9 | 4268 | Conduction disorders, other | antihypertensive |
| bradycardia | icd9 | 4269 | Conduction disorders, unspecified | antihypertensive |
| dizziness/vertigo | icd9 | 7804 | General symptoms, dizziness and giddiness | antihypertensive |
| dyspnea | icd9 | 7860 | Dyspnée et anomalies respiratoires | antihypertensive |
| headache | icd9 | 3393 | Drug induced headache, not elsewhere classified | antihypertensive |
| headache | icd9 | 7840 | Symptoms involving head and neck, headache | antihypertensive |
| hepatotoxicity | icd9 | 5733 | Hepatitis, unspecified | antihypertensive |
| hyperkalemia | icd9 | 2767 | Hyperpotassemia | antihypertensive |
| hypokalemia | icd9 | 2768 | Hypopotassemia | antihypertensive |
| hyponatremia | icd9 | 2761 | Hyposmolality and/or hyponatremia | antihypertensive |
| hypotension | icd9 | 45829 | Other iatrogenic hypotension | antihypertensive |
| metabolic alkalosis | icd9 | 2763 | Disorders of fluid, electrolyte and acid-base balance, alkalosis | antihypertensive |
| nausea | icd9 | 7870 | Symptoms involving digestive system, nausea and vomiting | antihypertensive |
| photosensitivity dermatitis | icd9 | 6927 | Contact dermatitis and other eczema, due to solar radiation | antihypertensive |
| poisoning by antihyp | icd9 | 9713 | Sympatholytics (antiadrenergics) | antihypertensive |
| poisoning by antihyp | icd9 | 9720 | Cardiac rhythm regulators | antihypertensive |
| poisoning by antihyp | icd9 | 9724 | Coronary vasodilators | antihypertensive |
| poisoning by antihyp | icd9 | 9725 | Other vasodilators | antihypertensive |
| poisoning by antihyp | icd9 | 9726 | Other antihypertensive agents | antihypertensive |
| poisoning by antihyp | icd9 | 9729 | Other and unspecified | antihypertensive |
| poisoning by antihyp | icd9 | 9742 | Carbonic acid anhydrase inhibitors | antihypertensive |
| poisoning by antihyp | icd9 | 9744 | Poisoning by water, mineral and uric acid metabolism drugs, other diuretics | antihypertensive |
| poisoning by antihyp | icd9 | E8556 | Accidental poisoning by sympatholytics [antiadrenergics] | antihypertensive |
| poisoning by antihyp | icd9 | E8585 | Accidental poisoning by water, mineral, and uric acid metabolism drugs | antihypertensive |
| rash | icd9 | 6930 | Dermatitis due to substances taken internally, due to drugs and medicaments | antihypertensive |
| rash | icd9 | 6938 | Due to other specified substance | antihypertensive |
| rash | icd9 | 6939 | Dermatitis due to substances taken internally, due to unspecified substance | antihypertensive |
| rash | icd9 | 7821 | Rash and other nonspecific skin eruption | antihypertensive |
| renal impairment | icd9 | 5845 | Acute renal failure, with lesion of tubular necrosis | antihypertensive |
| renal impairment | icd9 | 5846 | Acute renal failure, with lesion of renal cortical necrosis | antihypertensive |
| renal impairment | icd9 | 5847 | Acute renal failure, with lesion of renal medullary (papillary) necrosis | antihypertensive |
| renal impairment | icd9 | 5848 | Acute renal failure, with other specified pathological lesion in kidney | antihypertensive |
| renal impairment | icd9 | 5849 | Acute renal failure, unspecified | antihypertensive |
| renal impairment | icd9 | 5869 | Renal failure, unspecified | antihypertensive |
| sexual dysfunction | icd9 | 3027 | Psychosexual dysfunction | antihypertensive |
| sexual dysfunction | icd9 | 3029 | Sexual deviations and disorders, unspecified | antihypertensive |
| sexual dysfunction | icd9 | 6078 | Disorders of penis, other | antihypertensive |
| sexual dysfunction | icd9 | V417 | Problems with sexual function | antihypertensive |

**Appendix 4. Characteristics of consented patients, patients who declined consent, patients who were not dispensed the study drug, patients who were dispensed the study drug, and patients who were included in the final analysis.**

|  | **Consented (N=1,271)** | **Declined Consent (N=1,393)** | **Enrolled, drug not dispensed (N=468)** | **Enrolled, drug dispensed (N=803)** | **Enrolled, drug dispensed, interviewed (N=688)** |
| --- | --- | --- | --- | --- | --- |
| **Sex (male)** | 473 (37.2%) | 477 (34.2%) | 190 (40.6%) | 283 (35.2%) | 243 (35.3%) |
| **Age, mean (SD)** | 64.1 (14.1) | 67.0 (14.0) | 64.5 (13.6) | 63.8 (14.4) | 64.2 (13.9) |
| **Study Medication** |  |  |  |  |  |
| Antihypertensive | 742 (58.4%) | 824 (59.2%) | 298 (63.7%) | 444 (55.3%) | 386 (56.1%) |
| Antidepressant | 529 (41.6%) | 569 (40.9%) | 170 (36.3%) | 359 (44.7%) | 302 (43.9%) |
| **Top indications for patients prescribed an antihypertive** | N=742 | N=824 | N=298 | N=444 | N=386 |
| Hypertension | 608 (81.9%) | 646 (78.4%) | 258 (86.6%) | 350 (78.8%) | 306 (79.3%) |
| Oedema | 26 (3.5%) | 43 (5.2%) | 7 (2.4%) | 19 (4.3%) | 18 (4.7%) |
| **Top indications for patients prescribed an antidepressant** | N=529 | N=569 | N=170 | N=359 | N=302 |
| Depression | 214 (40.5%) | 254 (44.6%) | 63 (37.1%) | 151 (42.1%) | 121 (40.1%) |
| Anxiety | 107 (20.2%) | 113 (19.9%) | 37 (21.8%) | 70 (19.5%) | 62 (20.5%) |
| Insomnia | 76 (14.4%) | 94 (16.5%) | 20 (11.8%) | 56 (15.6%) | 52 (17.2%) |

# Appendix 5. Symptoms reported during interview, overall and by study drug therapeutic class, and among patients who reported all, none, or some of their symptoms to their physician.

| **Symptom** | **All Patients Reporting Symptoms N=318** | | | | | | | | **Patients on Antidepressant N=168** | | | | | | | | **Patients on Antihypertensive N=150** | | | | | | | |
| --- | --- | --- | --- | --- | --- | --- | --- | --- | --- | --- | --- | --- | --- | --- | --- | --- | --- | --- | --- | --- | --- | --- | --- | --- |
|  | **Pts who reported none to MD N=79** | | **Pts who reported all to MD N=233** | | **Pts who reported some to MD N=6** | | **OVERALL N=318** | | **Pts who reported none to MD N=38** | | **Pts who reported all to MD N=125** | | **Pts who reported some to MD N=5** | | **OVERALL N=168** | | **Pts who reported none to MD N=41** | | **Pts who reported all to MD N=108** | | **Pts who reported some to MD N=1** | | **OVERALL N=150** | |
|  | **N** | **%** | **N** | **%** | **N** | **%** | **N** | **%** | **N** | **%** | **N** | **%** | **N** | **%** | **N** | **%** | **N** | **%** | **N** | **%** | **N** | **%** | **N** | **%** |
| headache | 38 | 16.3 | 16 | 20.3 | 3 | 50.0 | **57** | **17.9** | 20 | 16.0 | 8 | 21.1 | 3 | 60.0 | **31** | **18.5** | 18 | 16.7 | 8 | 19.5 | 0 | 0 | **26** | **17.3** |
| dizziness or staggering (vertigo) | 35 | 15.0 | 13 | 16.5 | 3 | 50.0 | **51** | **16.0** | 14 | 11.2 | 8 | 21.1 | 3 | 60.0 | **25** | **14.9** | 21 | 19.4 | 5 | 12.2 | 0 | 0 | **26** | **17.3** |
| dry mouth or throat | 36 | 15.5 | 8 | 10.1 | 4 | 66.7 | **48** | **15.1** | 26 | 20.8 | 5 | 13.2 | 3 | 60.0 | **34** | **20.2** | 10 | 9.3 | 3 | 7.3 | 1 | 100 | **14** | **9.3** |
| nausea or vomiting | 35 | 15.0 | 8 | 10.1 | 2 | 33.3 | **45** | **14.2** | 24 | 19.2 | 4 | 10.5 | 2 | 40.0 | **30** | **17.9** | 11 | 10.2 | 4 | 9.8 | 0 | 0 | **15** | **10.0** |
| unusual tiredness or weakness | 29 | 12.4 | 7 | 8.9 | 0 | 0.0 | **36** | **11.3** | 10 | 8.0 | 2 | 5.3 | 0 | 0.0 | **12** | **7.1** | 19 | 17.6 | 5 | 12.2 | 0 | 0 | **24** | **16.0** |
| light-headed when getting up from a lying or sitting position or feeling faint | 29 | 12.4 | 5 | 6.3 | 1 | 16.7 | **35** | **11.0** | 18 | 14.4 | 2 | 5.3 | 1 | 20.0 | **21** | **12.5** | 11 | 10.2 | 3 | 7.3 | 0 | 0 | **14** | **9.3** |
| increased sleep or drowsiness | 23 | 9.9 | 8 | 10.1 | 0 | 0.0 | **31** | **9.7** | 19 | 15.2 | 7 | 18.4 | 0 | 0.0 | **26** | **15.5** | 4 | 3.7 | 1 | 2.4 | 0 | 0 | **5** | **3.3** |
| constipation | 17 | 7.3 | 3 | 3.8 | 2 | 33.3 | **22** | **6.9** | 6 | 4.8 | 1 | 2.6 | 2 | 40.0 | **9** | **5.4** | 11 | 10.2 | 2 | 4.9 | 0 | 0 | **13** | **8.7** |
| diarrhoea | 15 | 6.4 | 5 | 6.3 | 0 | 0.0 | **20** | **6.3** | 10 | 8.0 | 4 | 10.5 | 0 | 0.0 | **14** | **8.3** | 5 | 4.6 | 1 | 2.4 | 0 | 0 | **6** | **4.0** |
| reduction in sleeping | 17 | 7.3 | 2 | 2.5 | 1 | 16.7 | **20** | **6.3** | 15 | 12.0 | 1 | 2.6 | 1 | 20.0 | **17** | **10.1** | 2 | 1.9 | 1 | 2.4 | 0 | 0 | **3** | **2.0** |
| decrease in appetite | 10 | 4.3 | 3 | 3.8 | 2 | 33.3 | **15** | **4.7** | 9 | 7.2 | 2 | 5.3 | 2 | 40.0 | **13** | **7.7** | 1 | 0.9 | 1 | 2.4 | 0 | 0 | **2** | **1.3** |
| flushing of skin/hot flush | 11 | 4.7 | 4 | 5.1 | 0 | 0.0 | **15** | **4.7** | 4 | 3.2 | 1 | 2.6 | 0 | 0.0 | **5** | **3.0** | 7 | 6.5 | 3 | 7.3 | 0 | 0 | **10** | **6.7** |
| anxiety (nervousness) or agitation | 12 | 5.2 | 1 | 1.3 | 2 | 33.3 | **15** | **4.7** | 11 | 8.8 | 1 | 2.6 | 2 | 40.0 | **14** | **8.3** | 1 | 0.9 | 0 | 0.0 | 0 | 0 | **1** | **0.7** |
| indigestion or heartburn | 10 | 4.3 | 4 | 5.1 | 0 | 0.0 | **14** | **4.4** | 6 | 4.8 | 3 | 7.9 | 0 | 0.0 | **9** | **5.4** | 4 | 3.7 | 1 | 2.4 | 0 | 0 | **5** | **3.3** |
| passing water more often | 10 | 4.3 | 4 | 5.1 | 0 | 0.0 | **14** | **4.4** | 2 | 1.6 | 1 | 2.6 | 0 | 0.0 | **3** | **1.8** | 8 | 7.4 | 3 | 7.3 | 0 | 0 | **11** | **7.3** |
| muscle pain | 10 | 4.3 | 3 | 3.8 | 0 | 0.0 | **13** | **4.1** | 2 | 1.6 | 1 | 2.6 | 0 | 0.0 | **3** | **1.8** | 8 | 7.4 | 2 | 4.9 | 0 | 0 | **10** | **6.7** |
| palpitations/racing heart | 7 | 3.0 | 6 | 7.6 | 0 | 0.0 | **13** | **4.1** | 4 | 3.2 | 2 | 5.3 | 0 | 0.0 | **6** | **3.6** | 3 | 2.8 | 4 | 9.8 | 0 | 0 | **7** | **4.7** |
| swelling of feet | 6 | 2.6 | 6 | 7.6 | 0 | 0.0 | **12** | **3.8** | 1 | 0.8 | 0 | 0.0 | 0 | 0.0 | **1** | **0.6** | 5 | 4.6 | 6 | 14.6 | 0 | 0 | **11** | **7.3** |
| cough | 6 | 2.6 | 2 | 2.5 | 0 | 0.0 | **8** | **2.5** | 3 | 2.4 | 0 | 0.0 | 0 | 0.0 | **3** | **1.8** | 3 | 2.8 | 2 | 4.9 | 0 | 0 | **5** | **3.3** |
| pain or cramps in lower abdomen | 5 | 2.1 | 1 | 1.3 | 0 | 0.0 | **6** | **1.9** | 2 | 1.6 | 1 | 2.6 | 0 | 0.0 | **3** | **1.8** | 3 | 2.8 | 0 | 0.0 | 0 | 0 | **3** | **2.0** |
| chest pressure/pain | 4 | 1.7 | 1 | 1.3 | 1 | 16.7 | **6** | **1.9** | 4 | 3.2 | 1 | 2.6 | 1 | 20.0 | **6** | **3.6** | 0 | 0.0 | 0 | 0.0 | 0 | 0 | **0** | **0.0** |
| drop in blood pressure | 3 | 1.3 | 3 | 3.8 | 0 | 0.0 | **6** | **1.9** | 0 | 0.0 | 0 | 0.0 | 0 | 0.0 | **0** | **0.0** | 3 | 2.8 | 3 | 7.3 | 0 | 0 | **6** | **4.0** |
| bloated feeling or gas | 5 | 2.1 | 0 | 0.0 | 0 | 0.0 | **5** | **1.6** | 3 | 2.4 | 0 | 0.0 | 0 | 0.0 | **3** | **1.8** | 2 | 1.9 | 0 | 0.0 | 0 | 0 | **2** | **1.3** |
| itching of skin | 2 | 0.9 | 2 | 2.5 | 1 | 16.7 | **5** | **1.6** | 0 | 0.0 | 2 | 5.3 | 1 | 20.0 | **3** | **1.8** | 2 | 1.9 | 0 | 0.0 | 0 | 0 | **2** | **1.3** |
| passing water less often | 4 | 1.7 | 0 | 0.0 | 1 | 16.7 | **5** | **1.6** | 0 | 0.0 | 0 | 0.0 | 1 | 20.0 | **1** | **0.6** | 4 | 3.7 | 0 | 0.0 | 0 | 0 | **4** | **2.7** |
| runny or stuffy nose | 4 | 1.7 | 1 | 1.3 | 0 | 0.0 | **5** | **1.6** | 3 | 2.4 | 0 | 0.0 | 0 | 0.0 | **3** | **1.8** | 1 | 0.9 | 1 | 2.4 | 0 | 0 | **2** | **1.3** |
| blurred or affected vision | 4 | 1.7 | 1 | 1.3 | 0 | 0.0 | **5** | **1.6** | 3 | 2.4 | 1 | 2.6 | 0 | 0.0 | **4** | **2.4** | 1 | 0.9 | 0 | 0.0 | 0 | 0 | **1** | **0.7** |
| excessive thirst | 4 | 1.7 | 0 | 0.0 | 0 | 0.0 | **4** | **1.3** | 3 | 2.4 | 0 | 0.0 | 0 | 0.0 | **3** | **1.8** | 1 | 0.9 | 0 | 0.0 | 0 | 0 | **1** | **0.7** |
| increase in appetite | 4 | 1.7 | 0 | 0.0 | 0 | 0.0 | **4** | **1.3** | 3 | 2.4 | 0 | 0.0 | 0 | 0.0 | **3** | **1.8** | 1 | 0.9 | 0 | 0.0 | 0 | 0 | **1** | **0.7** |
| weight gain | 3 | 1.3 | 1 | 1.3 | 0 | 0.0 | **4** | **1.3** | 3 | 2.4 | 1 | 2.6 | 0 | 0.0 | **4** | **2.4** | 0 | 0.0 | 0 | 0.0 | 0 | 0 | **0** | **0.0** |
| flu or cold-like symptoms | 3 | 1.3 | 1 | 1.3 | 0 | 0.0 | **4** | **1.3** | 2 | 1.6 | 0 | 0.0 | 0 | 0.0 | **2** | **1.2** | 1 | 0.9 | 1 | 2.4 | 0 | 0 | **2** | **1.3** |
| foot or leg cramps/pain | 3 | 1.3 | 1 | 1.3 | 0 | 0.0 | **4** | **1.3** | 1 | 0.8 | 0 | 0.0 | 0 | 0.0 | **1** | **0.6** | 2 | 1.9 | 1 | 2.4 | 0 | 0 | **3** | **2.0** |
| increased blood pressure | 2 | 0.9 | 2 | 2.5 | 0 | 0.0 | **4** | **1.3** | 0 | 0.0 | 0 | 0.0 | 0 | 0.0 | **0** | **0.0** | 2 | 1.9 | 2 | 4.9 | 0 | 0 | **4** | **2.7** |
| itchy or irritated or inflamed eyes or eyelids | 3 | 1.3 | 0 | 0.0 | 1 | 16.7 | **4** | **1.3** | 3 | 2.4 | 0 | 0.0 | 1 | 20.0 | **4** | **2.4** | 0 | 0.0 | 0 | 0.0 | 0 | 0 | **0** | **0.0** |
| malaise | 2 | 0.9 | 2 | 2.5 | 0 | 0.0 | **4** | **1.3** | 1 | 0.8 | 1 | 2.6 | 0 | 0.0 | **2** | **1.2** | 1 | 0.9 | 1 | 2.4 | 0 | 0 | **2** | **1.3** |
| trembling & shaking of fingers, hands or legs | 2 | 0.9 | 2 | 2.5 | 0 | 0.0 | **4** | **1.3** | 2 | 1.6 | 1 | 2.6 | 0 | 0.0 | **3** | **1.8** | 0 | 0.0 | 1 | 2.4 | 0 | 0 | **1** | **0.7** |
| bone or joint pain | 3 | 1.3 | 0 | 0.0 | 0 | 0.0 | **3** | **0.9** | 1 | 0.8 | 0 | 0.0 | 0 | 0.0 | **1** | **0.6** | 2 | 1.9 | 0 | 0.0 | 0 | 0 | **2** | **1.3** |
| decrease in sexual desire | 2 | 0.9 | 0 | 0.0 | 1 | 16.7 | **3** | **0.9** | 1 | 0.8 | 0 | 0.0 | 1 | 20.0 | **2** | **1.2** | 1 | 0.9 | 0 | 0.0 | 0 | 0 | **1** | **0.7** |
| difficulty breathing | 1 | 0.4 | 1 | 1.3 | 1 | 16.7 | **3** | **0.9** | 1 | 0.8 | 1 | 2.6 | 1 | 20.0 | **3** | **1.8** | 0 | 0.0 | 0 | 0.0 | 0 | 0 | **0** | **0.0** |
| change in fingernails | 3 | 1.3 | 0 | 0.0 | 0 | 0.0 | **3** | **0.9** | 2 | 1.6 | 0 | 0.0 | 0 | 0.0 | **2** | **1.2** | 1 | 0.9 | 0 | 0.0 | 0 | 0 | **1** | **0.7** |
| depression | 0 | 0.0 | 3 | 3.8 | 0 | 0.0 | **3** | **0.9** | 0 | 0.0 | 2 | 5.3 | 0 | 0.0 | **2** | **1.2** | 0 | 0.0 | 1 | 2.4 | 0 | 0 | **1** | **0.7** |
| loss of memory | 3 | 1.3 | 0 | 0.0 | 0 | 0.0 | **3** | **0.9** | 3 | 2.4 | 0 | 0.0 | 0 | 0.0 | **3** | **1.8** | 0 | 0.0 | 0 | 0.0 | 0 | 0 | **0** | **0.0** |
| nightmares | 2 | 0.9 | 1 | 1.3 | 0 | 0.0 | **3** | **0.9** | 2 | 1.6 | 1 | 2.6 | 0 | 0.0 | **3** | **1.8** | 0 | 0.0 | 0 | 0.0 | 0 | 0 | **0** | **0.0** |
| ringing, buzzing or noises in ears | 2 | 0.9 | 0 | 0.0 | 1 | 16.7 | **3** | **0.9** | 2 | 1.6 | 0 | 0.0 | 1 | 20.0 | **3** | **1.8** | 0 | 0.0 | 0 | 0.0 | 0 | 0 | **0** | **0.0** |
| difficulty in passing water | 1 | 0.4 | 1 | 1.3 | 0 | 0.0 | **2** | **0.6** | 0 | 0.0 | 1 | 2.6 | 0 | 0.0 | **1** | **0.6** | 1 | 0.9 | 0 | 0.0 | 0 | 0 | **1** | **0.7** |
| increased sweating | 2 | 0.9 | 0 | 0.0 | 0 | 0.0 | **2** | **0.6** | 1 | 0.8 | 0 | 0.0 | 0 | 0.0 | **1** | **0.6** | 1 | 0.9 | 0 | 0.0 | 0 | 0 | **1** | **0.7** |
| change in mood | 1 | 0.4 | 1 | 1.3 | 0 | 0.0 | **2** | **0.6** | 0 | 0.0 | 1 | 2.6 | 0 | 0.0 | **1** | **0.6** | 1 | 0.9 | 0 | 0.0 | 0 | 0 | **1** | **0.7** |
| difficulty concentrating or learning | 2 | 0.9 | 0 | 0.0 | 0 | 0.0 | **2** | **0.6** | 2 | 1.6 | 0 | 0.0 | 0 | 0.0 | **2** | **1.2** | 0 | 0.0 | 0 | 0.0 | 0 | 0 | **0** | **0.0** |
| fluctuations in blood pressure | 1 | 0.4 | 1 | 1.3 | 0 | 0.0 | **2** | **0.6** | 0 | 0.0 | 0 | 0.0 | 0 | 0.0 | **0** | **0.0** | 1 | 0.9 | 1 | 2.4 | 0 | 0 | **2** | **1.3** |
| migraine headache | 2 | 0.9 | 0 | 0.0 | 0 | 0.0 | **2** | **0.6** | 0 | 0.0 | 0 | 0.0 | 0 | 0.0 | **0** | **0.0** | 2 | 1.9 | 0 | 0.0 | 0 | 0 | **2** | **1.3** |
| mouth soreness or irritation | 2 | 0.9 | 0 | 0.0 | 0 | 0.0 | **2** | **0.6** | 2 | 1.6 | 0 | 0.0 | 0 | 0.0 | **2** | **1.2** | 0 | 0.0 | 0 | 0.0 | 0 | 0 | **0** | **0.0** |
| skin sensitivity | 2 | 0.9 | 0 | 0.0 | 0 | 0.0 | **2** | **0.6** | 2 | 1.6 | 0 | 0.0 | 0 | 0.0 | **2** | **1.2** | 0 | 0.0 | 0 | 0.0 | 0 | 0 | **0** | **0.0** |
| urinary incontinence | 1 | 0.4 | 1 | 1.3 | 0 | 0.0 | **2** | **0.6** | 0 | 0.0 | 1 | 2.6 | 0 | 0.0 | **1** | **0.6** | 1 | 0.9 | 0 | 0.0 | 0 | 0 | **1** | **0.7** |
| decrease in sexual ability | 1 | 0.4 | 0 | 0.0 | 0 | 0.0 | **1** | **0.3** | 0 | 0.0 | 0 | 0.0 | 0 | 0.0 | **0** | **0.0** | 1 | 0.9 | 0 | 0.0 | 0 | 0 | **1** | **0.7** |
| muscle weakness | 0 | 0.0 | 1 | 1.3 | 0 | 0.0 | **1** | **0.3** | 0 | 0.0 | 1 | 2.6 | 0 | 0.0 | **1** | **0.6** | 0 | 0.0 | 0 | 0.0 | 0 | 0 | **0** | **0.0** |
| skin rash | 0 | 0.0 | 1 | 1.3 | 0 | 0.0 | **1** | **0.3** | 0 | 0.0 | 0 | 0.0 | 0 | 0.0 | **0** | **0.0** | 0 | 0.0 | 1 | 2.4 | 0 | 0 | **1** | **0.7** |
| anger or aggression | 1 | 0.4 | 0 | 0.0 | 0 | 0.0 | **1** | **0.3** | 1 | 0.8 | 0 | 0.0 | 0 | 0.0 | **1** | **0.6** | 0 | 0.0 | 0 | 0.0 | 0 | 0 | **0** | **0.0** |
| bradycardia | 0 | 0.0 | 1 | 1.3 | 0 | 0.0 | **1** | **0.3** | 0 | 0.0 | 1 | 2.6 | 0 | 0.0 | **1** | **0.6** | 0 | 0.0 | 0 | 0.0 | 0 | 0 | **0** | **0.0** |
| bruising | 1 | 0.4 | 0 | 0.0 | 0 | 0.0 | **1** | **0.3** | 0 | 0.0 | 0 | 0.0 | 0 | 0.0 | **0** | **0.0** | 1 | 0.9 | 0 | 0.0 | 0 | 0 | **1** | **0.7** |
| difficulty talking | 0 | 0.0 | 1 | 1.3 | 0 | 0.0 | **1** | **0.3** | 0 | 0.0 | 1 | 2.6 | 0 | 0.0 | **1** | **0.6** | 0 | 0.0 | 0 | 0.0 | 0 | 0 | **0** | **0.0** |
| feeling of fullness in the ears | 0 | 0.0 | 0 | 0.0 | 1 | 16.7 | **1** | **0.3** | 0 | 0.0 | 0 | 0.0 | 1 | 20.0 | **1** | **0.6** | 0 | 0.0 | 0 | 0.0 | 0 | 0 | **0** | **0.0** |
| hallucinations | 0 | 0.0 | 1 | 1.3 | 0 | 0.0 | **1** | **0.3** | 0 | 0.0 | 1 | 2.6 | 0 | 0.0 | **1** | **0.6** | 0 | 0.0 | 0 | 0.0 | 0 | 0 | **0** | **0.0** |
| increase in sexual desire | 1 | 0.4 | 0 | 0.0 | 0 | 0.0 | **1** | **0.3** | 1 | 0.8 | 0 | 0.0 | 0 | 0.0 | **1** | **0.6** | 0 | 0.0 | 0 | 0.0 | 0 | 0 | **0** | **0.0** |
| increased bowel movements | 1 | 0.4 | 0 | 0.0 | 0 | 0.0 | **1** | **0.3** | 1 | 0.8 | 0 | 0.0 | 0 | 0.0 | **1** | **0.6** | 0 | 0.0 | 0 | 0.0 | 0 | 0 | **0** | **0.0** |
| increased glucose level | 0 | 0.0 | 1 | 1.3 | 0 | 0.0 | **1** | **0.3** | 0 | 0.0 | 1 | 2.6 | 0 | 0.0 | **1** | **0.6** | 0 | 0.0 | 0 | 0.0 | 0 | 0 | **0** | **0.0** |
| loss of consciousness | 0 | 0.0 | 1 | 1.3 | 0 | 0.0 | **1** | **0.3** | 0 | 0.0 | 1 | 2.6 | 0 | 0.0 | **1** | **0.6** | 0 | 0.0 | 0 | 0.0 | 0 | 0 | **0** | **0.0** |
| pins and needles sensation | 1 | 0.4 | 0 | 0.0 | 0 | 0.0 | **1** | **0.3** | 0 | 0.0 | 0 | 0.0 | 0 | 0.0 | **0** | **0.0** | 1 | 0.9 | 0 | 0.0 | 0 | 0 | **1** | **0.7** |
| strange dreams | 0 | 0.0 | 1 | 1.3 | 0 | 0.0 | **1** | **0.3** | 0 | 0.0 | 1 | 2.6 | 0 | 0.0 | **1** | **0.6** | 0 | 0.0 | 0 | 0.0 | 0 | 0 | **0** | **0.0** |
| thought of suicide | 1 | 0.4 | 0 | 0.0 | 0 | 0.0 | **1** | **0.3** | 1 | 0.8 | 0 | 0.0 | 0 | 0.0 | **1** | **0.6** | 0 | 0.0 | 0 | 0.0 | 0 | 0 | **0** | **0.0** |
| unsteadiness on feet | 0 | 0.0 | 1 | 1.3 | 0 | 0.0 | **1** | **0.3** | 0 | 0.0 | 1 | 2.6 | 0 | 0.0 | **1** | **0.6** | 0 | 0.0 | 0 | 0.0 | 0 | 0 | **0** | **0.0** |
| unusual or uncontrolled body movement | 0 | 0.0 | 0 | 0.0 | 1 | 16.7 | **1** | **0.3** | 0 | 0.0 | 0 | 0.0 | 1 | 20.0 | **1** | **0.6** | 0 | 0.0 | 0 | 0.0 | 0 | 0 | **0** | **0.0** |
| urinary retention | 0 | 0.0 | 1 | 1.3 | 0 | 0.0 | **1** | **0.3** | 0 | 0.0 | 1 | 2.6 | 0 | 0.0 | **1** | **0.6** | 0 | 0.0 | 0 | 0.0 | 0 | 0 | **0** | **0.0** |
| water retention | 1 | 0.4 | 0 | 0.0 | 0 | 0.0 | **1** | **0.3** | 0 | 0.0 | 0 | 0.0 | 0 | 0.0 | **0** | **0.0** | 1 | 0.9 | 0 | 0.0 | 0 | 0 | **1** | **0.7** |
| other | 23 | 9.9 | 8 | 10.1 | 2 | 33.3 | **33** | **10.4** | 15 | 12.0 | 5 | 13.2 | 1 | 20.0 | **21** | **12.5** | 8 | 7.4 | 3 | 7.3 | 1 | 100 | **12** | **8.0** |

# Appendix 6. Potential medication-related adverse effects experienced by antidepressant and antihypertensive users based on expanded, therapeutic class-targeted codeset

| Adverse Effect | Frequency (N) | Percent (%) |
| --- | --- | --- |
| Antidepressant users with potential ADE (N=11) | | |
| Insomnia | 4 | 36.36 |
| Diarrhea | 2 | 18.18 |
| Headache | 2 | 18.18 |
| Dizziness | 1 | 9.09 |
| Fatigue | 1 | 9.09 |
| Ischemic Stroke | 1 | 9.09 |
| Antihypertensive users with potential ADE (N=51)* | | |
| Hyperglycemia | 21 | 35.59 |
| Dyspnea | 10 | 16.95 |
| Cough | 5 | 8.47 |
| Peripheral Edema | 5 | 8.47 |
| Fatigue | 3 | 5.08 |
| Constipation | 2 | 3.39 |
| Dizziness/Vertigo | 2 | 3.39 |
| Hyperlipidemia | 2 | 3.39 |
| Hypokalemia | 2 | 3.39 |
| Renal Impairment | 2 | 3.39 |
| Headache | 1 | 1.69 |
| Hepatotoxicity | 1 | 1.69 |
| Hyperkalemia | 1 | 1.69 |
| Hypotension | 1 | 1.69 |
| Psoriasis | 1 | 1.69 |

Abbreviations: ADE=Adverse Drug Event
*The cumulative frequency of adverse effects exceeds the number of patients experiencing said adverse effects because some patients experienced more than 1 relevant adverse effect during the follow-up period.

**Appendix 7. Details of treatment changes among patients with a potential ADE based on e-prescribing data**

| **Stop/Change to Study Medication** | **Patients with potential ADE based on e-prescribing data** | | |
| --- | --- | --- | --- |
|  | **Overall**  **N=40** | **Patients on antidepressants**  **N=20** | **Patients on antihypertensives**  **N=20** |
| **Stopped** | N=38 (95.0%) | N=20 (100%) | N=18 (90.0%) |
| Safety: Adverse Drug Reaction | 26 (68.4%) | 14 (70.0%) | 12 (66.7%) |
| Ineffective Treatment | 12 (31.6%) | 6 (30.0%) | 6 (33.3%) |
| **Dose Changed** | N=2 (5.0%) | N=0 (0%) | N=2 (10.0%) |
| Safety: Adverse Drug Reaction | 2 (100%) | 0 (0%) | 2 (100%) |
| Ineffective Treatment | 0 (0%) | 0 (0%) | 0 (0%) |

**Appendix 8. Characteristics of patients who reported all, none, or some of the symptoms they indicated in the interview to their physician**

|  | **Reported all symptoms to physician  (N=79)** | **Reported no symptoms to physician  (N=233)** | **Reported some symptoms to physician (N=6)** |
| --- | --- | --- | --- |
| Sex (male) | 20 (25.3%) | 69 (29.6%) | 1 (16.7%) |
| Age, mean (SD) | 66.3 (12.2) | 61.5 (14.9) | 64.2 (8.7) |
| Study Medication |  |  |  |
| Antihypertensive | 41 (51.9%) | 108 (46.4%) | 1 (16.7%) |
| Antidepressant | 38 (48.1%) | 125 (53.6%) | 5 (83.3%) |

**Appendix 9. Severity of symptoms experienced, as reported during interviews, by patients who reported all, none, or some of their symptoms to their physician**

| **Severity** | **All symptoms N=658*** | **Symptoms of patients who reported all to physician N=152** | **Symptoms of patients who reported none to physician N=474** | **Symptoms of patients who reported some to physician**  **N=31** |
| --- | --- | --- | --- | --- |
| Minimal | 71 (10.8%) | 9 (5.9%) | 55 (11.6%) | 7 (22.6%) |
| Mild | 117 (17.8%) | 23 (15.1%) | 88 (18.6%) | 6 (19.4%) |
| Moderate | 246 (37.4%) | 40 (26.3%) | 193 (40.7%) | 13 (41.9%) |
| Severe | 159 (24.2%) | 56 (36.8%) | 100 (21.1%) | 3 (9.7%) |
| Very Severe | 51 (7.8%) | 23 (15.1%) | 28 (5.9%) | 0 (0%) |
| Doesn’t Apply | 13 (2.0%) | 1 (0.7%) | 10 (2.1%) | 2 (6.5%) |

*One symptom is missing information on severity
